# Supplementary material for: Dose-dependent oral glucocorticoid cardiovascular risks in people with immune-mediated inflammatory diseases: A population-based cohort study
Source: PLoS Med. 2020 Dec 3;17(12):e1003432. doi: 10.1371/journal.pmed.1003432 (PMC7714202; doi:10.1371/journal.pmed.1003432)
Supplement: S1 Text — (DOCX) [file pmed.1003432.s001.docx]

##### Dose-dependent oral glucocorticoid cardiovascular risks in people with immune-mediated inflammatory diseases: a population-based cohort study

Mar Pujades-Rodriguez, Ann W Morgan, Richard M Cubbon, Jianhua Wu

**S1 TEXT**

**SUPPLEMENTARY METHODS**

**Sources of data**

The dataset analysed included individual patient electronic health records from three data sources: the Clinical Practice Research Datalink (CPRD-GOLD; http://www.cprd.com/, the Hospital Episodes Statistics (HES; http://www.hscic.gov.uk/hes) and the Office of National Statistics (ONS; http://www.ons.gov.uk/ons). Data linkage was performed by CPRD. The CPRD dataset contains complete information on all the prescriptions issued to the patients registered in their primary care general practices. CPRD also gathers health data recorded and coded during primary care contacts, including demographic and lifestyle information, symptoms, medical diagnoses, clinical and laboratory examinations and medical procedures. CPRD data were coded with the Read classification system (version 2), a hierarchical clinical classification containing over 96,00 codes. Accuracy and completeness of data collected in CPRD general practices are regularly audited. Studies have shown that, compared with the UK census, patients included in the CPRD dataset are broadly representative of the UK population, in terms of age, sex and ethnicity(1, 2). Compared to participants in the Health Survey for England (household-based population survey), CPRD patients have also been shown to be comparable in terms of distribution of body mass index. Multiple validation studies of data collected in CPRD (e.g. clinical diagnoses), primarily evaluating their positive predicted value (PPV), have shown high PPV estimates; and have also reported estimates of incidence that are similar to other UK data sources(3-6). HES provides information about medical diagnoses made during all elective and emergency hospital admission across all National Health Service hospitals in England. HES and ONS data are coded using the 10^th^ revision of the International Classification of Diseases (ICD-10) classification system. ONS data collected prior to 2000 is coded using the 9th revision of the ICD.

**Covariate definition**

To create the baseline covariates used in the analysis we used the following definitions:

- medication use: ≥1 prescriptions issued to the patient within 1 year prior to the start of follow-up in CPRD
- smoking status: the closest smoking status recorded within 1 year prior to the start of follow-up in CPRD. We then categorised as ‘ex-smokers’ patients who had non-smoker status at baseline but for whom ‘current smoking’ had been recorded at any time prior to the follow-up start.
- comorbidities: a diagnosis recorded at any time prior to the start of follow-up in CPRD or HES
- quantitative biomarkers (e.g. body mass index): the closest measurement recorded within 1 year prior to the start of follow-up in CPRD
- number of hospital visits: the total number of hospital admissions within 1 year prior to the start of follow-up in HES

**Definition of flare**

To account for increases in cardiovascular risk during periods of flare or active disease, we divided the study follow-up of each patient into periods of active and inactive disease. We then created a time-variant variable that had a value of ‘0’ during periods of inactivity and ‘1’ during periods of active disease or flare. We defined a flare based on the daily glucocorticoid dose prescribed and, for patients with rheumatoid arthritis, polymyalgia rheumatic, giant cell arteritis and inflammatory bowel disease, also based on the level of c-reactive protein (CRP) and erythrocyte sedimentation rate (ESR) in the blood. We did not consider CRP and ESR levels for the definition of flare in patients with systemic lupus erythematosus and vasculitis because patients with these diseases can have flares without increases of these biomarkers. Periods of flare started when there was a sudden increase in the prescribed prednisolone-equivalent daily dose by >5 mg (or >10 mg) that lasted over 3 weeks; when the CRP value was ≥10 mg/mL; or the ESR value was ≥30 mm/h. The flare ended when the glucocorticoid daily dose prescribed was reduced to <5 mg (or <10 mg) and/or the values of biomarkers decreased to normal (CRP <10 mg/mL and/or ESR <30 mm/h).

**Multiple imputation of glucocorticoid dose and covariates**

Imputation of exposure and covariate missing data took place in 2 consecutive steps.

**Step 1: Multiple imputation of glucocorticoid dose**

The median number of prescriptions issued per patient during the study follow-up was 16 (IQR 4-48) and the median duration between these prescriptions 31 days (IQR 22-56). The total number of tablets prescribed, which was available for all patients and prescriptions, allowed us to calculate the duration of each prescription. To account for ≥2 consecutive prescriptions being the same episode of glucocorticoid dose exposure, we allowed a grace period of 90 days between refills. The median number of prescriptions with missing dose per patient, which generally corresponds to tapering periods, was 10.3% (IQR 0.0-50.1%). Missing daily glucocorticoid dose appeared to be missing at random after adjusting for major confounders (e.g. age, underlying disease, disease duration). It was therefore replaced through generation of 5 datasets using multiple imputation with chained equations (MICE package in R 3.3.1).

The imputation model specifications and variable missingness are shown in the table below.

| **Variable** | **Variable Type** | **Missing (%)** | **Imputation method** |
| --- | --- | --- | --- |
| Patient indicator | Continuous, non-normal | 0 | Predictor/Auxiliary variable |
| Family practice indicator | Continuous, non-normal | 0 | Predictor/Auxiliary variable |
| Age | Continuous, non-normal | 0 | Predictor/Auxiliary variable |
| Sex | Binary | 0 | Predictor/Auxiliary variable |
| Ethnicity | Category | 8.4 | Polytomous logistic regression |
| Index of multiple deprivation | Continuous, non-normal | 0 | Predictor/Auxiliary variable |
| Body mass index | Continuous, non-normal | 59.1 | Predictive mean matching |
| Underlying immune-mediated diseases (first recorded disease) | Category | 0 | Predictor/Auxiliary variable |
| Duration of underlying inflammatory disease (for first recorded disease) | Category | 0 | Predictor/Auxiliary variable |
| Daily oral prednisolone-equivalent glucocorticoid dose | Continuous, non-normal | 34.1* | Predictive mean matching |
| Type of oral glucocorticoid** | Category | 0 | Predictor/Auxiliary variable |
| Time between the follow-up start and the date of glucocorticoid prescription | Continuous, non-normal | 0 | Predictor/Auxiliary variable |
| Diabetes | Binary | 0 | Predictor/Auxiliary variable |
| Prescribed non-oral glucocorticoids (one variable for each route of administration: inhaled, nasal, intramuscular/intra-articular, topical, recatal) | Binary | 0 | Predictor/Auxiliary variable |

* This indicates the total percentage of missingness in relation to the total number of prescriptions, not in relation to the total number of patients or to the total number of glucocorticoid exposure episodes/periods. The median number of prescriptions with missing dose per patient was 10.3%. ** Oral glucocorticoid drugs were: prednisolone, prednisone, budenoside, beclomethasone, betamethasone, deflazacort, dexamethasone, hydrocortisone, triamcinolone, cortisone and methylprednisolone

Were generated 5 multiply imputed datasets. We compared the density of the imputed data for each imputed dataset against the density of the observed data. We used the Kolmogorov-Smirnov test to compare the difference between the observations and the imputed values

**Step2: Multiple imputation of covariates for adjustment**

We performed the second imputation step for each of the 5 dose-imputed datasets separately. Risk factor data appeared to be missing at random after adjusting for major confounders (e.g. age, sex, and underlying disease conditions). Hence, we implemented multiple imputation via chained equations using the MICE package in R 3.3.1, to replace missing values for risk factor variables. The imputation model specifications and extent of missingness are shown in the table below.

| **Variable** | **Variable Type** | **Missing (%)** | **Imputation method** |
| --- | --- | --- | --- |
| Age | Continuous, non-normal | 0 | Predictor/Auxiliary variable |
| Sex | Binary | 0 | Predictor/Auxiliary variable |
| Ethnicity | Category | 0 (Imputed) | Predictor/Auxiliary variable |
| Underlying immune-mediated diseases | Category | 0 | Predictor/Auxiliary variable |
| Body mass index | Continuous, non-normal | 0 (Imputed) | Predictor/Auxiliary variable |
| Index of multiple deprivation | Continuous, non-normal | 0 | Predictor/Auxiliary variable |
| Number of hospital admissions | Continuous, non-normal | 11.6 | Predictive mean matching |
| Oral glucocorticoid daily dose | Continuous, non-normal | 0 (Imputed) | Predictor/Auxiliary variable |
| Systolic blood pressure | Continuous, non-normal | 36.6 | Predictive mean matching |
| Total cholesterol level | Continuous, non-normal | 74.9 | Predictive mean matching |
| High-density lipoprotein cholesterol | Continuous, non-normal | 84.4 | Predictive mean matching |
| Low-density lipoprotein cholesterol | Continuous, non-normal | 80.4 | Predictive mean matching |
| C-reactive protein | Continuous, non-normal | 68.4 | Predictive mean matching |
| Creatinine | Continuous, non-normal | 47.7 | Predictive mean matching |
| Smoking status | Binary | 24.9 | Default imputation (missing set to no) |
| Diabetes | Binary | 0 | Predictor/Auxiliary variable |
| Cancer | Binary | 0 | Predictor/Auxiliary variable |
| Asthma | Binary | 0 | Predictor/Auxiliary variable |
| Chronic obstructive pulmonary disease | Binary | 0 | Predictor/Auxiliary variable |
| Renal disease | Binary | 0 | Predictor/Auxiliary variable |
| Hypertension diagnosis | Binary | 0 | Predictor/Auxiliary variable |
| Prescribed non-oral glucocorticoids | Binary | 0 | Predictor/Auxiliary variable |
| DMARDs | Binary | 0 | Predictor/ Auxiliary variable |
| NSAIDs | Binary | 0 | Predictor/ Auxiliary variable |

Abbreviations: DMARDs, disease-modifying anti-rheumatic drugs; NSAIDs, non-steroidal anti-inflammatory drugs.

We generated 5 multiply imputed datasets for each of the 5 dose-imputed datasets. We compared the density of the imputed data for each imputed dataset against the density of the observed data. We used the Kolmogorov-Smirnov test to compare the difference between the observations and the imputed values.

**Propensity score for prescribing information**

For each patient, a propensity score was calculated via a logistic regression model with prescribed oral glucocorticoid treatment specified as the binary outcome and standard weights. The predictive variables included in the model were: baseline age, sex, index of multiple deprivation, smoking status, ethnicity, body mass index, comorbidities (diabetes, diagnosed hypertension, cancer, asthma, chronic obstructive pulmonary disease and advanced chronic kidney disease), biomarkers (total cholesterol, high- and low-density lipoprotein cholesterol, c-reactive protein, creatinine; modelled as cubic spline), and the type of immune-mediated inflammatory disease diagnosed (included as separate binary variables). The propensity score was then used to adjust the Cox proportional hazard model to balance covariates between exposure groups in order to control the potential residual confounding by glucocorticoid indication.

**REFERENCES**

1. Herrett E, Gallagher AM, Bhaskaran K, Forbes H, Mathur R, van Staa T, et al. Data Resource Profile: Clinical Practice Research Datalink (CPRD). Int J Epidemiol. 2015;44(3):827-36.

2. Mathur R, Bhaskaran K, Chaturvedi N, Leon DA, vanStaa T, Grundy E, et al. Completeness and usability of ethnicity data in UK-based primary care and hospital databases. J Public Health (Oxf). 2014;36(4):684-92.

3. Meier CR, Napalkov PN, Wegmuller Y, Jefferson T, Jick H**.** Population-based study on incidence, risk factors, clinical complications and drug utilisation associated with influenza in the United Kingdom. Eur J Clin Microbiol Infect Dis. 2000;19(11):834-42.

4. Ronquist G, Rodriguez LA, Ruigomez A, Johansson S, Wallander MA, Frithz G, et al. Association between captopril, other antihypertensive drugs and risk of prostate cancer. Prostate. 2004;58(1):50-6.

5. Ryan R, Majeed A**.** Prevalence of treated hypertension in general practice in England and Wales, 1994 and 1998. Health Stat Q. 2002;16:14-8.

6. van Staa TP, Dennison EM, Leufkens HG, Cooper C**.** Epidemiology of fractures in England and Wales. Bone. 2001;29(6):517-22.
