# Supplementary material for: Dose-dependent oral glucocorticoid cardiovascular risks in people with immune-mediated inflammatory diseases: A population-based cohort study
Source: PLoS Med. 2020 Dec 3;17(12):e1003432. doi: 10.1371/journal.pmed.1003432 (PMC7714202; doi:10.1371/journal.pmed.1003432)
Supplement: S1 Table — Table A. Definition of immune-mediated inflammatory diseases by data source. Table B. Prednisolone-equivalent dose conversion factors for glucocorticoids. Table C. Definition of cardiovascular outcomes by data source. Table D. Observation time and incidence rates of cardiovascular diseases by sex. Table E. Cumulative incidence estimates of cardiovascular diseases per level of current daily and cumulative oral glucocorticoid prednisolone-equivalent dose by type of immune-mediated inflammatory disease. Table F. Cumulative incidence estimates of cardiovascular diseases per level of current daily and cumulative oral glucocorticoid prednisolone-equivalent dose by type of immune-mediated inflammatory disease in men. Table G. Cumulative incidence estimates of cardiovascular diseases per level of current daily and cumulative oral glucocorticoid prednisolone-equivalent dose in women. Table H. Associations between time-variant oral glucocorticoid prednisolone-equivalent dose and incident all-cause cardiovascular disease by immune-mediated inflammatory disease, reported as crude hazard ratios with 95% CI. Table I. Association between time-variant oral glucocorticoid dose and incident cardiovascular disease in patients with 6 immune-mediated inflammatory diseases from complete case analysis. Table J. Association between time-variant oral glucocorticoid dose and incident all-cause cardiovascular disease by type of immune-mediated inflammatory disease from analysis in which missing covariate values were coded as a separate category. Table K. Association between time-variant oral glucocorticoid dose and incident all-cause cardiovascular disease by type of immune-mediated inflammatory disease from analysis in which biomarkers with over 60% missing data were excluded. Table L. Association between time-variant oral glucocorticoid dose and 6 incident cardiovascular diseases in patients with 6 immune-mediated inflammatory diseases from analysis in which missing covariate values were co [file pmed.1003432.s002.docx]

##### Dose-dependent oral glucocorticoid cardiovascular risks in people with immune-mediated inflammatory diseases: a population-based cohort study

Mar Pujades-Rodriguez, Ann W Morgan, Richard M Cubbon, Jianhua Wu

**S1 TABLE**

Table A. Definition of immune-mediated inflammatory diseases by data source

Table B. Prednisolone-equivalent dose conversion factors for glucocorticoids

Table C. Definition of cardiovascular outcomes by data source

Table D. Observation time and incidence rates of cardiovascular diseases by sex

Table E. Cumulative incidence estimates of cardiovascular diseases per level of current daily and cumulative oral glucocorticoid prednisolone-equivalent dose by type of immune-mediated inflammatory disease

Table F. Cumulative incidence estimates of cardiovascular diseases per level of current daily and cumulative oral glucocorticoid prednisolone-equivalent dose by type of immune-mediated inflammatory disease in men

Table G. Cumulative incidence estimates of cardiovascular diseases per level of current daily and cumulative oral glucocorticoid prednisolone-equivalent dose in women

Table H. Associations between time variant oral glucocorticoid prednisolone-equivalent dose and incident all-cause cardiovascular disease by immune-mediated inflammatory disease, reported as crude hazard ratios with 95% CI

Table I. Association between time variant oral glucocorticoid dose and incident cardiovascular disease in patients with 6 immune-mediated inflammatory diseases from complete case analysis

Table J. Association between time variant oral glucocorticoid dose and incident all-cause cardiovascular disease by type of immune-mediated inflammatory disease from analysis in which missing covariate values were coded as a separate category

Table K. Association between time variant oral glucocorticoid dose and incident all-cause cardiovascular disease by type of immune-mediated inflammatory disease from analysis in which biomarkers with over 60% missing data were excluded

Table L. Association between time variant oral glucocorticoid dose and 6 incident cardiovascular diseases in patients with 6 immune-mediated inflammatory diseases from analysis in which missing covariate values were coded as a separate category

Table M. Association between time variant oral glucocorticoid dose and 6 incident cardiovascular disease in patients with 6 immune-mediated inflammatory diseases from analysis in which biomarkers with over 60% missing data were excluded

Table N. Association between time variant oral glucocorticoid dose and incident all-cause cardiovascular disease by type of immune-mediated inflammatory disease, restricted to patients with newly diagnosed immune-mediated inflammatory disease

Table O. Association between time variant oral glucocorticoid dose and 6 incident cardiovascular diseases in patients with six immune-mediated inflammatory diseases, restricted to patients with newly diagnosed immune-mediated inflammatory disease

Table P. Association between time variant oral glucocorticoid dose and 6 incident cardiovascular diseases in patients with six immune-mediated inflammatory diseases, restricted to patients diagnosed with immune-mediated inflammatory disease within 2 years

Table Q. Association between time variant oral glucocorticoid dose and 6 incident cardiovascular diseases in patients with six immune-mediated inflammatory diseases, restricted to patients diagnosed with immune-mediated inflammatory diseases for over 2 years

Table R. Association between time variant oral glucocorticoid dose and 6 incident cardiovascular diseases in patients with six immune-mediated inflammatory diseases, adjusted for periods of flare during follow-up (defined by biomarker or 5 mg daily dose increase)

Table S. Association between time variant oral glucocorticoid dose and 6 incident cardiovascular diseases in patients with six immune-mediated inflammatory diseases, adjusted for periods of flare during follow-up (defined by biomarker or 10 mg daily dose increase)

Table T. Associations between time variant oral glucocorticoid prednisolone-equivalent dose and incident all-cause cardiovascular disease by immune-mediated inflammatory disease, according to the number of years of exposure considered prior to follow-up start [Additional sensitivity analysis]

Table U. Associations between time variant oral glucocorticoid prednisolone-equivalent dose and incident all-cause cardiovascular disease by immune-mediated inflammatory disease, adjusted for propensity score for prescribing indication [Additional sensitivity analysis]

Table V. Summary of the methodology and major findings of previous studies investigating the association between glucocorticoid dose and cardiovascular diseases

Note: Estimates of dose-response associations for specific types of CVD in patients with systemic lupus erythematosus are not presented because of the small numbers of events available for the analysis

**Table A. Definitions of immune-mediated inflammatory diseases by data source**

| **Inflammatory chronic disease** | **CPRD – Read codes** | **HES-ICD 10 hospital diagnoses** |
| --- | --- | --- |
| **Inflammatory bowel disease** | J40..12, J4z2.00 + 17 Read codes for Crohn’s disease + 8 Read codes for regional enteritis  J41y.00, J41y100, J41yz00, J57yA00, J57y900 + 16 Read codes for ulcerative colitis  4 Read codes for inflammatory bowel disease | M07.4, M07.5, M09.1, M09.2, K50.0, K50.1, K50.8, K50.9, M51 |
| **Polymyalgia rheumatica and giant cell arteritis** | G755100, G755000, G755.00, N200.00, Nyu4100, G755z00, G755200, N20..00, N20..11 | M31.5, M31.6, M35.3 |
| **Rheumatoid arthritis** | 2G27.00, 66H..13, G5y8.00, G5yA.00, H570.00, N005.00, N040N00, N040Q00, N040R00, N041.00, N042.00, N042100, N042200, N042z00, N04y000, N04y011, N04y200 + 35 Read codes for rheumatoid arthritis | I52.8, J99.0, M05, M06 |
| **Systemic lupus erythematosus** | K01x411, M154.00, M154z00, M154700 + 15 Read codes for systemic lupus erythematosus | M32 |
| **Vasculitis** | AD61.00, C332100, D310100, D310000, D310011, F371100, F396300, F421E00, G75..00, G750.00, G757.00, G757.12, G751000, G75z.00, G758.00, G754.11, G754.00, G752112, G752111, G76B.00, K01x300, K425200, N012.00, N012000, N012011, N012700, N012x00, N040N00 | D69.0, H35.0, M05, M30, M31.3, M31.4, M31.7, M35.2 |

**Table B. Prednisolone-equivalent dose conversion factors for glucocorticoids**

| **Glucocorticoid*** | **10mg prednisolone-equivalent (in mg)** |
| --- | --- |
| Betamethasone | 1.5 |
| Budenoside | 1.09 |
| Cortisone | 50 |
| Deflazacort | 12 |
| Dexamethasone | 1.5 |
| Hydrocortisone | 40 |
| Methylprednisolone | 8 |
| Prednisone | 10 |
| Triamcinolone | 8 |

* Overall, 96.4% of all prescriptions issued to cohort patients were for prednisolone and only 0.2% of all prescriptions for oral glucocorticoids were for budesonide.

**Table C. Definitions of cardiovascular outcomes by data source**

| **Endpoint** | **CPRD – Read codes** | **HES-ICD 10 hospital diagnoses** | **ONS-ICD 10 causes of death** | **ONS-ICD 9 causes of death** |
| --- | --- | --- | --- | --- |
| **Atrial fibrillation** | G573000 + 7 other Read codes for paroxysmal atrial fibrillation, persistent atrial fibrillation and atrial flutter. | I48.0 | Not used | Not used |
| **Heart failure** | G58..00 + 40 other Read codes for heart failure | I11.0, I13.0, I13.2, I50 | I11.0, I13.0, I13.2, I50 | 415.0, 428.0, 428.1, 428.2, 428.3, 428.3, 428.4, 428.9 |
| **Myocardial infarction** | G30X000, G307100, G30..15 + 40 other Read codes | I21, I22 | I20-I25 | 410 |
| **Cerebrovascular disease** | G64..11 + 9 other Read codes for ischaemic stroke  G60X.00 + 2 other Read codes for subarachnoid haemorrhage  G61..00 + 16 other Read codes for intracerebral haemorrhage  G66..00 + 14 other Read codes | I63  I60  I61  I64, G46.3-G46.7 | I63  I60  I61  I64, G46.3-G46.7 | 433.0, 433.1, 433.2, 433.3, 433.8, 433.9, 434.0, 434.1, 434.9  430.0  431.0  436.0, 437.0, 437.9 |
| **Peripheral arterial disease** | 63 codes for Lower limb peripheral arterial disease diagnosis, 136 Read codes for procedures for peripheral arterial disease, 2 Read codes for abnormal lower limb angiogram. | I73.1, I73.8, I73.9, I74.3, I74.4, I74.5 | I73.1, I73.8, I73.9, I74.3, I74.4, I74.5 | 443.1, 443.8, 443.9, 444.2, 444.8, 453.4 |
| **Abdominal aortic aneurysm** | G714.00 + 12 other Read codes. | I71.3, I71.4, I71.5, I71.6, I71.8, I71.9 | I71.3, I71.4, I71.5, I71.6, I71.8, I71.9 | 441.3, 441.4, 441.5, 441.6, 441.6, 441.7, 441.9 |

Abbreviations: HES, Hospital Episode Statistics; ICD, International Classification of Diseases (versions 9 and 10); ONS, Office for National Statistics

**Table D. Observation time and incidence rates of cardiovascular diseases by sex**

| All patients | **All CVDs** | **Atrial fibrillation** | **Heart failure** | **Myocardial infarction** | **Cerebrovascular disease** | **Peripheral arterial disease** | **Abdominal aortic aneurysm** |
| --- | --- | --- | --- | --- | --- | --- | --- |
| **Incidence cases, n (%)** | 13,426 | 6,013 | 4,727 | 2,809 | 1,972 | 2,947 | 698 |
| **Total person-years** | 541,655 | 566,291 | 573,937 | 576,994 | 577,124 | 577,852 | 583,361 |
| **Time at risk (years), median [IQR]** | 5.0 [2.0-6.2] | 5.0 [2.0-6.5] | 6.0 [2.0-6.5] | 6.0 [2.0-6.6] | 6.0 [2.0-6.6] | 6.0 [2.0-6.6] | 6.0 [2.0-6.6] |
| **Incidence per 1,000 person-years (95% CI)** |  |  |  |  |  |  |  |
| **Overall** | 24.8 (24.4-25.2) | 10.6 (10.4-10.9) | 8.2 (8.0-8.5) | 4.9 (4.7-5.1) | 3.4 (3.3-3.6) | 5.1 (4.9-5.3) | 1.2 (1.1-1.3) |
| **Current daily PED** |  |  |  |  |  |  |  |
| Non-use | 18.5 (18.1-18.9) | 7.9 (7.7- 8.2) | 5.8 (5.5- 6.0) | 3.6 (3.5- 3.8) | 2.5 (2.4-2.7) | 4.2 (4.0- 4.4) | 0.9 (0.8-1.0) |
| >0.0-4.9 mg | 46.0 (44.0-48.0) | 20.4 (19.0-21.9) | 15.6 (14.4-16.9) | 8.3 (7.4- 9.3) | 6.2 (5.5-7.1) | 8.4 (7.5- 9.3) | 2.6 (2.1-3.3) |
| 5.0-14.9 mg | 49.9 (48.3-51.4) | 20.4 (19.4-21.5) | 17.7 (16.7-18.7) | 10.2 (9.5-11.0) | 7.0 (6.4-7.6) | 8.7 (8.1- 9.5) | 2.3 (1.9-2.6) |
| 15.0-24.9 mg | 61.1 (56.6-65.8) | 24.9 (21.9-28.2) | 22.6 (19.8-25.7) | 9.7 (7.8-11.9) | 7.5 (5.9-9.5) | 9.0 (7.2-11.1) | 2.1 (1.3-3.2) |
| ≥25 mg | 45.6 (42.1-49.2) | 20.1 (17.8-22.8) | 18.5 (16.2-20.9) | 9.0 (7.4-10.9) | 5.5 (4.3-7.1) | 6.4 (5.0- 8.0) | 1.2 (0.7-2.1) |
| **Cumulative PED** |  |  |  |  |  |  |  |
| Non-use | 19.9 (19.3-20.5) | 8.2 (7.8- 8.6) | 5.8 (5.5- 6.1) | 4.0 (3.7-4.2) | 2.9 (2.7-3.1) | 4.2 (3.9-4.5) | 0.9 (0.8-1.1) |
| >0.0-959.9 mg | 22.2 (21.3-23.2) | 9.3 (8.7-10.0) | 7.5 (6.9- 8.1) | 4.3 (3.8-4.7) | 2.9 (2.6-3.3) | 4.7 (4.2-5.2) | 1.0 (0.8-1.3) |
| 960-3,054.9 mg | 29.0 (27.9-30.1) | 12.7 (12.0-13.5) | 9.3 (8.7-10.0) | 5.1 (4.6-5.6) | 3.6 (3.2-4.1) | 5.9 (5.4-6.5) | 1.4 (1.1-1.7) |
| 3,055-7,299.9 mg | 32.7 (31.6-33.8) | 14.2 (13.4-15.0) | 11.0 (10.4-11.7) | 6.1 (5.6-6.7) | 4.2 (3.8-4.7) | 6.7 (6.2-7.3) | 1.5 (1.3-1.8) |
| ≥7,300 mg | 26.4 (25.5-27.2) | 11.5 (11.0-12.1) | 9.9 (9.4-10.4) | 5.7 (5.3-6.1) | 3.9 (3.6-4.2) | 5.1 (4.7-5.5) | 1.4 (1.2-1.6) |
| **Men** | **All CVDs** | **Atrial fibrillation** | **Heart failure** | **Myocardial infarction** | **Cerebrovascular disease** | **Peripheral arterial disease** | **Abdominal aortic aneurysm** |
| **Incidence cases, n (%)** | 4,739 | 2,077 | 1,514 | 1,163 | 745 | 916 | 390 |
| **Total person-years** | 180,317 | 189,525 | 192,808 | 192,602 | 193,351 | 193,953 | 195,312 |
| **Time at risk (years), median [IQR]** | 5.0 [2.0-6.0] | 5.0 [2.0-6.3] | 5.0 [2.0-6.4] | 5.0 [2.0-6.4] | 5.0 [2.0-6.5] | 5.0 [2.0-6.5] | 6.0 [2.0-6.5] |
| **Incidence per 1,000 person-years (95% CI)** |  |  |  |  |  |  |  |
| **Overall** | 26.3 (25.6-27.0) | 11.0 (10.5-11.4) | 7.9 (7.5-8.3) | 6.0 (5.7-6.4) | 3.9 (3.6-4.1) | 4.7 (4.4-5.0) | 2.0 (1.8-2.2) |
| **Current daily PED** |  |  |  |  |  |  |  |
| Non-use | 19.9 (19.2-20.6) | 8.1 (7.7-8.6) | 5.2 (4.9-5.6) | 4.6 (4.3-4.9) | 3.0 (2.7-3.2) | 4.0 (3.7-4.3) | 1.5 (1.4-1.7) |
| >0.0-4.9 mg | 53.0 (49.0-57.3) | 23.2 (20.4-26.3) | 16.8 (14.5-19.4) | 10.1 (8.4-12.3) | 7.6 (6.0- 9.5) | 8.5 (6.8-10.4) | 4.4 (3.2-5.9) |
| 5.0-14.9 mg | 55.1 (52.2-58.1) | 21.9 (20.0-23.9) | 18.9 (17.2-20.8) | 13.5 (12.0-15.1) | 7.7 (6.6-9.0) | 7.6 (6.5-8.9) | 3.9 (3.1-4.8) |
| 15.0-24.9 mg | 66.4 (58.3-74.9) | 27.0 (21.9-33.1) | 24.0 (19.1-29.9) | 11.6 (8.4-16.1) | 9.1 (6.2-13.3) | 10.2 (7.0-14.3) | 5.2 (3.2-8.4) |
| ≥25 mg | 48.1 (42.3-54.5) | 23.5 (19.3-28.4) | 20.5 (16.6-25.2) | 10.5 (7.7-14.1) | 5.8 (3.8-8.9) | 4.0 (2.5-6.5) | 1.8 (0.8-3.8) |
| **Total cumulative PED** |  |  |  |  |  |  |  |
| Non-use | 21.5 (20.5-22.6) | 8.6 (8.0-9.3) | 5.4 (4.9-6.0) | 5.0 (4.5-5.6) | 3.3 (2.8-3.7) | 4.0 (3.5-4.4) | 1.5 (1.3-1.9) |
| >0.0-959.9 mg | 25.4 (23.6-27.3) | 10.4 (9.2-11.6) | 7.5 (6.5-8.6) | 5.5 (4.7-6.5) | 3.4 (2.8-4.2) | 4.6 (3.9-5.5) | 2.1 (1.6-2.7) |
| 960-3,054.9 mg | 31.3 (29.4-33.3) | 13.8 (12.5-15.2) | 8.7 (7.7-9.8) | 6.0 (5.1-6.9) | 4.2 (3.6-5.1) | 5.3 (4.5-6.1) | 2.7 (2.1-3.3) |
| 3,055-7,299.9 mg | 33.0 (31.1-35.0) | 13.7 (12.4-15.0) | 10.0 (9.0-11.3) | 7.5 (6.6-8.5) | 4.9 (4.1-5.7) | 5.8 (5.0-6.7) | 2.2 (1.7-2.8) |
| ≥7,300 mg | 27.2 (25.6-28.8) | 11.5 (10.5-12.6) | 10.3 (9.4-11.3) | 7.1 (6.3-8.0) | 4.2 (3.6-4.9) | 5.0 (4.3-5.7) | 2.1 (1.7-2.6) |
| **Women** | **All CVDs** | **Atrial fibrillation** | **Heart failure** | **Myocardial infarction** | **Cerebrovascular disease** | **Peripheral arterial disease** | **Abdominal aortic aneurysm** |
| **Incidence cases, n (%)** | 8,687 | 3,936 | 3,213 | 1,646 | 1,227 | 2,031 | 308 |
| **Total person-years** | 361,338 | 376,766 | 381,129 | 384,392 | 383,773 | 383,899 | 388,049 |
| **Time at risk (years), median [IQR]** | 5.0 [2.0-6.2] | 6.0 [2.0-6.5] | 6.0 [2.0-6.6] | 6.0 [2.0-6.6] | 6.0 [2.0-6.6] | 6.0 [2.0-6.6] | 6.0 [3.0-6.7] |
| **Incidence per 1,000 person-years (95% CI)** |  |  |  |  |  |  |  |
| **Overall** | 24.0 (23.6-24.5) | 10.4 (10.1-10.8) | 8.4 (8.1-8.7) | 4.3 (4.1-4.5) | 3.2 (3.0-3.4) | 5.3 (5.1-5.5) | 0.8 (0.7-0.9) |
| **Current daily PED** |  |  |  |  |  |  |  |
| Non-use | 17.7 (17.3-18.2) | 7.8 (7.5-8.1) | 6.0 (5.8-6.3) | 3.1 (2.9-3.3) | 2.3 (2.2-2.5) | 4.3 (4.1-4.5) | 0.6 (0.5-0.7) |
| >0.0-4.9 mg | 43.3 (41.0-45.7) | 19.3 (17.7-21.0) | 15.2 (13.8-16.7) | 7.6 (6.6- 8.7) | 5.7 (4.8-6.7) | 8.3 (7.3-9.5) | 2.0 (1.5-2.6) |
| 5.0-14.9 mg | 47.7 (45.9-49.5) | 19.8 (18.6-21.0) | 17.1 (16.0-18.3) | 8.7 (7.9- 9.6) | 6.6 (5.9-7.4) | 9.2 (8.4-10.1) | 1.5 (1.2-2.0) |
| 15.0-24.9 mg | 58.4 (52.9-64.1) | 23.8 (20.3-27.9) | 21.8 (18.5-25.7) | 8.7 (6.6-11.4) | 6.8 (4.9-9.2) | 8.4 (6.4-11.0) | 0.5 (0.2-1.6) |
| ≥25 mg | 44.2 (40.0-48.6) | 18.3 (15.5-21.4) | 17.3 (14.7-20.4) | 8.2 (6.3-10.4) | 5.4 (3.9-7.4) | 7.6 (5.9- 9.9) | 0.9 (0.4-2.0) |
| **Cumulative PED** |  |  |  |  |  |  |  |
| Non-use | 19.0 (18.3-19.7) | 7.9 (7.4-8.4) | 6.0 (5.6-6.5) | 3.4 (3.0-3.7) | 2.7 (2.4-3.0) | 4.3 (3.9-4.7) | 0.6 (0.5-0.7) |
| >0.0-959.9 mg | 20.8 (19.7-22.0) | 8.9 8.1- 9.6) | 7.5 6.8- 8.2) | 3.7 (3.2-4.2) | 2.7 (2.3-3.1) | 4.7 (4.2-5.3) | 0.5 (0.4-0.8) |
| 960-3,054.9 mg | 27.7 (26.4-29.1) | 12.1 (11.2-13.0) | 9.7 (8.9-10.5) | 4.6 (4.0-5.2) | 3.3 (2.8-3.8) | 6.3 (5.7-7.0) | 0.7 (0.5-1.0) |
| 3,055-7,299.9 mg | 32.5 (31.1-33.9) | 14.5 (13.5-15.4) | 11.5 (10.7-12.4) | 5.4 (4.8-6.0) | 3.9 (3.4-4.4) | 7.2 (6.6-7.9) | 1.2 (0.9-1.5) |
| ≥7,300 mg | 26.0 (25.0-27.0) | 11.6 (10.9-12.3) | 9.7 (9.1-10.4) | 5.1 (4.6-5.6) | 3.7 (3.4-4.2) | 5.2 (4.7-5.7) | 1.0 (0.8-1.3) |

Note: CI, confidence interval; CVD, cardiovascular disease; IQR, interquartile range; PED, prednisolone-equivalent dose.

**Table E. Cumulative incidence estimates of cardiovascular diseases per level of current daily and cumulative oral glucocorticoid prednisolone-equivalent dose by type of immune-mediated inflammatory disease**

|  | **Immune-mediated inflammatory disease** | | | | |  |
| --- | --- | --- | --- | --- | --- | --- |
| **All CVD events** | **All disease** | **Inflammatory**  **bowel disease** | **PMR and/or GCA** | **Rheumatoid arthritis** | **Systemic lupus erythematosus** | **Vasculitis** |
| Incident CVD, n (%) | 13,426 | 1,937 | 6,267 | 4,241 | 375 | 611 |
| **Cumulative probability (95% CI) at 1 year** | 2.4 (2.3-2.5) | 1.0 (0.9-1.1) | 4.4 (4.1-4.6) | 2.1 (1.9-2.3) | 1.1 (0.8-1.5) | 2.7 (2.2-3.1) |
| **Current daily PED** |  |  |  |  |  |  |
| non-use | 1.4 (1.4-1.5) | 0.8 (0.6-0.9) | 2.6 (2.3-2.9) | 1.6 (1.4-1.8) | 1.0 (0.6-1.3) | 2.1 (1.6-2.5) |
| >0.0-4.9 mg | 3.8 (3.3-4.2) | 2.4 (1.3-3.5) | 3.9 (3.4-4.5) | 3.9 (2.7-5.0) | 2.3 (0.0-4.8) | 4.1 (1.3-6.9) |
| 5.0-14.9 mg | 4.8 (4.4-5.1) | 2.3 (1.5-3.0) | 5.7 (5.2-6.3) | 4.1 (3.3-4.8) | 1.2 (0.1-2.2) | 5.0 (3.1-6.9) |
| 15.0-24.9 mg | 7.2 (6.1-8.3) | 4.1 (1.9-6.3) | 8.4 (7.0-9.8) | 6.7 (3.7-9.7) | 1.4 (0.0-4.1) | 4.9 (1.0-8.7) |
| ≥25.0 mg | 8.9 (7.4-10.4) | 4.8 (2.8-6.8) | 10.9 (8.4-13.4) | 9.4 (5.1-13.6) | 12.9 (0.2-23.9) | 13.1 (5.6-20.1) |
| **Cumulative PED** |  |  |  |  |  |  |
| non-use | 1.6 (1.4-1.7) | 0.9 (0.8-1.0) | 3.8 (3.2-4.4) | 1.7 (1.5-1.9) | 1.0 (0.7-1.4) | 2.3 (1.8-2.8) |
| >0.0-959.9 mg | 4.0 (3.6-4.3) | 1.3 (0.9-1.7) | 7.8 (6.8-8.8) | 3.4 (2.7-4.1) | 1.5 (0.2-2.9) | 4.6 (2.9-6.3) |
| 960.0-3,054.9 mg | 4.4 (4.1-4.8) | 1.2 (0.8-1.5) | 6.2 (5.7-6.7) | 3.5 (2.7-4.2) | 2.3 (0.6-4.0) | 4.6 (2.5-6.6) |
| 3,055.0-7,299.9 mg | 2.1 (1.9-2.3) | 1.0 (0.5-1.5) | 2.2 (1.9-2.5) | 3.0 (2.2-3.7) | 0.9 (0.0-1.8) | 1.8 (0.6-3.0) |
| ≥7,300.0 mg | 1.4 (1.0-1.9) | 0.5 (0.0-1.2) | 2.0 (1.1-2.8) | 1.3 (0.4-2.1) | 0.0 (0.0-0.0) | 2.5 (0.0-4.8) |
| **Cumulative probability (95% CI) at 5 years** | 10.3 (10.0-10.5) | 4.3 (4.0-4.6) | 17.4 (16.9-18.0) | 10.0 (9.6-10.4) | 5.9 (5.1-6.8) | 9.0 (8.1-9.9) |
| **Current daily PED** |  |  |  |  |  |  |
| non-use | 7.1 (6.9-7.3) | 3.5 (3.2-3.8) | 11.4 (10.8-11.9) | 8.1 (7.7-8.5) | 5.2 (4.3-6.1) | 7.5 (6.6-8.4) |
| >0.0-4.9 mg | 19.7 (18.5-20.9) | 9.3 (6.7-11.8) | 23.7 (22.1-25.3) | 15.1 (12.8-17.3) | 9.9 (4.6-14.9) | 13.7 (7.9-19.1) |
| 5.0-14.9 mg | 21.6 (20.7-22.5) | 10.6 (8.7-12.4) | 28.9 (27.4-30.3) | 18.2 (16.7-19.7) | 7.9 (4.9-10.7) | 16.5 (12.7-20.2) |
| 15.0-24.9 mg | 26.8 (24.2-29.2) | 14.0 (9.4-18.4) | 34.3 (30.5-37.9) | 23.8 (17.9-29.2) | 11.2 (2.2-19.3) | 21.1 (10.5-30.3) |
| ≥25.0 mg | 28.0 (25.1-30.7) | 15.6 (11.7-19.2) | 41.6 (35.8-46.8) | 31.2 (24.7-37.1) | 22.9 (7.3-35.6) | 19.3 (9.7-27.9) |
| **Cumulative PED** |  |  |  |  |  |  |
| non-use | 7.4 (7.1-7.7) | 4.4 (4.0-4.8) | 15.2 (13.8-16.6) | 8.6 (8.1-9.1) | 5.6 (4.5-6.7) | 8.2 (7.1-9.3) |
| >0.0-959.9 mg | 9.7 (9.1-10.3) | 4.1 (3.5-4.8) | 19.5 (17.8-21.1) | 9.3 (8.2-10.3) | 7.2 (4.6-9.6) | 9.5 (7.2-11.8) |
| 960.0-3,054.9 mg | 13.9 (13.3-14.6) | 4.3 (3.6-4.9) | 22.0 (20.8-23.1) | 14.1 (12.4-15.7) | 6.6 (3.5-9.6) | 11.8 (8.0-15.4) |
| 3,055.0-7,299.9 mg | 15.4 (14.7-16.1) | 4.0 (3.1-4.8) | 19.0 (18.0-19.9) | 18.2 (16.4-20.0) | 9.4 (5.3-13.2) | 12.4 (8.6-16.0) |
| ≥7,300.0 mg | 9.7 (9.1-10.3) | 4.0 (2.9-5.0) | 11.9 (11.1-12.8) | 9.1 (7.9-10.2) | 3.9 (2.0-5.9) | 8.3 (5.8-10.7) |
| **Cumulative probability (95% CI) at 10 years** | 19.1 (18.7-19.4) | 8.7 (8.2-9.1) | 31.2 (30.5-32.0) | 19.9 (19.2-20.5) | 11.5 (10.1-12.8) | 15.7 (14.3-17.0) |
| **Current daily PED** |  |  |  |  |  |  |
| non-use | 14.6 (14.2-14.9) | 7.4 (7.0-7.8) | 24.0 (23.1-24.8) | 16.4 (15.7-17.0) | 9.8 (8.5-11.2) | 13.4 (12.0-14.8) |
| >0.0-4.9 mg | 33.0 (31.4-34.6) | 17.3 (13.3-21.0) | 39.5 (37.2-41.6) | 27.7 (24.6-30.7) | 22.6 (13.2-30.9) | 25.3 (16.5-33.1) |
| 5.0-14.9 mg | 35.1 (33.9-36.2) | 18.4 (15.7-21.0) | 46.9 (44.8-48.8) | 31.9 (30.0-33.8) | 14.6 (10.4-18.6) | 26.0 (21.0-30.7) |
| 15.0-24.9 mg | 42.5 (38.8-45.9) | 26.0 (17.7-33.3) | 50.6 (45.3-55.3) | 40.8 (32.7-47.6) | 30.3 (7.2-46.7) | 30.8 (15.0-43.1) |
| ≥25.0 mg | 39.9 (36.3-43.1) | 25.8 (20.5-30.8) | 57.6 (50.5-63.3) | 42.0 (35.0-48.1) | 29.4 (11.1-43.3) | 29.2 (11.4-42.7) |
| **Cumulative PED** |  |  |  |  |  |  |
| non-use | 14.5 (14.0-14.9) | 9.1 (8.4-9.8) | 26.4 (24.2-28.4) | 17.5 (16.7-18.3) | 10.8 (9.0-12.6) | 13.6 (11.9-15.4) |
| >0.0-959.9 mg | 17.6 (16.7-18.5) | 8.4 (7.3-9.5) | 33.2 (30.8-35.5) | 18.6 (17.1-20.2) | 12.4 (8.8-15.8) | 17.9 (14.2-21.4) |
| 960.0-3,054.9 mg | 20.8 (20.0-21.7) | 7.7 (6.7-8.7) | 34.1 (32.4-35.8) | 21.7 (19.6-23.7) | 9.9 (5.7-13.8) | 13.6 (9.3-17.6) |
| 3,055.0-7,299.9 mg | 24.7 (23.8-25.6) | 8.2 (7.0-9.4) | 32.4 (30.9-33.8) | 28.2 (25.9-30.5) | 12.8 (7.7-17.6) | 19.7 (14.3-24.6) |
| ≥7,300.0 mg | 21.9 (21.1-22.7) | 8.7 (7.3-10.0) | 28.2 (26.8-29.5) | 21.9 (20.2-23.4) | 11.7 (8.4-14.9) | 18.4 (14.7-22.0) |
| **Atrial fibrillation events** | **All disease** | **Inflammatory**  **bowel disease** | **PMR and/or GCA** | **Rheumatoid arthritis** | **Systemic lupus erythematosus** | **Vasculitis** |
| Incident CVD, n (%) | 6,013 | 887 | 3,024 | 1,719 | 131 | 254 |
| **Cumulative probability (95% CI) at 1 year** | 1.0 (0.9-1.0) | 0.5 (0.4-0.5) | 1.9 (1.7-2.1) | 0.6 (0.5-0.7) | 0.3 (0.1-0.5) | 1.0 (0.7-1.3) |
| **Current daily PED** |  |  |  |  |  |  |
| non-use | 0.5 (0.5-0.6) | 0.3 (0.3-0.4) | 0.9 (0.7-1.1) | 0.5 (0.4-0.6) | 0.3 (0.1-0.5) | 0.8 (0.6-1.1) |
| >0.0-4.9 mg | 1.5 (1.2-1.8) | 0.9 (0.2-1.5) | 1.7 (1.4-2.1) | 1.0 (0.4-1.6) | 1.5 (0.0-3.6) | 1.5 (0.0-3.2) |
| 5.0-14.9 mg | 2.0 (1.8-2.3) | 1.0 (0.5-1.5) | 2.8 (2.4-3.1) | 1.1 (0.7-1.4) | 0.2 (0.0-0.7) | 1.4 (0.4-2.4) |
| 15.0-24.9 mg | 3.3 (2.6-4.1) | 1.6 (0.2-3.1) | 3.9 (2.9-5.0) | 3.1 (0.9-5.1) | 0.0 (0.0-0.0) | 3.3 (0.1-6.4) |
| ≥25.0 mg | 3.9 (2.8-4.9) | 3.9 (2.1-5.7) | 4.9 (3.1-6.6) | 1.9 (0.0-3.9) | 0.0 (0.0-0.0) | 2.7 (0.0-6.3) |
| **Cumulative PED** |  |  |  |  |  |  |
| non-use | 0.6 (0.5-0.7) | 0.4 (0.3-0.5) | 1.6 (1.2-2.0) | 0.5 (0.4-0.6) | 0.3 (0.1-0.5) | 1.0 (0.6-1.3) |
| >0.0-959.9 mg | 1.4 (1.2-1.6) | 0.8 (0.5-1.1) | 2.6 (2.1-3.2) | 1.0 (0.6-1.3) | 0.3 (0.0-0.9) | 1.3 (0.4-2.3) |
| 960.0-3,054.9 mg | 2.0 (1.7-2.2) | 0.6 (0.3-0.8) | 2.9 (2.5-3.2) | 1.1 (0.6-1.5) | 1.0 (0.0-2.1) | 1.5 (0.3-2.7) |
| 3,055.0-7,299.9 mg | 0.9 (0.7-1.1) | 0.2 (0.0-0.4) | 1.1 (0.8-1.3) | 0.9 (0.5-1.4) | 0.0 (0.0-0.0) | 0.6 (0.0-1.3) |
| ≥7,300.0 mg | 0.3 (0.1-0.5) | 0.0 (0.0-0.0) | 0.4 (0.0-0.8) | 0.3 (0.0-0.6) | 0.0 (0.0-0.0) | 0.6 (0.0-1.8) |
| **Cumulative probability (95% CI) at 5 years** | 4.1 (4.0-4.3) | 1.8 (1.6-1.9) | 7.6 (7.3-8.0) | 3.5 (3.3-3.8) | 1.4 (1.0-1.8) | 3.7 (3.1-4.2) |
| **Current daily PED** |  |  |  |  |  |  |
| non-use | 2.7 (2.6-2.9) | 1.4 (1.3-1.6) | 4.6 (4.3-5.0) | 2.8 (2.5-3.0) | 1.4 (1.0-1.9) | 3.2 (2.6-3.8) |
| >0.0-4.9 mg | 8.7 (7.9-9.6) | 3.4 (1.8-5.1) | 11.2 (10.0-12.4) | 5.6 (4.1-7.0) | 3.0 (0.0-5.9) | 5.8 (2.0-9.5) |
| 5.0-14.9 mg | 9.2 (8.6-9.9) | 4.2 (2.9-5.4) | 13.6 (12.5-14.8) | 7.0 (6.0-8.0) | 0.9 (0.0-1.8) | 4.9 (2.7-7.1) |
| 15.0-24.9 mg | 12.5 (10.5-14.4) | 4.3 (1.5-6.9) | 17.9 (14.7-21.1) | 10.2 (5.9-14.3) | 2.3 (0.0-6.6) | 8.2 (1.4-14.5) |
| ≥25.0 mg | 13.0 (10.7-15.2) | 9.3 (6.3-12.3) | 21.6 (16.4-26.5) | 9.8 (5.2-14.2) | 3.5 (0.0-9.9) | 8.0 (0.8-14.6) |
| **Cumulative PED** |  |  |  |  |  |  |
| non-use | 2.8 (2.6-2.9) | 1.8 (1.5-2.0) | 6.9 (5.9-7.9) | 2.8 (2.5-3.1) | 1.5 (0.9-2.0) | 3.9 (3.1-4.6) |
| >0.0-959.9 mg | 3.9 (3.5-4.3) | 2.1 (1.6-2.5) | 8.2 (7.0-9.3) | 3.4 (2.7-4.0) | 2.4 (0.9-3.8) | 3.6 (2.1-5.1) |
| 960.0-3,054.9 mg | 6.0 (5.5-6.4) | 1.9 (1.4-2.3) | 10.0 (9.1-10.9) | 5.3 (4.2-6.4) | 1.7 (0.2-3.2) | 3.3 (1.2-5.4) |
| 3,055.0-7,299.9 mg | 6.6 (6.1-7.0) | 1.4 (0.9-1.9) | 8.6 (7.9-9.4) | 6.8 (5.6-8.1) | 0.6 (0.0-1.6) | 4.9 (2.4-7.4) |
| ≥7,300.0 mg | 3.7 (3.3-4.1) | 1.2 (0.6-1.8) | 4.7 (4.2-5.3) | 3.6 (2.9-4.3) | 0.5 (0.0-1.2) | 2.0 (0.8-3.2) |
| **Cumulative probability (95% CI) at 10 years** | 8.7 (8.4-8.9) | 3.8 (3.5-4.1) | 15.7 (15.1-16.3) | 8.2 (7.7-8.6) | 4.0 (3.1-4.8) | 6.7 (5.7-7.7) |
| **Current daily PED** |  |  |  |  |  |  |
| non-use | 6.5 (6.2-6.7) | 3.2 (2.9-3.5) | 11.8 (11.1-12.5) | 6.6 (6.1-7.1) | 3.5 (2.6-4.3) | 6.0 (5.0-7.0) |
| >0.0-4.9 mg | 16.6 (15.3-17.9) | 6.9 (4.2-9.5) | 20.9 (18.9-22.9) | 13.0 (10.4-15.4) | 8.7 (2.2-14.7) | 11.6 (5.1-17.6) |
| 5.0-14.9 mg | 16.4 (15.4-17.3) | 8.3 (6.4-10.3) | 24.5 (22.5-26.4) | 13.5 (12.0-14.9) | 4.2 (1.7-6.7) | 9.9 (6.2-13.5) |
| 15.0-24.9 mg | 19.9 (16.8-22.9) | 9.7 (4.0-15.1) | 26.4 (21.3-31.1) | 17.7 (11.4-23.5) | 12.0 (0.0-24.4) | 8.1 (1.4-14.3) |
| ≥25.0 mg | 22.1 (18.8-25.3) | 16.1 (11.5-20.4) | 39.6 (31.3-46.7) | 16.4 (10.5-21.8) | 9.4 (0.0-19.1) | 10.1 (1.2-18.0) |
| **Cumulative PED** |  |  |  |  |  |  |
| non-use | 6.3 (5.9-6.6) | 4.0 (3.5-4.5) | 14.3 (12.4-16.0) | 7.1 (6.5-7.7) | 3.7 (2.6-4.8) | 6.7 (5.48.0) |
| >0.0-959.9 mg | 7.9 (7.3-8.5) | 3.8 (3.1-4.5) | 16.4 (14.4-18.4) | 8.1 (7.0-9.2) | 5.1 (2.7-7.4) | 6.5 (4.1- 8.8) |
| 960.0-3,054.9 mg | 9.9 (9.2-10.6) | 3.5 (2.8-4.2) | 17.6 (16.1-19.1) | 9.1 (7.6-10.6) | 3.7 (0.8-6.5) | 5.4 (2.3-8.4) |
| 3,055.0-7,299.9 mg | 11.8 (11.1-12.6) | 3.4 (2.6-4.2) | 16.9 (15.7-18.1) | 11.3 (9.5-13.0) | 2.0 (0.0-4.2) | 8.7 (4.7-12.5) |
| ≥7,300.0 mg | 9.8 (9.2-10.4) | 3.7 (2.8-4.6) | 13.5 (12.4-14.5) | 8.9 (7.8-10.0) | 4.4 (2.2-6.5) | 5.9 (3.6-8.1) |
| **Heart failure events** | **All disease** | **Inflammatory**  **bowel disease** | **PMR and/or GCA** | **Rheumatoid arthritis** | **Systemic lupus erythematosus** | **Vasculitis** |
| Incident CVD, n (%) | 4,727 | 535 | 2,319 | 1,537 | 138 | 201 |
| **Cumulative probability (95% CI) at 1 year** | 0.7 (0.6-0.7) | 0.2 (0.2-0.3) | 1.3 (1.2-1.4) | 0.6 (0.5-0.7) | 0.4 (0.2-0.5) | 0.7 (0.5-1.0) |
| **Current daily PED** |  |  |  |  |  |  |
| non-use | 0.4 (0.3-0.4) | 0.2 (0.1-0.2) | 0.8 (0.6-1.0) | 0.4 (0.3-0.5) | 0.3 (0.1- 0.5) | 0.5 (0.3-0.7) |
| >0.0-4.9 mg | 1.0 (0.8-1.2) | 0.3 (0.0-0.7) | 1.0 (0.8-1.3) | 1.1 (0.4-1.7) | 0.0 (0.0- 0.0) | 2.1 (0.0-4.1) |
| 5.0-14.9 mg | 1.5 (1.3-1.7) | 1.0 (0.5-1.5) | 1.8 (1.5-2.1) | 1.4 (1.0-1.8) | 0.2 (0.0- 0.7) | 1.0 (0.1-1.8) |
| 15.0-24.9 mg | 2.0 (1.4-2.6) | 1.0 (0.0-2.2) | 2.3 (1.5-3.1) | 3.0 (0.9-5.1) | 0.0 (0.0- 0.0) | 0.8 (0.0-2.5) |
| ≥25.0 mg | 3.1 (2.2-4.1) | 0.7 (0.0-1.4) | 3.4 (1.9-4.9) | 5.6 (2.1-8.8) | 6.6 (0.0-15.0) | 7.3 (1.5-12.8) |
| **Cumulative PED** |  |  |  |  |  |  |
| non-use | 0.4 (0.3-0.5) | 0.2 (0.1-0.2) | 1.1 (0.7-1.4) | 0.4 (0.3-0.5) | 0.4 (0.2-0.6) | 0.6 (0.3-0.8) |
| >0.0-959.9 mg | 1.2 (1.0-1.4) | 0.3 (0.1-0.5) | 2.4 (1.9-3.0) | 1.1 (0.7-1.5) | 0.0 (0.0-0.0) | 1.5 (0.5-2.4) |
| 960.0-3,054.9 mg | 1.2 (1.0-1.4) | 0.5 (0.2-0.7) | 1.6 (1.4-1.9) | 1.1 (0.7-1.5) | 0.7 (0.0-1.6) | 0.8 (0.0-1.7) |
| 3,055.0-7,299.9 mg | 0.7 (0.6-0.9) | 0.1 (0.0-0.3) | 0.8 (0.6-0.9) | 1.0 (0.6-1.5) | 0.3 (0.0-0.8) | 0.6 (0.0-1.2) |
| ≥7,300.0 mg | 0.6 (0.3-0.9) | 0.0 (0.0-0.0) | 0.9 (0.3-1.5) | 0.4 (0.0-0.8) | 0.0 (0.0-0.0) | 1.2 (0.0-2.9) |
| **Cumulative probability (95% CI) at 5 years** | 3.2 (3.1-3.4) | 1.0 (0.9-1.2) | 5.9 (5.5-6.2) | 3.2 (2.9-3.4) | 1.9 (1.4-2.4) | 2.5 (2.0-2.9) |
| **Current daily PED** |  |  |  |  |  |  |
| non-use | 2.1 (1.9-2.2) | 0.7 (0.6-0.8) | 3.8 (3.5-4.1) | 2.3 (2.0-2.5) | 1.5 (1.0-2.0) | 1.9 (1.4-2.3) |
| >0.0-4.9 mg | 6.7 (6.0-7.5) | 2.9 (1.4-4.4) | 8.5 (7.4-9.5) | 4.6 (3.2-5.9) | 1.1 (0.0-3.2) | 6.0 (2.0-9.9) |
| 5.0-14.9 mg | 7.8 (7.2-8.4) | 4.0 (2.8-5.2) | 10.4 (9.4-11.4) | 7.0 (6.0-8.0) | 2.6 (0.8-4.3) | 4.0 (2.0-5.9) |
| 15.0-24.9 mg | 10.2 (8.4-12.0) | 3.8 (1.1-6.4) | 12.2 (9.4-14.8) | 12.7 (8.0-17.2) | 9.7 (1.2-17.4) | 6.7 (0.0-13.0) |
| ≥25.0 mg | 11.6 (9.5-13.7) | 7.0 (3.9-10.0) | 13.8 (9.5-17.8) | 14.5 (9.5-19.3) | 20.2 (5.4-32.6) | 12.4 (4.1-19.8) |
| **Cumulative PED** |  |  |  |  |  |  |
| non-use | 2.0 (1.8-2.1) | 0.9 (0.7-1.1) | 4.8 (3.9-5.6) | 2.5 (2.2-2.7) | 1.7 (1.1-2.3) | 1.9 (1.3-2.4) |
| >0.0-959.9 mg | 3.0 (2.7-3.3) | 1.0 (0.6-1.3) | 6.5 (5.5-7.5) | 3.0 (2.4-3.6) | 2.3 (0.8-3.8) | 3.2 (1.8-4.5) |
| 960.0-3,054.9 mg | 4.3 (3.9-4.7) | 1.1 (0.8-1.5) | 7.2 (6.4-8.0) | 4.5 (3.5-5.5) | 1.5 (0.0-3.0) | 3.5 (1.3-5.7) |
| 3,055.0-7,299.9 mg | 5.3 (4.9-5.8) | 1.4 (0.9-1.9) | 6.6 (6.0-7.2) | 6.3 (5.2-7.5) | 2.8 (0.5-5.0) | 4.1 (1.8-6.3) |
| ≥7,300.0 mg | 3.5 (3.2-3.9) | 1.5 (0.8-2.1) | 4.2 (3.7-4.7) | 3.6 (2.9-4.3) | 2.1 (0.6-3.5) | 2.6 (1.2-4.0) |
| **Cumulative probability (95% CI) at 10 years** | 6.8 (6.6-7.0) | 2.3 (2.1-2.6) | 12.1 (11.6-12.7) | 7.0 (6.6-7.5) | 4.4 (3.5-5.3) | 5.6 (4.6-6.5) |
| **Current daily PED** |  |  |  |  |  |  |
| non-use | 4.7 (4.5-5.0) | 1.8 (1.5-2.0) | 8.7 (8.2-9.3) | 5.3 (4.9-5.7) | 3.7 (2.8-4.7) | 4.1 (3.2-4.9) |
| >0.0-4.9 mg | 13.1 (11.9-14.4) | 6.5 (3.8-9.1) | 17.1 (15.2-19.0) | 9.3 (7.2-11.3) | 2.2 (0.0-5.2) | 13.8 (6.6-20.4) |
| 5.0-14.9 mg | 14.7 (13.7-15.6) | 6.3 (4.6-7.9) | 20.5 (18.7-22.3) | 13.8 (12.4-15.3) | 5.9 (2.9-8.8) | 9.5 (5.6-13.2) |
| 15.0-24.9 mg | 20.9 (17.7-24.0) | 9.2 (3.6-14.3) | 25.2 (20.1-29.9) | 21.4 (14.5-27.6) | 15.4 (1.7-26.9) | 24.5 (7.2-38.0) |
| ≥25.0 mg | 19.5 (16.3-22.6) | 11.6 (7.5-15.5) | 28.0 (20.1-34.9) | 20.4 (14.2-26.1) | 26.0 (7.8-40.1) | 25.2 (5.7-40.0) |
| **Cumulative PED** |  |  |  |  |  |  |
| non-use | 4.5 (4.2-4.8) | 2.4 (2.0-2.7) | 9.3 (7.8-10.7) | 5.6 (5.1-6.1) | 4.0 (2.8-5.2) | 4.4 (3.2-5.6) |
| >0.0-959.9 mg | 6.2 (5.6-6.8) | 2.2 (1.6-2.7) | 13.4 (11.5-15.2) | 6.5 (5.5-7.5) | 6.3 (3.4-9.1) | 5.4 (3.3-7.5) |
| 960.0-3,054.9 mg | 7.6 (7.0-8.2) | 1.9 (1.4-2.3) | 14.0 (12.6-15.4) | 8.0 (6.6-9.4) | 2.3 (0.4-4.2) | 4.7 (2.0-7.2) |
| 3,055.0-7,299.9 mg | 9.3 (8.6-9.9) | 2.5 (1.8-3.2) | 12.1 (11.1-13.1) | 11.3 (9.5-13.0) | 6.6 (2.4-10.6) | 9.2 (4.9-13.2) |
| ≥7,300.0 mg | 8.6 (8.0-9.2) | 2.9 (2.1-3.6) | 11.3 (10.4-12.2) | 8.4 (7.3-9.5) | 4.0 (2.0-6.0) | 7.6 (5.0-10.2) |
| **Acute myocardial infarction events** | **All disease** | **Inflammatory**  **bowel disease** | **PMR and/or GCA** | **Rheumatoid arthritis** | **Systemic lupus erythematosus** | **Vasculitis** |
| Incident CVD, n (%) | 2,809 | 380 | 1,223 | 1,013 | 86 | 108 |
| **Cumulative probability (95% CI) at 1 year** | 0.4 (0.3-0.4) | 0.1 (0.1-0.2) | 0.6 (0.5-0.7) | 0.4 (0.3-0.4) | 0.2 (0.1-0.4) | 0.4 (0.2-0.5) |
| **Current daily PED** |  |  |  |  |  |  |
| non-use | 0.2 (0.2-0.3) | 0.1 (0.1-0.1) | 0.4 (0.3-0.5) | 0.3 (0.2-0.4) | 0.2 (0.0-0.3) | 0.2 (0.1-0.4) |
| >0.0-4.9 mg | 0.4 (0.3-0.6) | 0.0 (0.0-0.0) | 0.5 (0.3-0.7) | 0.6 (0.1-1.1) | 0.7 (0.0-2.2) | 0.0 (0.0-0.0) |
| 5.0-14.9 mg | 0.8 (0.6-0.9) | 0.4 (0.1-0.7) | 0.8 (0.6-1.0) | 0.9 (0.5-1.2) | 0.0 (0.0-0.0) | 1.0 (0.1-1.8) |
| 15.0-24.9 mg | 0.7 (0.3-1.0) | 0.7 (0.0-1.6) | 0.9 (0.4-1.4) | 0.0 (0.0-0.0) | 0.0 (0.0-0.0) | 0.0 (0.0-0.0) |
| ≥25.0 mg | 1.7 (1.0-2.4) | 0.7 (0.0-1.5) | 1.7 (0.7-2.8) | 1.7 (0.0-3.6) | 7.0 (0.0-15.8) | 5.0 (0.1-9.7) |
| **Cumulative PED** |  |  |  |  |  |  |
| non-use | 0.3 (0.2-0.3) | 0.1 (0.1-0.2) | 0.6 (0.4-0.9) | 0.3 (0.2-0.4) | 0.2 (0.0-0.4) | 0.2 (0.1-0.4) |
| >0.0-959.9 mg | 0.7 (0.5-0.8) | 0.1 (0.0-0.2) | 1.3 (0.9-1.7) | 0.8 (0.4-1.1) | 0.6 (0.0-1.5) | 1.0 (0.2-1.8) |
| 960.0-3,054.9 mg | 0.6 (0.4-0.7) | 0.1 (0.0-0.3) | 0.8 (0.6-1.0) | 0.5 (0.2-0.8) | 0.3 (0.0-0.9) | 0.8 (0.0-1.7) |
| 3,055.0-7,299.9 mg | 0.3 (0.2-0.4) | 0.3 (0.0-0.5) | 0.3 (0.2-0.4) | 0.5 (0.2-0.8) | 0.0 (0.0-0.0) | 0.4 (0.0-0.9) |
| ≥7,300.0 mg | 0.1 (0.0-0.3) | 0.0 (0.0-0.0) | 0.3 (0.0-0.6) | 0.0 (0.0-0.0) | 0.0 (0.0-0.0) | 0.0 (0.0-0.0) |
| **Cumulative probability (95% CI) at 5 years** | 2.1 (2.0-2.2) | 0.8 (0.7-0.9) | 3.4 (3.2-3.7) | 2.3 (2.1-2.5) | 1.3 (0.9-1.7) | 1.5 (1.1-1.9) |
| **Current daily PED** |  |  |  |  |  |  |
| non-use | 1.4 (1.3-1.5) | 0.7 (0.6-0.8) | 2.2 (2.0-2.5) | 1.8 (1.6-2.0) | 1.3 (0.8-1.7) | 0.9 (0.6-1.3) |
| >0.0-4.9 mg | 3.7 (3.2-4.3) | 1.2 (0.2-2.2) | 4.6 (3.8-5.5) | 2.7 (1.7-3.8) | 2.9 (0.0-5.6) | 3.5 (0.4-6.5) |
| 5.0-14.9 mg | 4.8 (4.3-5.3) | 2.3 (1.4-3.2) | 6.3 (5.5-7.2) | 4.6 (3.8-5.5) | 1.0 (0.0-2.1) | 3.6 (1.7-5.5) |
| 15.0-24.9 mg | 4.8 (3.4-6.2) | 1.7 (0.0-3.4) | 7.0 (4.6-9.3) | 3.1 (0.6-5.5) | 0.0 (0.0-0.0) | 7.2 (0.0-13.9) |
| ≥25.0 mg | 6.8 (5.1-8.5) | 2.9 (1.2-4.6) | 11.4 (7.3-15.3) | 7.5 (3.4-11.4) | 5.2 (0.0-11.9) | 6.6 (0.6-12.3) |
| **Cumulative PED** |  |  |  |  |  |  |
| non-use | 1.5 (1.3-1.6) | 0.7 (0.5-0.9) | 2.9 (2.2-3.5) | 2.1 (1.8-2.3) | 1.5 (1.0-2.1) | 0.7 (0.4-1.1) |
| >0.0-959.9 mg | 1.8 (1.6-2.1) | 1.0 (0.7-1.4) | 3.0 (2.3-3.8) | 1.8 (1.3-2.3) | 1.4 (0.3-2.5) | 2.5 (1.2-3.7) |
| 960.0-3,054.9 mg | 2.6 (2.2-2.9) | 0.8 (0.5-1.1) | 4.1 (3.5-4.7) | 2.6 (1.8-3.4) | 1.1 (0.0-2.3) | 2.8 (0.8-4.8) |
| 3,055.0-7,299.9 mg | 3.3 (2.9-3.6) | 0.9 (0.5-1.3) | 3.8 (3.3-4.3) | 4.7 (3.6-5.7) | 1.6 (0.0-3.5) | 2.9 (1.1-4.8) |
| ≥7,300.0 mg | 2.2 (1.9-2.5) | 1.1 (0.5-1.6) | 2.8 (2.3-3.2) | 2.0 (1.4-2.5) | 0.3 (0.0-0.8) | 1.9 (0.7-3.1) |
| **Cumulative probability (95% CI) at 10 years** | 4.2 (4.0-4.3) | 1.8 (1.6-2.0) | 6.5 (6.1-7.0) | 4.8 (4.5-5.2) | 2.7 (2.0-3.4) | 3.0 (2.4-3.7) |
| **Current daily PED** |  |  |  |  |  |  |
| non-use | 3.1 (2.9-3.2) | 1.4 (1.2-1.6) | 4.6 (4.2-5.1) | 3.9 (3.5-4.2) | 2.7 (2.0-3.5) | 2.3 (1.7-3.0) |
| >0.0-4.9 mg | 7.2 (6.3-8.2) | 4.2 (1.9-6.3) | 8.8 (7.4-10.2) | 5.9 (4.3-7.6) | 5.9 (0.6-10.8) | 3.3 (0.4-6.2) |
| 5.0-14.9 mg | 8.9 (8.2-9.7) | 4.7 (3.2-6.3) | 11.7 (10.2-13.1) | 9.0 (7.8-10.2) | 1.3 (0.0-2.5) | 6.9 (3.9-9.7) |
| 15.0-24.9 mg | 10.6 (7.8-13.2) | 6.5 (0.7-11.9) | 13.3 (8.9-17.4) | 8.5 (3.5-13.2) | 4.6 (0.0-12.9) | 11.7 (0.0-22.2) |
| ≥25.0 mg | 10.5 (7.9-12.9) | 7.0 (3.5-10.4) | 15.7 (9.7-21.2) | 11.7 (6.3-16.8) | 3.6 (0.0-8.4) | 5.8 (0.6-10.7) |
| **Cumulative PED** |  |  |  |  |  |  |
| non-use | 3.1 (2.8-3.3) | 1.6 (1.3-1.9) | 5.4 (4.3-6.6) | 4.1 (3.6-4.5) | 3.7 (2.6-4.9) | 1.9 (1.1-2.7) |
| >0.0-959.9 mg | 3.6 (3.2-4.0) | 1.7 (1.2-2.2) | 6.1 (4.8-7.3) | 4.3 (3.5-5.2) | 1.4 (0.4-2.5) | 4.9 (2.8-6.9) |
| 960.0-3,054.9 mg | 4.4 (3.9-4.8) | 1.6 (1.1-2.1) | 7.4 (6.3-8.4) | 4.6 (3.5-5.7) | 2.9 (0.3-5.5) | 3.0 (0.9-4.9) |
| 3,055.0-7,299.9 mg | 5.2 (4.7-5.7) | 2.0 (1.4-2.6) | 6.2 (5.4-6.9) | 7.9 (6.4-9.3) | 1.4 (0.0-3.0) | 3.4 (1.3-5.5) |
| ≥7,300.0 mg | 5.2 (4.8-5.7) | 2.4 (1.7-3.1) | 6.5 (5.7-7.2) | 5.6 (4.7-6.5) | 1.3 (0.2-2.5) | 4.2 (2.3-6.1) |
| **Peripheral arterial disease events** | **All disease** | **Inflammatory**  **bowel disease** | **PMR and/or GCA** | **Rheumatoid arthritis** | **Systemic lupus erythematosus** | **Vasculitis** |
| Incident CVD, n (%) | 1,972 | 298 | 851 | 629 | 71 | 126 |
| **Cumulative probability (95% CI) at 1 year** | 0.3 (0.3-0.4) | 0.1 (0.1-0.2) | 0.6 (0.5-0.7) | 0.3 (0.2-0.3) | 0.2 (0.0-0.3) | 0.5 (0.3-0.7) |
| **Current daily PED** |  |  |  |  |  |  |
| non-use | 0.2 (0.2-0.2) | 0.1 (0.1-0.1) | 0.3 (0.2-0.4) | 0.2 (0.2-0.3) | 0.2 (0.0-0.3) | 0.5 (0.3-0.7) |
| >0.0-4.9 mg | 0.4 (0.3-0.6) | 0.4 (0.0-0.9) | 0.5 (0.3-0.7) | 0.3 (0.0-0.6) | 0.0 (0.0-0.0) | 0.5 (0.0-1.6) |
| 5.0-14.9 mg | 0.7 (0.6-0.8) | 0.3 (0.0-0.6) | 0.8 (0.6-1.0) | 0.5 (0.3-0.8) | 0.2 (0.0-0.7) | 0.9 (0.1-1.8) |
| 15.0-24.9 mg | 1.0 (0.6-1.5) | 0.9 (0.0-2.0) | 1.1 (0.5-1.6) | 0.8 (0.0-1.9) | 1.4 (0.0-4.1) | 0.8 (0.0-2.4) |
| ≥25.0 mg | 0.8 (0.3-1.3) | 0.0 (0.0-0.0) | 1.2 (0.3-2.2) | 1.2 (0.0-2.9) | 0.0 (0.0-0.0) | 1.1 (0.0-3.2) |
| **Cumulative PED** |  |  |  |  |  |  |
| non-use | 0.2 (0.2-0.3) | 0.1 (0.1-0.2) | 0.4 (0.2-0.6) | 0.2 (0.2-0.3) | 0.2 (0.0-0.3) | 0.6 (0.3-0.8) |
| >0.0-959.9 mg | 0.5 (0.3-0.6) | 0.2 (0.0-0.3) | 0.9 (0.5-1.2) | 0.3 (0.1-0.5) | 0.3 (0.0-0.9) | 1.1 (0.3-2.0) |
| 960.0-3,054.9 mg | 0.6 (0.5-0.7) | 0.1 (0.0-0.2) | 0.9 (0.7-1.1) | 0.4 (0.2-0.7) | 0.0 (0.0-0.0) | 0.5 (0.0-1.1) |
| 3,055.0-7,299.9 mg | 0.3 (0.2-0.4) | 0.2 (0.0-0.4) | 0.3 (0.2-0.4) | 0.4 (0.1-0.7) | 0.3 (0.0-0.9) | 0.0 (0.0-0.0) |
| ≥7,300.0 mg | 0.2 (0.0-0.4) | 0.5 (0.0-1.2) | 0.1 (0.0-0.3) | 0.4 (0.0-0.8) | 0.0 (0.0-0.0) | 0.0 (0.0-0.0) |
| **Cumulative probability (95% CI) at 5 years** | 1.5 (1.4-1.6) | 0.6 (0.5-0.7) | 2.4 (2.2-2.6) | 1.5 (1.4-1.7) | 1.1 (0.7-1.5) | 1.9 (1.5-2.3) |
| **Current daily PED** |  |  |  |  |  |  |
| non-use | 1.1 (1.0-1.2) | 0.5 (0.4-0.6) | 1.6 (1.3-1.8) | 1.3 (1.1-1.4) | 1.0 (0.6-1.4) | 1.5 (1.1-1.9) |
| >0.0-4.9 mg | 2.5 (2.0-2.9) | 1.1 (0.2-2.0) | 2.9 (2.2-3.5) | 2.2 (1.2-3.1) | 1.5 (0.0-3.6) | 2.2 (0.0-4.7) |
| 5.0-14.9 mg | 3.3 (2.9-3.7) | 1.8 (1.0-2.6) | 4.5 (3.7-5.2) | 2.6 (1.9-3.2) | 1.8 (0.3-3.2) | 4.6 (2.5-6.7) |
| 15.0-24.9 mg | 4.3 (3.1-5.5) | 2.9 (0.7-5.1) | 5.2 (3.2-7.1) | 3.0 (0.7-5.2) | 3.3 (0.0-7.8) | 6.1 (0.0-11.8) |
| ≥25.0 mg | 3.7 (2.4-5.1) | 0.3 (0.0-0.9) | 6.4 (3.3-9.3) | 7.3 (3.2-11.2) | 0.0 (0.0-0.0) | 1.1 (0.0-3.1) |
| **Cumulative PED** |  |  |  |  |  |  |
| non-use | 1.2 (1.0-1.3) | 0.8 (0.6-0.9) | 2.3 (1.7-2.9) | 1.3 (1.1-1.5) | 1.0 (0.5-1.4) | 1.6 (1.1-2.1) |
| >0.0-959.9 mg | 1.4 (1.1-1.6) | 0.4 (0.2-0.6) | 2.6 (2.0-3.3) | 1.3 (0.9-1.7) | 1.7 (0.4-3.0) | 2.6 (1.3-3.9) |
| 960.0-3,054.9 mg | 2.0 (1.7-2.2) | 0.4 (0.2-0.7) | 3.1 (2.6-3.7) | 2.6 (1.8-3.3) | 0.4 (0.0-1.2) | 0.9 (0.0-1.9) |
| 3,055.0-7,299.9 mg | 2.1 (1.8-2.4) | 0.6 (0.3-0.9) | 2.4 (2.0-2.8) | 3.0 (2.1-3.8) | 2.7 (0.5-4.9) | 2.4 (0.6-4.2) |
| ≥7,300.0 mg | 1.4 (1.2-1.7) | 0.7 (0.3-1.1) | 1.6 (1.3-2.0) | 1.3 (0.9-1.8) | 0.8 (0.0-1.6) | 2.1 (0.9-3.4) |
| **Cumulative probability (95% CI) at 10 years** | 2.9 (2.8-3.1) | 1.4 (1.2-1.6) | 4.6 (4.3-5.0) | 3.1 (2.8-3.3) | 2.3 (1.7-3.0) | 3.5 (2.7-4.2) |
| **Current daily PED** |  |  |  |  |  |  |
| non-use | 2.2 (2.0-2.3) | 1.2 (1.0-1.3) | 3.2 (2.9-3.6) | 2.5 (2.2-2.8) | 1.8 (1.2-2.4) | 2.8 (2.1-3.5) |
| >0.0-4.9 mg | 5.9 (5.0-6.8) | 4.2 (2.0-6.4) | 6.4 (5.1-7.7) | 5.3 (3.7-7.0) | 8.9 (2.5-14.9) | 3.4 (0.0-7.0) |
| 5.0-14.9 mg | 6.0 (5.4-6.6) | 2.5 (1.5-3.5) | 8.8 (7.4-10.1) | 4.9 (3.9-5.8) | 2.3 (0.6-4.0) | 8.2 (4.8-11.5) |
| 15.0-24.9 mg | 6.9 (4.9-8.9) | 7.0 (1.7-11.9) | 7.7 (4.6-10.7) | 3.3 (0.9-5.6) | 12.7 (0.0-24.7) | 9.5 (0.0-18.1) |
| ≥25.0 mg | 6.4 (4.4-8.3) | 3.6 (0.9-6.2) | 9.4 (4.8-13.7) | 8.7 (4.4-12.8) | 3.0 (0.0-8.6) | 0.9 (0.0-2.6) |
| **Cumulative PED** |  |  |  |  |  |  |
| non-use | 2.3 (2.0-2.5) | 1.4 (1.1-1.7) | 3.8 (2.8-4.7) | 2.7 (2.3-3.0) | 2.1 (1.2-2.9) | 2.7 (1.9-3.5) |
| >0.0-959.9 mg | 2.6 (2.2-3.0) | 1.1 (0.7-1.5) | 5.0 (3.8-6.1) | 2.8 (2.1-3.4) | 1.8 (0.5-3.0) | 4.0 (2.2-5.8) |
| 960.0-3,054.9 mg | 2.9 (2.5-3.2) | 1.3 (0.8-1.7) | 4.6 (3.8-5.4) | 3.2 (2.3-4.1) | 0.8 (0.0-2.0) | 1.3 (0.0-2.7) |
| 3,055.0-7,299.9 mg | 3.7 (3.3-4.2) | 1.4 (0.9-2.0) | 4.6 (3.9-5.3) | 4.9 (3.7-6.1) | 2.4 (0.5-4.2) | 4.4 (1.4-7.3) |
| ≥7,300.0 mg | 3.6 (3.2-4.0) | 1.5 (0.9-2.1) | 4.5 (3.9-5.1) | 3.2 (2.5-3.9) | 3.9 (1.9-5.8) | 5.2 (3.0-7.4) |
| **Cerebrovascular events** | **All disease** | **Inflammatory**  **bowel disease** | **PMR and/or GCA** | **Rheumatoid arthritis** | **Systemic lupus erythematosus** | **Vasculitis** |
| Incident CVD, n (%) | 2,947 | 403 | 1,454 | 898 | 81 | 111 |
| **Cumulative probability (95% CI) at 1 year** | 0.4 (0.4-0.5) | 0.2 (0.1-0.2) | 0.7 (0.6-0.8) | 0.4 (0.3-0.5) | 0.2 (0.1-0.4) | 0.3 (0.2-0.5) |
| **Current daily PED** |  |  |  |  |  |  |
| non-use | 0.3 (0.2-0.3) | 0.1 (0.1-0.2) | 0.5 (0.4-0.7) | 0.3 (0.3-0.4) | 0.2 (0.0-0.3) | 0.3 (0.1-0.4) |
| >0.0-4.9 mg | 0.7 (0.5-0.9) | 0.6 (0.0-1.1) | 0.7 (0.5-0.9) | 1.0 (0.4-1.7) | 0.0 (0.0-0.0) | 0.0 (0.0-0.0) |
| 5.0-14.9 mg | 0.7 (0.6-0.9) | 0.3 (0.0-0.6) | 0.8 (0.6-1.1) | 0.6 (0.3-0.8) | 0.5 (0.0-1.1) | 1.0 (0.1-1.8) |
| 15.0-24.9 mg | 1.2 (0.8-1.7) | 0.9 (0.0-2.0) | 1.5 (0.8-2.1) | 1.2 (0.0-2.5) | 0.0 (0.0-0.0) | 0.0 (0.0-0.0) |
| ≥25.0 mg | 1.1 (0.6-1.7) | 0.5 (0.0-1.2) | 1.9 (0.8-3.0) | 0.5 (0.0-1.6) | 0.0 (0.0-0.0) | 1.2 (0.0-3.5) |
| **Cumulative PED** |  |  |  |  |  |  |
| non-use | 0.3 (0.2-0.3) | 0.2 (0.1-0.2) | 0.7 (0.4-0.9) | 0.3 (0.3-0.4) | 0.1 (0.0-0.3) | 0.3 (0.1-0.5) |
| >0.0-959.9 mg | 0.7 (0.6-0.9) | 0.2 (0.0-0.3) | 1.6 (1.1-2.0) | 0.6 (0.3-1.0) | 0.6 (0.0-1.5) | 0.5 (0.0-1.1) |
| 960.0-3,054.9 mg | 0.7 (0.6-0.9) | 0.2 (0.1-0.4) | 1.0 (0.8-1.2) | 0.5 (0.2-0.8) | 0.4 (0.0-1.0) | 0.5 (0.0-1.2) |
| 3,055.0-7,299.9 mg | 0.3 (0.2-0.4) | 0.2 (0.0-0.4) | 0.3 (0.2-0.4) | 0.5 (0.2-0.8) | 0.3 (0.0-0.9) | 0.2 (0.0-0.6) |
| ≥7,300.0 mg | 0.3 (0.1-0.5) | 0.0 (0.0-0.0) | 0.5 (0.1-0.9) | 0.3 (0.0-0.6) | 0.0 (0.0-0.0) | 0.6 (0.0-1.7) |
| **Cumulative probability (95% CI) at 5 years** | 2.1 (2.0-2.2) | 0.9 (0.7-1.0) | 3.7 (3.5-4.0) | 2.0 (1.8-2.2) | 1.4 (1.0-1.8) | 1.6 (1.2-1.9) |
| **Current daily PED** |  |  |  |  |  |  |
| non-use | 1.6 (1.5-1.7) | 0.7 (0.6-0.8) | 2.7 (2.4- 3.0) | 1.7 (1.5-1.9) | 1.3 (0.9-1.8) | 1.4 (1.0-1.8) |
| >0.0-4.9 mg | 3.9 (3.4-4.5) | 1.9 (0.7-3.0) | 4.5 (3.7- 5.3) | 4.0 (2.8-5.3) | 1.5 (0.0-3.4) | 2.1 (0.0-4.4) |
| 5.0-14.9 mg | 4.2 (3.7-4.6) | 1.7 (0.9-2.4) | 6.2 (5.3- 7.0) | 3.0 (2.3-3.6) | 2.1 (0.6-3.6) | 3.2 (1.3-5.0) |
| 15.0-24.9 mg | 4.6 (3.4-5.9) | 5.4 (2.3-8.5) | 5.8 (3.8- 7.8) | 3.1 (0.8-5.4) | 0.0 (0.0-0.0) | 0.0 (0.0-0.0) |
| ≥25.0 mg | 5.1 (3.5-6.6) | 2.6 (0.8-4.3) | 10.2 (6.0-14.1) | 4.6 (1.5-7.7) | 0.0 (0.0-0.0) | 1.1 (0.0-3.3) |
| **Cumulative PED** |  |  |  |  |  |  |
| non-use | 1.6 (1.4-1.7) | 0.9 (0.7-1.1) | 3.2 (2.5-3.9) | 1.9 (1.6-2.1) | 1.2 (0.7-1.7) | 1.6 (1.1-2.1) |
| >0.0-959.9 mg | 2.1 (1.9-2.4) | 0.8 (0.5-1.1) | 5.2 (4.3-6.2) | 1.8 (1.4-2.3) | 1.5 (0.3-2.6) | 1.1 (0.3-2.0) |
| 960.0-3,054.9 mg | 2.9 (2.6-3.2) | 0.9 (0.6-1.2) | 4.6 (3.9-5.2) | 3.2 (2.3-4.0) | 3.1 (0.8-5.3) | 2.1 (0.4-3.7) |
| 3,055.0-7,299.9 mg | 3.1 (2.7-3.4) | 0.5 (0.2-0.8) | 4.1 (3.6-4.7) | 3.0 (2.2-3.8) | 2.3 (0.2-4.3) | 1.1 (0.0-2.2) |
| ≥7,300.0 mg | 1.8 (1.6-2.1) | 0.9 (0.4-1.4) | 2.3 (1.9-2.7) | 1.5 (1.0-2.0) | 0.5 (0.0-1.1) | 1.8 (0.6-3.0) |
| **Cumulative probability (95% CI) at 10 years** | 4.4 (4.2-4.6) | 1.9 (1.6-2.1) | 7.8 (7.3-8.2) | 4.6 (4.2-4.9) | 2.3 (1.7-2.9) | 3.2 (2.5-3.8) |
| **Current daily PED** |  |  |  |  |  |  |
| non-use | 3.6 (3.5-3.8) | 1.7 (1.4-1.9) | 6.5 (6.0-7.0) | 4.0 (3.6-4.3) | 2.1 (1.5-2.8) | 2.9 (2.2-3.6) |
| >0.0-4.9 mg | 7.6 (6.6-8.5) | 2.3 (0.9-3.7) | 9.0 (7.5-10.4) | 8.1 (6.1-10.0) | 3.8 (0.0-7.5) | 3.1 (0.0-6.2) |
| 5.0-14.9 mg | 7.6 (6.9-8.3) | 3.2 (1.9-4.6) | 11.3 (9.9-12.7) | 6.3 (5.3-7.4) | 3.6 (1.6-5.5) | 5.1 (2.4-7.8) |
| 15.0-24.9 mg | 6.4 (4.7-8.2) | 6.8 (3.0-10.4) | 8.5 (5.4-11.4) | 4.4 (1.1-7.5) | 0.0 (0.0-0.0) | 0.0 (0.0-0.0) |
| ≥25.0 mg | 7.3 (5.4-9.1) | 3.6 (1.5-5.6) | 15.2 (9.6-20.3) | 6.9 (3.2-10.4) | 0.0 (0.0-0.0) | 4.6 (0.0-11.4) |
| **Cumulative PED** |  |  |  |  |  |  |
| non-use | 3.3 (3.0-3.5) | 1.8 (1.5-2.1) | 6.2 (5.0-7.4) | 4.2 (3.8-4.7) | 2.2 (1.4-3.0) | 3.0 (2.1-3.8) |
| >0.0-959.9 mg | 4.5 (4.0-4.9) | 2.2 (1.6-2.8) | 10.0 (8.3-11.5) | 4.3 (3.4-5.1) | 2.2 (0.7-3.6) | 3.5 (1.6-5.4) |
| 960.0-3,054.9 mg | 4.9 (4.4-5.4) | 1.7 (1.2-2.2) | 9.3 (8.1-10.4) | 4.2 (3.2-5.2) | 3.1 (0.9-5.2) | 1.7 (0.3-3.1) |
| 3,055.0-7,299.9 mg | 6.2 (5.6-6.7) | 1.6 (1.0-2.2) | 8.7 (7.8- 9.7) | 6.2 (4.8-7.5) | 3.5 (0.7-6.3) | 5.1 (1.6-8.4) |
| ≥7,300.0 mg | 4.6 (4.2-5.0) | 1.8 (1.2-2.4) | 5.9 (5.2- 6.6) | 4.8 (4.0-5.6) | 1.9 (0.6-3.2) | 2.9 (1.3-4.4) |
| **Abdominal aortic aneurysm events** | **All disease** | **Inflammatory**  **bowel disease** | **PMR and/or GCA** | **Rheumatoid arthritis** | **Systemic lupus erythematosus** | **Vasculitis** |
| Incident CVD, n (%) | 698 | 106 | 338 | 199 | 12 | 43 |
| **Cumulative probability (95% CI) at 1 year** | 0.1 (0.1-0.1) | 0.0 (0.0-0.1) | 0.2 (0.1-0.2) | 0.1 (0.1-0.2) | 0.0 (0.0-0.1) | 0.1 (0.0-0.2) |
| **Current daily PED** |  |  |  |  |  |  |
| non-use | 0.1 (0.0-0.1) | 0.0 (0.0-0.0) | 0.1 (0.0-0.2) | 0.1 (0.1-0.1) | 0.0 (0.0-0.1) | 0.1 (0.0-0.2) |
| >0.0-4.9 mg | 0.3 (0.1-0.4) | 0.4 (0.0-0.9) | 0.3 (0.1-0.4) | 0.1 (0.0-0.3) | 0.0 (0.0-0.0) | 1.0 (0.0-2.4) |
| 5.0-14.9 mg | 0.2 (0.1-0.2) | 0.1 (0.0-0.2) | 0.2 (0.1-0.3) | 0.2 (0.1-0.4) | 0.0 (0.0-0.0) | 0.2 (0.0-0.5) |
| 15.0-24.9 mg | 0.2 (0.0-0.4) | 0.0 (0.0-0.0) | 0.3 (0.0-0.6) | 0.0 (0.0-0.0) | 0.0 (0.0-0.0) | 0.0 (0.0-0.0) |
| ≥25.0 mg | 0.2 (0.0-0.5) | 0.2 (0.0-0.7) | 0.2 (0.0-0.5) | 0.6 (0.0-1.8) | 0.0 (0.0-0.0) | 0.0 (0.0-0.0) |
| **Cumulative PED** |  |  |  |  |  |  |
| non-use | 0.1 (0.0-0.1) | 0.0 (0.0-0.1) | 0.1 (0.0-0.2) | 0.1 (0.1-0.2) | 0.0 (0.0-0.1) | 0.1 (0.0-0.1) |
| >0.0-959.9 mg | 0.2 (0.1-0.3) | 0.0 (0.0-0.1) | 0.3 (0.1-0.6) | 0.2 (0.0-0.4) | 0.0 (0.0-0.0) | 0.5 (0.0-1.0) |
| 960.0-3,054.9 mg | 0.2 (0.1-0.3) | 0.1 (0.0-0.2) | 0.2 (0.1-0.4) | 0.1 (0.0-0.3) | 0.0 (0.0-0.0) | 0.5 (0.0-1.2) |
| 3,055.0-7,299.9 mg | 0.1 (0.0-0.1) | 0.1 (0.0-0.2) | 0.1 (0.0-0.1) | 0.1 (0.0-0.2) | 0.0 (0.0-0.0) | 0.0 (0.0-0.0) |
| ≥7,300.0 mg | 0.0 (0.0-0.1) | 0.0 (0.0-0.0) | 0.1 (0.0-0.3) | 0.0 (0.0-0.0) | 0.0 (0.0-0.0) | 0.0 (0.0-0.0) |
| **Cumulative probability (95% CI) at 5 years** | 0.4 (0.4-0.5) | 0.2 (0.1-0.3) | 0.8 (0.6-0.9) | 0.4 (0.3-0.5) | 0.1 (0.0-0.2) | 0.5 (0.3-0.8) |
| **Current daily PED** |  |  |  |  |  |  |
| non-use | 0.3 (0.2-0.3) | 0.2 (0.1-0.2) | 0.4 (0.3-0.6) | 0.3 (0.2-0.4) | 0.1 (0.0-0.2) | 0.5 (0.2-0.7) |
| >0.0-4.9 mg | 1.1 (0.8-1.4) | 1.3 (0.3-2.3) | 1.3 (0.9-1.8) | 0.6 (0.1-1.1) | 0.0 (0.0-0.0) | 1.0 (0.0-2.4) |
| 5.0-14.9 mg | 1.0 (0.8-1.3) | 0.4 (0.0-0.8) | 1.4 (1.0-1.8) | 1.0 (0.6-1.4) | 0.2 (0.0-0.7) | 0.5 (0.0-1.2) |
| 15.0-24.9 mg | 1.4 (0.7-2.1) | 0.0 (0.0-0.0) | 1.6 (0.5-2.7) | 2.0 (0.0-3.9) | 0.0 (0.0-0.0) | 2.9 (0.0-6.8) |
| ≥25.0 mg | 0.6 (0.1-1.0) | 0.2 (0.0-0.6) | 1.3 (0.0-2.6) | 0.4 (0.0-1.1) | 0.0 (0.0-0.0) | 0.0 (0.0-0.0) |
| **Cumulative PED** |  |  |  |  |  |  |
| non-use | 0.3 (0.2-0.4) | 0.2 (0.1-0.3) | 0.5 (0.2-0.8) | 0.3 (0.2-0.4) | 0.1 (0.0-0.2) | 0.5 (0.2-0.8) |
| >0.0-959.9 mg | 0.4 (0.3-0.5) | 0.1 (0.0-0.3) | 1.0 (0.6-1.4) | 0.3 (0.1-0.5) | 0.2 (0.0-0.7) | 0.6 (0.0-1.2) |
| 960.0-3,054.9 mg | 0.6 (0.5-0.8) | 0.2 (0.1-0.4) | 1.0 (0.7-1.3) | 0.5 (0.2-0.8) | 0.0 (0.0-0.0) | 1.2 (0.0-2.5) |
| 3,055.0-7,299.9 mg | 0.7 (0.5-0.8) | 0.2 (0.0-0.4) | 0.8 (0.6-1.1) | 0.8 (0.4-1.2) | 0.0 (0.0-0.0) | 0.3 (0.0-1.0) |
| ≥7,300.0 mg | 0.4 (0.3-0.6) | 0.1 (0.0-0.3) | 0.5 (0.3-0.7) | 0.4 (0.2-0.7) | 0.2 (0.0-0.6) | 0.4 (0.0-0.9) |
| **Cumulative probability (95% CI) at 10 years** | 1.0 (0.9-1.1) | 0.5 (0.4-0.7) | 1.8 (1.6-2.0) | 0.9 (0.7-1.0) | 0.3 (0.1-0.5) | 1.1 (0.7-1.6) |
| **Current daily PED** |  |  |  |  |  |  |
| non-use | 0.8 (0.7-0.9) | 0.5 (0.4-0.6) | 1.3 (1.1-1.5) | 0.7 (0.5-0.8) | 0.2 (0.0-0.4) | 1.1 (0.6-1.5) |
| >0.0-4.9 mg | 2.4 (1.8-3.0) | 1.1 (0.3-1.9) | 3.2 (2.3-4.1) | 1.6 (0.7-2.5) | 1.0 (0.0-3.0) | 2.2 (0.0-5.0) |
| 5.0-14.9 mg | 2.0 (1.6-2.4) | 1.1 (0.3-1.8) | 3.1 (2.3-4.0) | 1.7 (1.2-2.2) | 0.6 (0.0-1.6) | 0.9 (0.0-2.1) |
| 15.0-24.9 mg | 1.7 (0.9-2.6) | 0.0 (0.0-0.0) | 1.8 (0.5-2.9) | 3.0 (0.4-5.5) | 0.0 (0.0-0.0) | 2.8 (0.0-6.6) |
| ≥25.0 mg | 1.2 (0.2-2.1) | 0.9 (0.0-2.5) | 2.0 (0.0-4.0) | 1.1 (0.0-2.8) | 0.0 (0.0-0.0) | 0.0 (0.0-0.0) |
| **Cumulative PED** |  |  |  |  |  |  |
| non-use | 0.7 (0.6-0.9) | 0.7 (0.5-0.9) | 1.6 (0.9-2.3) | 0.7 (0.5-0.9) | 0.2 (0.0-0.4) | 1.0 (0.4-1.6) |
| >0.0-959.9 mg | 1.0 (0.7-1.2) | 0.6 (0.2-0.9) | 2.3 (1.3-3.2) | 0.7 (0.3-1.0) | 0.5 (0.0-1.2) | 1.8 (0.4-3.2) |
| 960.0-3,054.9 mg | 1.1 (0.8-1.3) | 0.4 (0.2-0.6) | 1.8 (1.3-2.3) | 1.2 (0.6-1.8) | 0.0 (0.0-0.0) | 1.0 (0.0-2.0) |
| 3,055.0-7,299.9 mg | 1.3 (1.0-1.5) | 0.4 (0.1-0.7) | 1.7 (1.3-2.1) | 1.5 (0.8-2.1) | 0.6 (0.0-1.9) | 0.3 (0.0-1.0) |
| ≥7,300.0 mg | 1.2 (1.0-1.5) | 0.4 (0.1-0.7) | 1.7 (1.3-2.1) | 1.1 (0.7-1.5) | 0.4 (0.0-1.1) | 1.0 (0.1-2.0) |

Note: CI, confidence interval; CVD, cardiovascular disease; GCA, giant cell arteritis; IQR, interquartile range; PED, prednisolone-equivalent dose; PMR, polymyalgia rheumatica.

**Table F. Cumulative incidence estimates of cardiovascular diseases per level of current daily and cumulative oral glucocorticoid prednisolone-equivalent dose by type of immune-mediated inflammatory disease in men**

|  | **Immune-mediated inflammatory disease** | | | | |  |
| --- | --- | --- | --- | --- | --- | --- |
| **All CVD events** | **All disease** | **Inflammatory**  **bowel disease** | **PMR and/or GCA** | **Rheumatoid arthritis** | **Systemic lupus erythematosus** | **Vasculitis** |
| Incident CVD, n (%) | 4739 | 1005 | 1932 | 1402 | 84 | 317 |
| **Cumulative probability (95% CI) at 1 year** | 2.7 (2.6-2.9) | 1.1 (0.9-1.3) | 5.5 (5.0-6.0) | 2.8 (2.4-3.2) | 1.3 (0.4-2.3) | 3.7 (2.9-4.5) |
| **Current daily PED** |  |  |  |  |  |  |
| non-use | 1.6 (1.4-1.8) | 0.8 (0.6-1.0) | 2.8 (2.2-3.4) | 2.1 (1.8- 2.5) | 1.4 (0.4-2.4) | 3.1 (2.3-3.9) |
| >0.0-4.9 mg | 4.6 (3.7-5.5) | 3.2 (1.3-5.0) | 5.4 (4.3-6.6) | 2.7 (0.7-4.7) | 7.5 (0.0-20.6) | 2.1 (0.0-4.8) |
| 5.0-14.9 mg | 5.7 (5.0-6.4) | 1.8 (0.9-2.8) | 7.4 (6.3-8.5) | 5.1 (3.6-6.5) | 0.0 (0.0-0.0) | 6.5 (3.3-9.5) |
| 15.0-24.9 mg | 9.1 (7.0-11.1) | 5.8 (2.0-9.4) | 11.0 (8.0-14.0) | 9.7 (3.1-15.8) | 0.0 (0.0-0.0) | 5.1 (0.0-10.5) |
| ≥25.0 mg | 10.9 (8.1-13.6) | 6.5 (3.0-9.9) | 12.4 (7.6-16.9) | 19.5 (7.9-29.7) | 0.0 (0.0-0.0) | 16.0 (3.3-27.0) |
| **Cumulative PED** |  |  |  |  |  |  |
| non-use | 1.7 (1.5-1.9) | 0.9 (0.7-1.1) | 3.5 (2.4-4.7) | 2.2 (1.8-2.6) | 1.3 (0.3-2.3) | 3.4 (2.4-4.3) |
| >0.0-959.9 mg | 4.7 (4.0-5.4) | 1.6 (1.0-2.2) | 9.7 (7.6-11.7) | 5.9 (4.0-7.7) | 2.8 (0.0-8.0) | 7.1 (3.6-10.5) |
| 960.0-3,054.9 mg | 5.3 (4.7-5.9) | 1.3 (0.8-1.9) | 8.6 (7.5-9.7) | 4.0 (2.5-5.5) | 2.9 (0.0-8.3) | 4.3 (1.3-7.2) |
| 3,055.0-7,299.9 mg | 2.4 (1.9-2.8) | 0.9 (0.2-1.6) | 2.6 (2.0-3.2) | 3.1 (1.6-4.5) | 0.0 (0.0-0.0) | 3.2 (1.0-5.3) |
| ≥7,300.0 mg | 1.1 (0.4-1.8) | 0.4 (0.0-1.2) | 1.3 (0.0-2.7) | 1.8 (0.0-3.5) | 0.0 (0.0-0.0) | 1.1 (0.0-3.1) |
| **Cumulative probability (95% CI) at 5 years** | 11.2 (10.8-11.6) | 4.8 (4.4-5.2) | 20.5 (19.5-21.5) | 13.0 (12.1-13.8) | 9.9 (7.2-12.5) | 11.3 (9.8-12.8) |
| **Current daily PED** |  |  |  |  |  |  |
| non-use | 8.0 (7.6-8.4) | 4.0 (3.6-4.4) | 13.6 (12.5-14.7) | 10.5 (9.6-11.4) | 8.6 (5.9-11.2) | 9.6 (8.0-11.1) |
| >0.0-4.9 mg | 23.0 (20.7-25.3) | 8.6 (5.2-11.9) | 30.6 (27.1-33.9) | 17.6 (12.8-22.2) | 18.9 (0.0-36.1) | 16.8 (6.5-25.9) |
| 5.0-14.9 mg | 24.5 (22.8-26.1) | 10.4 (7.7-13.1) | 34.3 (31.3-37.2) | 22.7 (19.6-25.7) | 13.8 (2.8-23.5) | 18.8 (13.1-24.1) |
| 15.0-24.9 mg | 27.8 (23.3-32.0) | 11.3 (5.8-16.4) | 38.6 (31.0-45.4) | 30.0 (17.9-40.1) | 37.3 (0.0-66.0) | 24.7 (7.7-38.3) |
| ≥25.0 mg | 28.1 (23.2-32.7) | 17.3 (11.3-22.9) | 43.0 (30.6-52.8) | 41.0 (27.8-51.5) | 93.3 (NA-NA) | 15.0 (3.1-25.3) |
| **Cumulative PED** |  |  |  |  |  |  |
| non-use | 8.2 (7.6-8.7) | 4.9 (4.3-5.5) | 16.2 (13.3-19.0) | 11.1 (10.0-12.2) | 8.2 (5.2-11.0) | 10.3 (8.4-12.2) |
| >0.0-959.9 mg | 11.0 (9.9-12.0) | 5.3 (4.2-6.5) | 24.3 (20.7-27.6) | 12.0 (9.7-14.3) | 14.0 (3.7-23.1) | 11.2 (7.0-15.3) |
| 960.0-3,054.9 mg | 15.2 (14.1-16.4) | 4.8 (3.7-5.8) | 26.6 (24.3-28.8) | 18.6 (15.0-22.1) | 8.9 (0.0-20.2) | 15.0 (8.1-21.4) |
| 3,055.0-7,299.9 mg | 15.6 (14.4-16.7) | 4.0 (2.8-5.2) | 20.1 (18.2-21.9) | 22.4 (18.6-26.0) | 22.2 (2.8-37.5) | 15.0 (9.2-20.4) |
| ≥7,300.0 mg | 10.9 (9.8-12.0) | 3.6 (2.3-4.9) | 14.8 (12.9-16.7) | 11.6 (9.2-14.0) | 12.8 (2.6-21.9) | 10.1 (6.3-13.8) |
| **Cumulative probability (95% CI) at 10 years** | 20.1 (19.5-20.7) | 9.8 (9.1-10.5) | 34.5 (33.0-35.9) | 24.6 (23.3-25.8) | 15.8 (12.1-19.4) | 19.0 (16.8-21.2) |
| **Current daily PED** |  |  |  |  |  |  |
| non-use | 15.7 (15.1-16.3) | 8.5 (7.8-9.2) | 27.1 (25.4-28.8) | 20.9 (19.5-22.3) | 14.5 (10.5-18.2) | 16.1 (13.7-18.4) |
| >0.0-4.9 mg | 36.0 (32.9-39.0) | 19.7 (13.5-25.4) | 44.1 (39.4-48.3) | 32.3 (25.6-38.2) | 27.8 (0.2-46.6) | 35.4 (18.4-48.1) |
| 5.0-14.9 mg | 36.7 (34.6-38.8) | 18.7 (14.7-22.6) | 51.3 (47.3-54.8) | 35.2 (31.5-38.6) | 16.9 (3.9-27.9) | 30.3 (22.7-37.0) |
| 15.0-24.9 mg | 46.2 (39.0-52.3) | 19.9 (9.4-29.0) | 54.0 (43.0-62.3) | 73.2 (47.0-82.3) | 34.6 (0.0-57.9) | 37.8 (9.1-56.0) |
| ≥25.0 mg | 40.9 (34.8-46.2) | 27.7 (19.5-34.9) | 61.3 (47.9-70.1) | 46.7 (34.3-56.2) | 21.6 (0.0-49.7) | 20.6 (3.1-34.4) |
| **Cumulative PED** |  |  |  |  |  |  |
| non-use | 15.8 (14.9-16.7) | 10.3 (9.3-11.4) | 26.4 (22.3-30.3) | 21.9 (20.1-23.6) | 14.6 (10.1-18.9) | 16.3 (13.3-19.1) |
| >0.0-959.9 mg | 19.8 (18.2-21.4) | 10.2 (8.4-12.0) | 40.4 (35.2-45.1) | 23.9 (20.3-27.3) | 23.3 (6.8-36.4) | 22.6 (15.8-28.8) |
| 960.0-3,054.9 mg | 21.9 (20.4-23.4) | 9.2 (7.6-10.9) | 37.5 (34.3-40.5) | 27.4 (23.0-31.5) | 9.4 (0.0-19.1) | 18.1 (10.1-25.3) |
| 3,055.0-7,299.9 mg | 24.8 (23.2-26.4) | 7.9 (6.1-9.7) | 34.2 (31.4-36.8) | 35.4 (30.4-39.9) | 27.8 (5.6-44.0) | 21.3 (13.4-28.3) |
| ≥7,300.0 mg | 22.7 (21.2-24.2) | 9.3 (7.3-11.2) | 32.0 (29.2-34.7) | 25.0 (21.8-28.1) | 15.7 (4.7-25.3) | 22.1 (16.2-27.4) |
| **Atrial fibrillation events** | **All disease** | **Inflammatory**  **bowel disease** | **PMR and/or GCA** | **Rheumatoid arthritis** | **Systemic lupus erythematosus** | **Vasculitis** |
| Incident CVD, n (%) | 2077 | 461 | 932 | 525 | 27 | 132 |
| **Cumulative probability (95% CI) at 1 year** | 1.1 (1.0-1.3) | 0.5 (0.4-0.6) | 2.4 (2.1-2.8) | 0.9 (0.7-1.1) | 0.8 (0.1-1.6) | 1.4 (0.9-1.9) |
| **Current daily PED** |  |  |  |  |  |  |
| non-use | 0.6 (0.5-0.7) | 0.3 (0.2-0.4) | 1.0 (0.6-1.3) | 0.8 (0.5-1.0) | 0.8 (0.0-1.5) | 1.3 (0.7-1.8) |
| >0.0-4.9 mg | 1.9 (1.3-2.4) | 1.7 (0.3-3.1) | 2.2 (1.5-3.0) | 0.4 (0.0-1.1) | 7.5 (0.0-20.6) | 0.0 (0.0-0.0) |
| 5.0-14.9 mg | 2.5 (2.0-3.0) | 1.1 (0.4-1.8) | 3.6 (2.8-4.4) | 1.3 (0.5-2.0) | 0.0 (0.0-0.0) | 2.1 (0.3-3.8) |
| 15.0-24.9 mg | 4.7 (3.2-6.3) | 2.7 (0.1-5.3) | 5.1 (2.9-7.2) | 7.2 (1.5-12.7) | 0.0 (0.0-0.0) | 5.0 (0.0-10.4) |
| ≥25.0 mg | 5.6 (3.5-7.7) | 6.0 (2.6-9.2) | 6.1 (2.5-9.5) | 4.5 (0.0-10.5) | 0.0 (0.0-0.0) | 3.2 (0.0-9.0) |
| **Cumulative PED** |  |  |  |  |  |  |
| non-use | 0.7 (0.6-0.8) | 0.4 (0.3-0.5) | 2.0 (1.1-2.8) | 0.8 (0.5-1.0) | 0.6 (0.0-1.4) | 1.4 (0.8-2.0) |
| >0.0-959.9 mg | 1.6 (1.2-2.0) | 1.1 (0.6-1.6) | 2.6 (1.5-3.7) | 1.4 (0.5-2.3) | 2.8 (0.0-8.0) | 2.5 (0.3-4.6) |
| 960.0-3,054.9 mg | 2.6 (2.2-3.1) | 0.9 (0.4-1.3) | 4.2 (3.4-5.1) | 1.6 (0.6-2.5) | 2.9 (0.0-8.2) | 1.7 (0.0-3.5) |
| 3,055.0-7,299.9 mg | 0.9 (0.6-1.2) | 0.3 (0.0-0.6) | 1.1 (0.7-1.5) | 0.9 (0.1-1.7) | 0.0 (0.0-0.0) | 1.2 (0.0-2.5) |
| ≥7,300.0 mg | 0.2 (0.0-0.6) | 0.0 (0.0-0.0) | 0.4 (0.0-1.2) | 0.4 (0.0-1.3) | 0.0 (0.0-0.0) | 0.0 (0.0-0.0) |
| **Cumulative probability (95% CI) at 5 years** | 4.5 (4.2-4.7) | 2.0 (1.7-2.3) | 9.1 (8.4-9.9) | 4.4 (3.9-5.0) | 2.0 (0.8-3.2) | 4.7 (3.7-5.7) |
| **Current daily PED** |  |  |  |  |  |  |
| non-use | 3.0 (2.7-3.2) | 1.6 (1.3-1.8) | 5.4 (4.7-6.1) | 3.4 (2.9-3.9) | 1.9 (0.7-3.2) | 4.4 (3.3-5.5) |
| >0.0-4.9 mg | 10.5 (8.8-12.2) | 3.2 (1.2-5.2) | 15.5 (12.7-18.2) | 5.9 (2.9-8.8) | 5.5 (0.0-15.4) | 6.6 (0.1-12.6) |
| 5.0-14.9 mg | 10.8 (9.6-12.1) | 4.8 (2.8-6.7) | 16.9 (14.4-19.3) | 9.4 (7.1-11.5) | 0.0 (0.0-0.0) | 5.0 (1.8-8.0) |
| 15.0-24.9 mg | 13.5 (10.0-16.9) | 4.7 (0.9-8.3) | 19.8 (13.1-25.9) | 14.3 (5.0-22.7) | 18.7 (0.0-45.3) | 14.8 (1.5-26.3) |
| ≥25.0 mg | 13.2 (9.4-16.9) | 12.3 (7.2-17.2) | 22.9 (11.7-32.6) | 8.5 (0.7-15.6) | 0.0 (0.0-0.0) | 2.7 (0.0-7.7) |
| **Cumulative PED** |  |  |  |  |  |  |
| non-use | 3.0 (2.7-3.4) | 1.8 (1.5-2.2) | 7.8 (5.7-9.9) | 3.5 (2.9-4.1) | 1.3 (0.1-2.4) | 5.3 (3.9-6.8) |
| >0.0-959.9 mg | 4.6 (3.9-5.4) | 2.7 (1.9-3.5) | 10.5 (7.9-13.1) | 4.0 (2.6-5.4) | 7.8 (0.1-14.8) | 4.9 (2.0-7.7) |
| 960.0-3,054.9 mg | 6.7 (5.9-7.5) | 2.3 (1.6-3.0) | 12.1 (10.4-13.8) | 7.3 (4.9-9.7) | 3.1 (0.0-8.9) | 5.1 (0.9-9.1) |
| 3,055.0-7,299.9 mg | 6.5 (5.6-7.3) | 1.5 (0.7-2.2) | 8.9 (7.6-10.2) | 8.4 (5.8-10.9) | 5.9 (0.0-16.3) | 5.1 (1.5-8.5) |
| ≥7,300.0 mg | 4.2 (3.5-4.9) | 1.5 (0.7-2.4) | 6.3 (5.0-7.6) | 4.2 (2.7-5.7) | 0.0 (0.0-0.0) | 1.6 (0.0-3.2) |
| **Cumulative probability (95% CI) at 10 years** | 8.8 (8.4-9.3) | 4.2 (3.8-4.7) | 17.7 (16.4-18.9) | 9.3 (8.4-10.2) | 5.5 (3.1-7.7) | 7.4 (5.9-8.9) |
| **Current daily PED** |  |  |  |  |  |  |
| non-use | 6.7 (6.3-7.1) | 3.5 (3.1-4.0) | 13.2 (11.9-14.6) | 7.8 (6.8-8.7) | 6.1 (3.4-8.7) | 6.6 (5.0-8.2) |
| >0.0-4.9 mg | 17.5 (14.9-20.0) | 7.8 (3.4-12.0) | 25.5 (21.0-29.6) | 10.7 (6.3-14.8) | 3.6 (0.0-10.3) | 15.4 (3.2-25.9) |
| 5.0-14.9 mg | 17.0 (15.2-18.7) | 8.3 (5.4-11.1) | 28.3 (24.3-32.0) | 14.4 (11.6-17.0) | 0.0 (0.0-0.0) | 10.1 (4.7-15.1) |
| 15.0-24.9 mg | 21.7 (15.5-27.4) | 8.9 (1.4-15.8) | 27.3 (16.6-36.4) | 35.3 (12.8-51.0) | 12.4 (0.0-31.9) | 13.3 (1.4-23.6) |
| ≥25.0 mg | 24.2 (18.5-29.4) | 20.2 (13.1-26.7) | 41.8 (26.3-53.4) | 18.5 (7.5-27.9) | 0.0 (0.0-0.0) | 2.5 (0.0-7.2) |
| **Cumulative PED** |  |  |  |  |  |  |
| non-use | 6.7 (6.1-7.3) | 4.2 (3.5-4.9) | 14.9 (11.4-18.3) | 8.5 (7.3-9.8) | 5.8 (2.7-8.8) | 8.5 (6.2-10.7) |
| >0.0-959.9 mg | 8.5 (7.3-9.7) | 4.8 (3.5-6.0) | 19.7 (15.2-23.9) | 9.8 (7.2-12.3) | 8.9 (1.0-16.1) | 5.8 (2.4-9.0) |
| 960.0-3,054.9 mg | 10.4 (9.2-11.5) | 4.4 (3.2-5.5) | 19.7 (16.9-22.5) | 10.7 (7.7-13.6) | 2.4 (0.0-7.0) | 7.8 (1.9-13.3) |
| 3,055.0-7,299.9 mg | 11.1 (9.8-12.3) | 3.3 (2.1-4.5) | 17.1 (14.8-19.3) | 12.2 (8.9-15.4) | 11.7 (0.0-25.5) | 6.3 (1.7-10.6) |
| ≥7,300.0 mg | 9.9 (8.8-11.0) | 4.1 (2.7-5.4) | 16.5 (14.2-18.7) | 8.5 (6.4-10.4) | 0.0 (0.0-0.0) | 5.7 (2.4-8.9) |
| **Heart failure events** | **All disease** | **Inflammatory**  **bowel disease** | **PMR and/or GCA** | **Rheumatoid arthritis** | **Systemic lupus erythematosus** | **Vasculitis** |
| Incident CVD, n (%) | 1,514 | 253 | 658 | 466 | 35 | 103 |
| **Cumulative probability (95% CI) at 1 year** | 0.7 (0.6-0.8) | 0.2 (0.1-0.3) | 1.5 (1.2-1.8) | 0.6 (0.4-0.8) | 0.3 (0.0-0.8) | 1.0 (0.6-1.5) |
| **Current daily PED** |  |  |  |  |  |  |
| non-use | 0.3 (0.3-0.4) | 0.2 (0.1-0.2) | 0.7 (0.4-1.0) | 0.3 (0.2-0.5) | 0.4 (0.0-0.9) | 0.8 (0.4-1.2) |
| >0.0-4.9 mg | 1.2 (0.7-1.6) | 0.3 (0.0-0.9) | 1.4 (0.8-2.0) | 0.8 (0.0-1.8) | 0.0 (0.0-0.0) | 2.0 (0.0-4.8) |
| 5.0-14.9 mg | 1.7 (1.3-2.1) | 0.7 (0.1-1.3) | 2.2 (1.6-2.8) | 1.7 (0.8-2.6) | 0.0 (0.0-0.0) | 1.7 (0.0-3.2) |
| 15.0-24.9 mg | 2.3 (1.2-3.4) | 1.4 (0.0-3.3) | 3.0 (1.3-4.7) | 2.5 (0.0-5.8) | 0.0 (0.0-0.0) | 0.0 (0.0-0.0) |
| ≥25.0 mg | 3.6 (1.9-5.2) | 1.0 (0.0-2.4) | 4.9 (1.7-8.0) | 6.1 (0.0-12.5) | 0.0 (0.0-0.0) | 7.7 (0.0-15.6) |
| **Cumulative PED** |  |  |  |  |  |  |
| non-use | 0.3 (0.2-0.4) | 0.2 (0.1-0.2) | 0.6 (0.1-1.1) | 0.4 (0.2-0.5) | 0.4 (0.0-1.0) | 0.9 (0.4-1.5) |
| >0.0-959.9 mg | 1.3 (0.9-1.7) | 0.5 (0.2-0.8) | 2.8 (1.6-3.9) | 1.7 (0.7-2.7) | 0.0 (0.0-0.0) | 1.4 (0.0-3.0) |
| 960.0-3,054.9 mg | 1.5 (1.2-1.8) | 0.5 (0.1-0.8) | 2.5 (1.8-3.1) | 0.8 (0.1-1.5) | 0.0 (0.0-0.0) | 1.6 (0.0-3.5) |
| 3,055.0-7,299.9 mg | 0.7 (0.4-0.9) | 0.0 (0.0-0.0) | 0.8 (0.5-1.2) | 0.9 (0.1-1.7) | 0.0 (0.0-0.0) | 0.8 (0.0-1.8) |
| ≥7,300.0 mg | 0.4 (0.0-0.8) | 0.0 (0.0-0.0) | 0.4 (0.0-1.2) | 0.4 (0.0-1.3) | 0.0 (0.0-0.0) | 1.0 (0.0-3.0) |
| **Cumulative probability (95% CI) at 5 years** | 3.1 (2.8-3.3) | 1.0 (0.8-1.2) | 6.4 (5.7-7.0) | 3.5 (3.0-3.9) | 3.6 (1.9-5.2) | 2.7 (1.9-3.4) |
| **Current daily PED** |  |  |  |  |  |  |
| non-use | 1.9 (1.7-2.1) | 0.7 (0.6-0.9) | 3.8 (3.2-4.4) | 2.4 (2.0-2.9) | 2.6 (1.1-4.1) | 1.9 (1.2-2.6) |
| >0.0-4.9 mg | 6.8 (5.4-8.2) | 1.9 (0.2-3.5) | 9.3 (7.0-11.5) | 5.3 (2.5-7.9) | 0.0 (0.0-0.0) | 8.4 (0.8-15.4) |
| 5.0-14.9 mg | 8.3 (7.2-9.4) | 2.9 (1.4-4.4) | 12.8 (10.6-15.0) | 7.5 (5.5-9.4) | 7.3 (0.0-14.9) | 4.5 (1.5-7.5) |
| 15.0-24.9 mg | 9.8 (6.7-12.8) | 3.0 (0.0-6.0) | 14.5 (8.7-19.9) | 11.2 (2.5-19.0) | 31.2 (0.0-58.2) | 4.3 (0.0-12.0) |
| ≥25.0 mg | 12.9 (9.0-16.7) | 7.7 (3.1-12.1) | 19.7 (9.3-28.8) | 19.4 (7.6-29.5) | 93.7 (NA-NA) | 10.2 (0.0-19.3) |
| **Cumulative PED** |  |  |  |  |  |  |
| non-use | 1.9 (1.6-2.2) | 0.9 (0.7-1.2) | 3.9 (2.3-5.5) | 2.8 (2.3-3.4) | 2.9 (1.1-4.7) | 2.2 (1.3-3.1) |
| >0.0-959.9 mg | 2.8 (2.2-3.4) | 1.3 (0.7-1.8) | 6.7 (4.6-8.8) | 3.4 (2.1-4.7) | 2.2 (0.0-6.3) | 2.1 (0.2-4.0) |
| 960.0-3,054.9 mg | 4.0 (3.4-4.7) | 0.8 (0.4-1.2) | 8.4 (6.9-9.9) | 3.2 (1.6-4.8) | 4.1 (0.0-11.5) | 5.0 (0.8-8.9) |
| 3,055.0-7,299.9 mg | 4.8 (4.1-5.5) | 1.2 (0.5-1.9) | 6.2 (5.1-7.3) | 7.1 (4.7-9.3) | 10.2 (0.0-22.6) | 4.5 (1.1-7.9) |
| ≥7,300.0 mg | 3.7 (3.1-4.4) | 1.0 (0.3-1.7) | 5.3 (4.1-6.4) | 3.8 (2.4-5.2) | 6.3 (0.0-12.9) | 2.6 (0.7-4.4) |
| **Cumulative probability (95% CI) at 10 years** | 6.5 (6.1-6.9) | 2.5 (2.1-2.8) | 12.5 (11.4-13.6) | 8.0 (7.2-8.9) | 6.9 (4.2-9.5) | 6.7 (5.1-8.3) |
| **Current daily PED** |  |  |  |  |  |  |
| non-use | 4.3 (3.9-4.6) | 1.9 (1.6-2.3) | 8.3 (7.2-9.4) | 5.5 (4.7-6.2) | 6.2 (3.4-8.9) | 4.8 (3.3-6.2) |
| >0.0-4.9 mg | 14.1 (11.6-16.6) | 7.2 (2.8-11.5) | 16.9 (13.0-20.7) | 14.9 (9.5-19.9) | 0.0 (0.0-0.0) | 17.7 (3.9-29.1) |
| 5.0-14.9 mg | 15.7 (14.0-17.5) | 5.4 (3.1-7.6) | 23.5 (19.6-27.2) | 15.9 (12.9-18.7) | 11.1 (0.0-21.5) | 13.1 (6.8-18.8) |
| 15.0-24.9 mg | 23.4 (17.1-29.2) | 9.1 (1.3-16.3) | 28.4 (17.8-37.4) | 36.7 (16.7-51.0) | 21.7 (0.0-43.1) | 19.5 (0.0-37.8) |
| ≥25.0 mg | 19.2 (14.1-24.0) | 11.1 (5.4-16.3) | 34.5 (20.0-45.9) | 18.8 (8.7-27.7) | 21.7 (0.0-49.8) | 15.4 (0.0-28.5) |
| **Cumulative PED** |  |  |  |  |  |  |
| non-use | 4.2 (3.7-4.7) | 2.7 (2.1-3.2) | 7.7 (5.0-10.3) | 5.8 (4.8-6.8) | 4.9 (2.2-7.5) | 4.9 (3.0-6.8) |
| >0.0-959.9 mg | 6.2 (5.2-7.2) | 2.3 (1.4-3.1) | 15.3 (11.0-19.3) | 7.7 (5.5-9.9) | 16.1 (0.0-30.0) | 7.5 (2.9-11.9) |
| 960.0-3,054.9 mg | 6.6 (5.7-7.6) | 1.8 (1.0-2.5) | 14.1 (11.5-16.6) | 7.4 (4.8-10.0) | 2.9 (0.0-8.3) | 7.2 (1.8-12.3) |
| 3,055.0-7,299.9 mg | 8.7 (7.6-9.8) | 2.1 (1.1-3.0) | 11.6 (9.7-13.5) | 14.3 (10.4-18.1) | 23.6 (0.0-41.7) | 11.2 (4.1-17.8) |
| ≥7,300.0 mg | 8.9 (7.9-9.9) | 3.1 (1.9-4.2) | 12.3 (10.4-14.2) | 10.8 (8.5-13.0) | 7.9 (0.0-15.3) | 8.2 (4.3-11.9) |
| **Acute myocardial infarction events** | **All disease** | **Inflammatory**  **bowel disease** | **PMR and/or GCA** | **Rheumatoid arthritis** | **Systemic lupus erythematosus** | **Vasculitis** |
| Incident CVD, n (%) | 1,163 | 251 | 428 | 396 | 25 | 63 |
| **Cumulative probability (95% CI) at 1 year** | 0.4 (0.4-0.5) | 0.2 (0.1-0.3) | 0.7 (0.5-0.9) | 0.6 (0.4-0.7) | 0.3 (0.0-0.8) | 0.6 (0.2-0.9) |
| **Current daily PED** |  |  |  |  |  |  |
| non-use | 0.3 (0.2-0.4) | 0.1 (0.1-0.2) | 0.5 (0.2-0.7) | 0.4 (0.3-0.6) | 0.4 (0.0-0.9) | 0.4 (0.1-0.7) |
| >0.0-4.9 mg | 0.3 (0.1-0.6) | 0.0 (0.0-0.0) | 0.4 (0.1-0.7) | 0.4 (0.0-1.1) | 0.0 (0.0-0.0) | 0.0 (0.0-0.0) |
| 5.0-14.9 mg | 1.0 (0.7-1.3) | 0.4 (0.0-0.8) | 1.2 (0.7-1.6) | 1.3 (0.5-2.0) | 0.0 (0.0-0.0) | 0.8 (0.0-1.9) |
| 15.0-24.9 mg | 0.9 (0.2-1.5) | 0.7 (0.0-2.1) | 1.2 (0.2-2.3) | 0.0 (0.0-0.0) | 0.0 (0.0-0.0) | 0.0 (0.0-0.0) |
| ≥25.0 mg | 2.4 (1.0-3.8) | 1.5 (0.0-3.3) | 1.7 (0.0-3.6) | 4.1 (0.0-9.6) | 0.0 (0.0-0.0) | 8.0 (0.0-16.3) |
| **Cumulative PED** |  |  |  |  |  |  |
| non-use | 0.3 (0.2-0.4) | 0.2 (0.1-0.3) | 0.5 (0.1-0.9) | 0.4 (0.3-0.6) | 0.4 (0.0-1.0) | 0.4 (0.1-0.8) |
| >0.0-959.9 mg | 0.8 (0.5-1.1) | 0.1 (0.0-0.2) | 1.7 (0.8-2.6) | 1.3 (0.4-2.2) | 0.0 (0.0-0.0) | 1.5 (0.0-3.1) |
| 960.0-3,054.9 mg | 0.7 (0.4-0.9) | 0.1 (0.0-0.3) | 1.0 (0.6-1.4) | 0.8 (0.1-1.4) | 0.0 (0.0-0.0) | 0.5 (0.0-1.5) |
| 3,055.0-7,299.9 mg | 0.5 (0.3-0.7) | 0.5 (0.0-1.0) | 0.4 (0.1-0.6) | 0.7 (0.0-1.4) | 0.0 (0.0-0.0) | 0.8 (0.0-1.9) |
| ≥7,300.0 mg | 0.0 (0.0-0.0) | 0.0 (0.0-0.0) | 0.0 (0.0-0.0) | 0.0 (0.0-0.0) | 0.0 (0.0-0.0) | 0.0 (0.0-0.0) |
| **Cumulative probability (95% CI) at 5 years** | 2.6 (2.4-2.8) | 1.2 (1.0-1.4) | 4.6 (4.0-5.1) | 3.3 (2.8-3.7) | 2.7 (1.2-4.2) | 1.9 (1.2-2.5) |
| **Current daily PED** |  |  |  |  |  |  |
| non-use | 1.9 (1.7-2.1) | 1.0 (0.8-1.2) | 3.1 (2.5-3.6) | 2.8 (2.3-3.3) | 2.6 (1.1-4.2) | 1.3 (0.7-1.9) |
| >0.0-4.9 mg | 5.1 (3.8-6.4) | 1.7 (0.0-3.3) | 6.8 (4.7-8.8) | 4.2 (1.6-6.7) | 8.5 (0.0-23.1) | 4.6 (0.0-9.6) |
| 5.0-14.9 mg | 6.2 (5.2-7.2) | 3.5 (1.8-5.2) | 8.5 (6.6-10.4) | 5.8 (4.0-7.5) | 2.5 (0.0-7.1) | 4.3 (1.3-7.2) |
| 15.0-24.9 mg | 5.5 (2.9-7.9) | 1.6 (0.0-3.9) | 9.3 (4.0-14.3) | 5.0 (0.0-10.5) | 0.0 (0.0-0.0) | 4.1 (0.0-11.5) |
| ≥25.0 mg | 6.1 (3.5-8.6) | 4.5 (1.4-7.6) | 10.9 (3.0-18.1) | 4.3 (0.0-9.0) | 0.0 (0.0-0.0) | 6.9 (0.0-14.1) |
| **Cumulative PED** |  |  |  |  |  |  |
| non-use | 1.9 (1.6-2.2) | 1.0 (0.7-1.3) | 3.5 (2.0-4.9) | 3.2 (2.6-3.8) | 2.9 (1.1-4.7) | 0.9 (0.3-1.5) |
| >0.0-959.9 mg | 2.2 (1.7-2.7) | 1.6 (0.9-2.2) | 3.4 (1.9-4.8) | 2.3 (1.2-3.3) | 2.1 (0.0-6.0) | 3.4 (0.8-6.0) |
| 960.0-3,054.9 mg | 3.2 (2.6-3.8) | 1.1 (0.5-1.6) | 5.6 (4.3-6.9) | 4.8 (2.7-6.9) | 0.0 (0.0-0.0) | 2.7 (0.0-5.8) |
| 3,055.0-7,299.9 mg | 3.9 (3.3-4.6) | 1.4 (0.7-2.1) | 4.9 (3.9-5.9) | 5.8 (3.6-7.9) | 4.9 (0.0-13.8) | 3.4 (0.7-6.0) |
| ≥7,300.0 mg | 2.8 (2.2-3.4) | 1.4 (0.6-2.2) | 4.0 (3.0-5.0) | 2.1 (1.0-3.1) | 2.2 (0.0-6.3) | 2.6 (0.7-4.6) |
| **Cumulative probability (95% CI) at 10 years** | 5.1 (4.8-5.5) | 2.6 (2.3-3.0) | 8.3 (7.4-9.2) | 6.9 (6.1-7.7) | 5.1 (2.7-7.4) | 4.2 (3.0-5.4) |
| **Current daily PED** |  |  |  |  |  |  |
| non-use | 3.9 (3.5-4.2) | 2.1 (1.8-2.5) | 5.7 (4.8-6.6) | 5.8 (4.9-6.6) | 5.5 (2.8-8.0) | 3.2 (2.0-4.3) |
| >0.0-4.9 mg | 9.1 (7.1-11.1) | 4.5 (1.2-7.7) | 11.4 (8.1-14.6) | 9.7 (5.3-13.8) | 4.8 (0.0-13.5) | 4.4 (0.0-9.2) |
| 5.0-14.9 mg | 11.5 (10.0-13.0) | 7.2 (4.4-9.9) | 16.2 (13.0-19.3) | 11.0 (8.5-13.4) | 2.1 (0.0-6.0) | 9.4 (4.2-14.2) |
| 15.0-24.9 mg | 13.9 (8.1-19.4) | 8.0 (0.0-16.4) | 13.7 (5.8-20.9) | 23.1 (5.4-37.0) | 0.0 (0.0-0.0) | 13.7 (0.0-31.3) |
| ≥25.0 mg | 12.0 (7.7-16.1) | 9.5 (3.8-14.8) | 21.4 (8.9-31.9) | 8.5 (1.0-15.4) | 0.0 (0.0-0.0) | 6.6 (0.0-13.6) |
| **Cumulative PED** |  |  |  |  |  |  |
| non-use | 3.9 (3.4-4.4) | 2.3 (1.8-2.8) | 6.8 (4.2-9.4) | 6.0 (5.0-7.0) | 6.2 (3.0-9.3) | 2.4 (1.2-3.7) |
| >0.0-959.9 mg | 4.7 (3.8-5.6) | 2.7 (1.8-3.6) | 7.0 (4.0-9.8) | 6.2 (4.2-8.2) | 4.4 (0.0-10.2) | 8.5 (3.8-13.0) |
| 960.0-3,054.9 mg | 5.0 (4.2-5.9) | 2.1 (1.2-2.9) | 9.1 (6.9-11.1) | 7.0 (4.5-9.5) | 0.0 (0.0-0.0) | 3.9 (0.0-7.8) |
| 3,055.0-7,299.9 mg | 6.5 (5.6-7.5) | 3.3 (2.1-4.4) | 7.8 (6.3- 9.3) | 11.0 (7.6-14.3) | 4.6 (0.0-12.9) | 4.3 (0.9-7.6) |
| ≥7,300.0 mg | 6.5 (5.6-7.4) | 3.5 (2.2-4.7) | 8.5 (6.9-10.1) | 7.5 (5.5-9.4) | 1.6 (0.0-4.8) | 5.2 (2.1-8.2) |
| **Peripheral arterial disease events** | **All disease** | **Inflammatory**  **bowel disease** | **PMR and/or GCA** | **Rheumatoid arthritis** | **Systemic lupus erythematosus** | **Vasculitis** |
| Incident CVD, n (%) | 745 | 143 | 290 | 229 | 18 | 65 |
| **Cumulative probability (95% CI) at 1 year** | 0.4 (0.3-0.4) | 0.1 (0.1-0.2) | 0.7 (0.5-0.9) | 0.4 (0.3-0.6) | 0.0 (0.0-0.0) | 0.6 (0.3-0.9) |
| **Current daily PED** |  |  |  |  |  |  |
| non-use | 0.2 (0.2-0.3) | 0.1 (0.0-0.1) | 0.3 (0.1-0.5) | 0.4 (0.2-0.5) | 0.0 (0.0-0.0) | 0.6 (0.2-0.9) |
| >0.0-4.9 mg | 0.5 (0.2-0.7) | 0.3 (0.0-0.9) | 0.5 (0.2-0.9) | 0.4 (0.0-1.1) | 0.0 (0.0-0.0) | 0.0 (0.0-0.0) |
| 5.0-14.9 mg | 0.9 (0.6-1.2) | 0.3 (0.0-0.6) | 1.3 (0.8-1.7) | 0.6 (0.1-1.1) | 0.0 (0.0-0.0) | 1.2 (0.0-2.6) |
| 15.0-24.9 mg | 1.4 (0.5-2.2) | 1.2 (0.0-2.9) | 1.7 (0.4-2.9) | 1.2 (0.0-3.6) | 0.0 (0.0-0.0) | 0.0 (0.0-0.0) |
| ≥25.0 mg | 0.9 (0.0-1.8) | 0.0 (0.0-0.0) | 1.1 (0.0-2.7) | 4.6 (0.0-10.7) | 0.0 (0.0-0.0) | 0.0 (0.0-0.0) |
| **Cumulative PED** |  |  |  |  |  |  |
| non-use | 0.2 (0.2-0.3) | 0.1 (0.0-0.2) | 0.5 (0.1-0.9) | 0.3 (0.2-0.5) | 0.0 (0.0-0.0) | 0.6 (0.2-1.1) |
| >0.0-959.9 mg | 0.7 (0.4-1.0) | 0.2 (0.0-0.5) | 1.0 (0.3-1.8) | 1.0 (0.2-1.7) | 0.0 (0.0-0.0) | 1.9 (0.0-3.7) |
| 960.0-3,054.9 mg | 0.7 (0.4-0.9) | 0.0 (0.0-0.0) | 1.3 (0.8-1.7) | 0.5 (0.0-1.0) | 0.0 (0.0-0.0) | 0.0 (0.0-0.0) |
| 3,055.0-7,299.9 mg | 0.3 (0.1-0.4) | 0.0 (0.0-0.0) | 0.3 (0.1-0.5) | 0.5 (0.0-1.1) | 0.0 (0.0-0.0) | 0.0 (0.0-0.0) |
| ≥7,300.0 mg | 0.5 (0.0-0.9) | 0.4 (0.0-1.2) | 0.4 (0.0-1.2) | 0.9 (0.0-2.0) | 0.0 (0.0-0.0) | 0.0 (0.0-0.0) |
| **Cumulative probability (95% CI) at 5 years** | 1.8 (1.6-1.9) | 0.7 (0.5-0.8) | 3.1 (2.6-3.5) | 2.2 (1.8-2.6) | 2.1 (0.8-3.4) | 2.4 (1.6-3.1) |
| **Current daily PED** |  |  |  |  |  |  |
| non-use | 1.3 (1.1-1.5) | 0.6 (0.4-0.7) | 2.1 (1.7-2.6) | 1.8 (1.4-2.2) | 2.0 (0.6-3.3) | 2.0 (1.3- 2.8) |
| >0.0-4.9 mg | 3.4 (2.4-4.4) | 1.2 (0.0-2.5) | 4.8 (3.1-6.4) | 2.8 (0.7-4.8) | 0.0 (0.0-0.0) | 0.0 (0.0- 0.0) |
| 5.0-14.9 mg | 3.7 (2.9-4.5) | 1.3 (0.3-2.2) | 4.7 (3.3-6.0) | 3.6 (2.2-4.9) | 2.4 (0.0-6.9) | 5.8 (2.3- 9.1) |
| 15.0-24.9 mg | 6.0 (3.4-8.4) | 3.0 (0.0-5.8) | 8.9 (3.7-13.8) | 5.0 (0.1-9.6) | 18.7 (0.0-45.3) | 4.1 (0.0-11.5) |
| ≥25.0 mg | 4.6 (2.0-7.2) | 0.7 (0.0-1.9) | 8.3 (0.4-15.4) | 13.9 (3.1-23.4) | 0.0 (0.0-0.0) | 0.0 (0.0- 0.0) |
| **Cumulative PED** |  |  |  |  |  |  |
| non-use | 1.4 (1.1-1.6) | 0.8 (0.6-1.1) | 2.6 (1.3-3.8) | 1.7 (1.3-2.2) | 1.8 (0.3-3.2) | 2.1 (1.2-3.1) |
| >0.0-959.9 mg | 1.6 (1.1-2.0) | 0.4 (0.1-0.7) | 3.7 (2.1-5.2) | 1.9 (0.9-2.8) | 4.7 (0.0-10.9) | 3.2 (0.8-5.6) |
| 960.0-3,054.9 mg | 2.2 (1.7-2.7) | 0.5 (0.1-0.8) | 4.3 (3.2-5.4) | 3.3 (1.6-5.0) | 0.0 (0.0-0.0) | 1.0 (0.0-2.9) |
| 3,055.0-7,299.9 mg | 2.5 (2.0-3.0) | 0.5 (0.1-0.9) | 3.0 (2.2-3.8) | 5.0 (2.9-6.9) | 4.2 (0.0-12.0) | 2.2 (0.0-4.7) |
| ≥7,300.0 mg | 1.8 (1.4-2.3) | 0.6 (0.1-1.1) | 2.1 (1.4-2.8) | 2.2 (1.1-3.3) | 2.2 (0.0-6.3) | 3.1 (0.9-5.2) |
| **Cumulative probability (95% CI) at 10 years** | 3.2 (3.0-3.5) | 1.4 (1.1-1.7) | 5.7 (4.9-6.4) | 3.9 (3.3-4.5) | 3.6 (1.7-5.4) | 4.4 (3.2-5.6) |
| **Current daily PED** |  |  |  |  |  |  |
| non-use | 2.5 (2.3-2.8) | 1.2 (0.9- 1.5) | 4.6 (3.7-5.4) | 3.3 (2.7-3.9) | 2.6 (1.0-4.2) | 3.6 (2.4-4.8) |
| >0.0-4.9 mg | 6.8 (5.1-8.6) | 5.4 (1.6- 9.1) | 8.0 (5.3-10.6) | 5.7 (2.5-8.7) | 10.8 (0.0-28.3) | 3.5 (0.0-9.9) |
| 5.0-14.9 mg | 6.2 (5.1-7.4) | 2.2 (0.7- 3.6) | 8.3 (5.9-10.6) | 5.8 (4.0-7.7) | 5.7 (0.0-13.2) | 10.1 (4.7-15.2) |
| 15.0-24.9 mg | 6.6 (3.5-9.6) | 5.6 (0.0-11.6) | 7.3 (3.1-11.3) | 4.4 (0.1-8.6) | 26.0 (0.0-50.3) | 3.3 (0.0-9.5) |
| ≥25.0 mg | 5.3 (2.5-8.0) | 1.6 (0.0- 4.0) | 11.6 (1.7-20.4) | 9.2 (2.5-15.3) | 0.0 (0.0-0.0) | 0.0 (0.0-0.0) |
| **Cumulative PED** |  |  |  |  |  |  |
| non-use | 2.5 (2.1-2.9) | 1.5 (1.1-1.9) | 3.2 (1.7-4.7) | 3.6 (2.8-4.4) | 2.6 (0.8-4.4) | 3.4 (2.0-4.8) |
| >0.0-959.9 mg | 3.0 (2.3-3.7) | 1.3 (0.6-2.0) | 8.5 (5.3-11.6) | 2.8 (1.5-4.0) | 4.1 (0.0-9.5) | 4.9 (1.6-8.1) |
| 960.0-3,054.9 mg | 3.3 (2.6-3.9) | 1.4 (0.8-2.1) | 5.8 (4.2-7.4) | 4.5 (2.5-6.4) | 3.6 (0.0-10.3) | 0.9 (0.0-2.5) |
| 3,055.0-7,299.9 mg | 4.6 (3.8-5.5) | 1.3 (0.5-2.1) | 6.6 (5.0-8.2) | 7.7 (4.9-10.5) | 4.0 (0.0-11.2) | 3.6 (0.0-7.2) |
| ≥7,300.0 mg | 3.6 (2.9-4.2) | 1.1 (0.4-1.8) | 4.7 (3.4-5.9) | 3.7 (2.3-5.0) | 6.8 (0.0-14.0) | 7.6 (3.7-11.3) |
| **Cerebrovascular disease events** | **All disease** | **Inflammatory**  **bowel disease** | **PMR and/or GCA** | **Rheumatoid arthritis** | **Systemic lupus erythematosus** | **Vasculitis** |
| Incident CVD, n (%) | 916 | 188 | 415 | 250 | 9 | 54 |
| **Cumulative probability (95% CI) at 1 year** | 0.4 (0.3-0.5) | 0.2 (0.1-0.2) | 0.9 (0.6-1.1) | 0.4 (0.2-0.5) | 0.0 (0.0-0.0) | 0.4 (0.1-0.7) |
| **Current daily PED** |  |  |  |  |  |  |
| non-use | 0.3 (0.2-0.3) | 0.1 (0.1-0.2) | 0.6 (0.3-0.8) | 0.3 (0.2-0.5) | 0.0 (0.0-0.0) | 0.4 (0.1-0.6) |
| >0.0-4.9 mg | 0.9 (0.5-1.3) | 0.3 (0.0-0.9) | 1.1 (0.6-1.6) | 0.8 (0.0-1.9) | 0.0 (0.0-0.0) | 0.0 (0.0-0.0) |
| 5.0-14.9 mg | 0.7 (0.4-0.9) | 0.0 (0.0-0.0) | 1.0 (0.6-1.4) | 0.5 (0.0-0.9) | 0.0 (0.0-0.0) | 0.8 (0.0-2.0) |
| 15.0-24.9 mg | 1.4 (0.5-2.3) | 1.9 (0.0-4.0) | 1.5 (0.3-2.6) | 1.3 (0.0-3.8) | 0.0 (0.0-0.0) | 0.0 (0.0-0.0) |
| ≥25.0 mg | 0.8 (0.0-1.6) | 0.0 (0.0-0.0) | 1.1 (0.0-2.6) | 1.9 (0.0-5.5) | 0.0 (0.0-0.0) | 2.6 (0.0-7.4) |
| **Cumulative PED** |  |  |  |  |  |  |
| non-use | 0.3 (0.2-0.3) | 0.2 (0.1-0.3) | 0.4 (0.0-0.8) | 0.3 (0.2-0.5) | 0.0 (0.0-0.0) | 0.4 (0.1-0.8) |
| >0.0-959.9 mg | 0.9 (0.5-1.2) | 0.2 (0.0-0.4) | 2.5 (1.4-3.6) | 0.8 (0.1-1.4) | 0.0 (0.0-0.0) | 0.5 (0.0-1.4) |
| 960.0-3,054.9 mg | 0.6 (0.4-0.8) | 0.2 (0.0-0.4) | 1.1 (0.7-1.5) | 0.2 (0.0-0.5) | 0.0 (0.0-0.0) | 0.5 (0.0-1.5) |
| 3,055.0-7,299.9 mg | 0.4 (0.2-0.6) | 0.0 (0.0-0.0) | 0.4 (0.2-0.6) | 0.7 (0.0-1.4) | 0.0 (0.0-0.0) | 0.4 (0.0-1.2) |
| ≥7,300.0 mg | 0.1 (0.0-0.4) | 0.0 (0.0-0.0) | 0.4 (0.0-1.2) | 0.0 (0.0-0.0) | 0.0 (0.0-0.0) | 0.0 (0.0-0.0) |
| **Cumulative probability (95% CI) at 5 years** | 2.0 (1.8-2.2) | 0.9 (0.7-1.1) | 4.0 (3.5-4.5) | 2.2 (1.8-2.6) | 1.2 (0.2-2.2) | 1.8 (1.2-2.5) |
| **Current daily PED** |  |  |  |  |  |  |
| non-use | 1.6 (1.4-1.8) | 0.8 (0.6-1.0) | 2.9 (2.4-3.4) | 2.0 (1.6-2.4) | 1.2 (0.1-2.2) | 1.8 (1.1-2.5) |
| >0.0-4.9 mg | 3.8 (2.8-4.9) | 1.5 (0.0-2.9) | 5.3 (3.6-7.0) | 2.8 (0.9-4.7) | 6.7 (0.0-18.4) | 1.6 (0.0-4.6) |
| 5.0-14.9 mg | 3.8 (3.0-4.5) | 1.0 (0.0-1.9) | 6.8 (5.1-8.5) | 2.5 (1.3-3.7) | 0.0 (0.0-0.0) | 2.6 (0.3-4.8) |
| 15.0-24.9 mg | 5.5 (3.2-7.8) | 3.6 (0.4-6.8) | 9.1 (4.1-13.8) | 4.4 (0.0-9.2) | 0.0 (0.0-0.0) | 0.0 (0.0-0.0) |
| ≥25.0 mg | 3.9 (1.5-6.1) | 1.6 (0.0-3.8) | 5.3 (0.0-10.6) | 9.3 (0.5-17.3) | 0.0 (0.0-0.0) | 2.3 (0.0-6.6) |
| **Cumulative PED** |  |  |  |  |  |  |
| non-use | 1.6 (1.3-1.8) | 1.0 (0.7-1.3) | 3.0 (1.7-4.4) | 2.1 (1.6-2.6) | 1.1 (0.0-2.2) | 1.9 (1.1-2.8) |
| >0.0-959.9 mg | 2.3 (1.8-2.8) | 1.1 (0.6-1.6) | 6.8 (4.7-8.9) | 2.0 (1.0-2.9) | 0.0 (0.0-0.0) | 0.8 (0.0-2.0) |
| 960.0-3,054.9 mg | 2.7 (2.1-3.2) | 0.9 (0.4-1.4) | 4.6 (3.4-5.7) | 3.5 (1.7-5.2) | 5.2 (0.0-14.5) | 2.6 (0.0-5.6) |
| 3,055.0-7,299.9 mg | 2.7 (2.1-3.2) | 0.4 (0.0-0.8) | 4.0 (3.0-4.9) | 3.0 (1.5-4.5) | 3.9 (0.0-11.0) | 1.8 (0.0-3.8) |
| ≥7,300.0 mg | 1.9 (1.4-2.3) | 0.5 (0.0-1.0) | 2.8 (1.9-3.6) | 1.7 (0.7-2.6) | 0.0 (0.0-0.0) | 1.7 (0.0-3.3) |
| **Cumulative probability (95% CI) at 10 years** | 4.1 (3.8-4.4) | 1.8 (1.5-2.2) | 8.0 (7.1-8.9) | 4.8 (4.1-5.4) | 1.4 (0.3-2.4) | 3.4 (2.3-4.4) |
| **Current daily PED** |  |  |  |  |  |  |
| non-use | 3.5 (3.2-3.8) | 1.8 (1.4-2.1) | 6.8 (5.8-7.8) | 4.3 (3.6-5.0) | 1.2 (0.1-2.2) | 3.4 (2.2-4.6) |
| >0.0-4.9 mg | 7.9 (6.0-9.8) | 2.1 (0.1-4.1) | 9.7 (6.7-12.7) | 10.1 (5.6-14.3) | 10.4 (0.0-23.1) | 1.5 (0.0-4.4) |
| 5.0-14.9 mg | 6.6 (5.4-7.7) | 2.6 (0.8-4.3) | 12.0 (9.1-14.7) | 5.1 (3.3-6.8) | 0.0 (0.0-0.0) | 3.9 (0.8-6.8) |
| 15.0-24.9 mg | 6.5 (3.7-9.3) | 3.3 (0.3-6.2) | 12.0 (5.5-17.9) | 3.7 (0.0-7.8) | 0.0 (0.0-0.0) | 0.0 (0.0-0.0) |
| ≥25.0 mg | 3.3 (1.5-5.1) | 1.0 (0.0-2.5) | 7.2 (0.9-12.9) | 4.9 (0.5-9.1) | 0.0 (0.0-0.0) | 2.1 (0.0-6.1) |
| **Cumulative PED** |  |  |  |  |  |  |
| non-use | 3.2 (2.7-3.6) | 1.9 (1.4-2.4) | 6.9 (4.2-9.4) | 4.6 (3.7-5.6) | 1.1 (0.0-2.3) | 3.4 (2.0-4.8) |
| >0.0-959.9 mg | 4.2 (3.4-5.1) | 2.2 (1.3-3.1) | 12.4 (8.8-15.8) | 3.6 (2.1-5.2) | 0.0 (0.0-0.0) | 3.9 (0.5-7.1) |
| 960.0-3,054.9 mg | 4.2 (3.4-5.0) | 1.8 (1.0-2.5) | 8.1 (6.1-10.1) | 4.7 (2.6-6.8) | 3.3 (0.0-9.5) | 2.2 (0.0-4.8) |
| 3,055.0-7,299.9 mg | 5.4 (4.5-6.3) | 1.4 (0.6-2.3) | 8.2 (6.5-9.9) | 6.8 (4.0-9.4) | 3.6 (0.0-10.3) | 6.0 (0.6-11.1) |
| ≥7,300.0 mg | 4.3 (3.6-5.0) | 1.4 (0.6-2.1) | 6.7 (5.2-8.1) | 4.9 (3.3-6.4) | 1.9 (0.0-5.6) | 1.8 (0.2-3.4) |
| **Abdominal aortic aneurysm events** | **All disease** | **Inflammatory**  **bowel disease** | **PMR and/or GCA** | **Rheumatoid arthritis** | **Systemic lupus erythematosus** | **Vasculitis** |
| Incident CVD, n (%) | 390 | 82 | 159 | 110 | 9 | 30 |
| **Cumulative probability (95% CI) at 1 year** | 0.2 (0.1-0.2) | 0.1 (0.0-0.1) | 0.3 (0.2-0.5) | 0.2 (0.1-0.3) | 0.2 (0.0-0.5) | 0.2 (0.0-0.4) |
| **Current daily PED** |  |  |  |  |  |  |
| non-use | 0.1 (0.1-0.2) | 0.0 (0.0-0.1) | 0.2 (0.0-0.4) | 0.2 (0.1-0.3) | 0.2 (0.0-0.6) | 0.2 (0.0-0.4) |
| >0.0-4.9 mg | 0.5 (0.2-0.9) | 0.9 (0.0-1.8) | 0.6 (0.2-1.0) | 0.0 (0.0-0.0) | 0.0 (0.0-0.0) | 0.0 (0.0-0.0) |
| 5.0-14.9 mg | 0.3 (0.1-0.4) | 0.1 (0.0-0.4) | 0.2 (0.0-0.4) | 0.4 (0.0-0.9) | 0.0 (0.0-0.0) | 0.4 (0.0-1.1) |
| 15.0-24.9 mg | 0.6 (0.0-1.1) | 0.0 (0.0-0.0) | 1.0 (0.0-2.0) | 0.0 (0.0-0.0) | 0.0 (0.0-0.0) | 0.0 (0.0-0.0) |
| ≥25.0 mg | 0.5 (0.0-1.1) | 0.5 (0.0-1.5) | 0.0 (0.0-0.0) | 2.3 (0.0-6.6) | 0.0 (0.0-0.0) | 0.0 (0.0-0.0) |
| **Cumulative PED** |  |  |  |  |  |  |
| non-use | 0.1 (0.1-0.2) | 0.1 (0.0-0.1) | 0.2 (0.0-0.5) | 0.2 (0.1-0.3) | 0.2 (0.0-0.6) | 0.1 (0.0-0.2) |
| >0.0-959.9 mg | 0.4 (0.2-0.6) | 0.1 (0.0-0.2) | 0.7 (0.1-1.2) | 0.5 (0.0-1.0) | 0.0 (0.0-0.0) | 1.4 (0.0-3.0) |
| 960.0-3,054.9 mg | 0.3 (0.2-0.5) | 0.2 (0.0-0.4) | 0.5 (0.2-0.8) | 0.5 (0.0-1.0) | 0.0 (0.0-0.0) | 0.0 (0.0-0.0) |
| 3,055.0-7,299.9 mg | 0.1 (0.0-0.2) | 0.1 (0.0-0.4) | 0.2 (0.0-0.3) | 0.0 (0.0-0.0) | 0.0 (0.0-0.0) | 0.0 (0.0-0.0) |
| ≥7,300.0 mg | 0.1 (0.0-0.4) | 0.0 (0.0-0.0) | 0.4 (0.0-1.2) | 0.0 (0.0-0.0) | 0.0 (0.0-0.0) | 0.0 (0.0-0.0) |
| **Cumulative probability (95% CI) at 5 years** | 0.8 (0.7-0.9) | 0.4 (0.3-0.5) | 1.6 (1.2-1.9) | 0.9 (0.6-1.1) | 0.5 (0.0-1.1) | 0.8 (0.4-1.2) |
| **Current daily PED** |  |  |  |  |  |  |
| non-use | 0.6 (0.5-0.7) | 0.3 (0.2-0.4) | 1.0 (0.7-1.3) | 0.6 (0.4-0.8) | 0.4 (0.0-1.0) | 0.8 (0.3-1.3) |
| >0.0-4.9 mg | 2.0 (1.2-2.8) | 2.5 (0.6-4.3) | 2.4 (1.2-3.5) | 1.1 (0.0-2.3) | 0.0 (0.0-0.0) | 0.0 (0.0-0.0) |
| 5.0-14.9 mg | 1.9 (1.3-2.4) | 0.8 (0.1-1.6) | 2.8 (1.7-3.9) | 1.8 (0.9-2.8) | 1.8 (0.0-5.1) | 0.4 (0.0-1.1) |
| 15.0-24.9 mg | 3.4 (1.5-5.2) | 0.0 (0.0-0.0) | 4.0 (1.0-6.8) | 6.4 (0.0-12.3) | 0.0 (0.0-0.0) | 6.1 (0.0-14.0) |
| ≥25.0 mg | 1.2 (0.0-2.3) | 0.4 (0.0-1.3) | 3.2 (0.0-7.4) | 1.4 (0.0-4.1) | 0.0 (0.0-0.0) | 0.0 (0.0-0.0) |
| **Cumulative PED** |  |  |  |  |  |  |
| non-use | 0.6 (0.4-0.7) | 0.4 (0.2-0.6) | 1.2 (0.4-2.1) | 0.7 (0.4-1.0) | 0.2 (0.0-0.7) | 0.8 (0.2-1.4) |
| >0.0-959.9 mg | 0.9 (0.6-1.3) | 0.3 (0.0-0.6) | 2.5 (1.2-3.8) | 1.0 (0.2-1.7) | 2.0 (0.0-5.9) | 1.1 (0.0-2.4) |
| 960.0-3,054.9 mg | 1.2 (0.8-1.5) | 0.5 (0.2-0.9) | 2.0 (1.2-2.7) | 1.3 (0.3-2.2) | 0.0 (0.0-0.0) | 1.8 (0.0-4.2) |
| 3,055.0-7,299.9 mg | 1.2 (0.8-1.5) | 0.5 (0.1-0.9) | 1.6 (1.0-2.1) | 1.3 (0.2-2.2) | 0.0 (0.0-0.0) | 0.8 (0.0-2.2) |
| ≥7,300.0 mg | 0.8 (0.5-1.1) | 0.3 (0.0-0.6) | 1.0 (0.5-1.5) | 1.0 (0.2-1.7) | 1.6 (0.0-4.7) | 0.3 (0.0-1.0) |
| **Cumulative probability (95% CI) at 10 years** | 1.7 (1.5-1.9) | 0.9 (0.7-1.1) | 2.9 (2.4-3.5) | 1.9 (1.5-2.4) | 1.3 (0.2-2.4) | 2.0 (1.1-2.9) |
| **Current daily PED** |  |  |  |  |  |  |
| non-use | 1.3 (1.1-1.5) | 0.8 (0.6-1.0) | 2.1 (1.6-2.7) | 1.6 (1.1-2.1) | 1.0 (0.0-2.1) | 1.9 (0.9-2.9) |
| >0.0-4.9 mg | 3.6 (2.4-4.8) | 2.2 (0.5-3.8) | 5.2 (2.9-7.5) | 1.5 (0.0-3.0) | 6.7 (0.0-18.3) | 3.0 (0.0-8.6) |
| 5.0-14.9 mg | 3.2 (2.4-4.1) | 1.5 (0.2-2.8) | 4.9 (2.9-6.9) | 3.3 (1.9-4.7) | 1.5 (0.0-4.4) | 1.4 (0.0-3.6) |
| 15.0-24.9 mg | 3.9 (1.8-5.9) | 0.0 (0.0-0.0) | 4.8 (1.0-8.4) | 7.4 (0.7-13.6) | 0.0 (0.0-0.0) | 5.3 (0.0-12.2) |
| ≥25.0 mg | 1.6 (0.0-3.3) | 1.9 (0.0-4.9) | 2.2 (0.0-5.0) | 0.9 (0.0-2.5) | 0.0 (0.0-0.0) | 0.0 (0.0-0.0) |
| **Cumulative PED** |  |  |  |  |  |  |
| non-use | 1.3 (1.0-1.6) | 1.0 (0.6-1.3) | 2.0 (0.6-3.5) | 1.6 (1.0-2.1) | 0.6 (0.0-1.5) | 1.9 (0.5-3.2) |
| >0.0-959.9 mg | 1.9 (1.3-2.5) | 0.9 (0.2-1.5) | 4.0 (1.8-6.1) | 2.0 (0.8-3.3) | 4.9 (0.0-11.5) | 3.6 (0.3-6.8) |
| 960.0-3,054.9 mg | 2.0 (1.5-2.6) | 0.9 (0.4-1.4) | 3.3 (2.1-4.4) | 3.3 (1.3-5.2) | 0.0 (0.0-0.0) | 1.5 (0.0-3.6) |
| 3,055.0-7,299.9 mg | 1.8 (1.3-2.3) | 0.7 (0.1-1.3) | 2.6 (1.6-3.6) | 2.1 (0.5-3.6) | 6.2 (0.0-17.1) | 0.7 (0.0-2.0) |
| ≥7,300.0 mg | 1.8 (1.4-2.3) | 0.6 (0.1-1.2) | 2.8 (1.8-3.7) | 1.8 (0.9-2.8) | 1.3 (0.0-3.8) | 1.8 (0.0-3.6) |

Note: CI, confidence interval; CVD, cardiovascular disease; GCA, giant cell arteritis; IQR, interquartile range; PED, prednisolone-equivalent dose; PMR, polymyalgia rheumatica

**Table G. Cumulative incidence estimates of cardiovascular diseases per level of current daily and cumulative oral glucocorticoid prednisolone-equivalent dose by type of immune-mediated inflammatory disease in women**

|  | **Immune-mediated inflammatory disease** | | | | |  |
| --- | --- | --- | --- | --- | --- | --- |
| **All CVD events** | **All disease** | **Inflammatory**  **bowel disease** | **PMR and/or GCA** | **Rheumatoid arthritis** | **Systemic lupus erythematosus** | **Vasculitis** |
| Incident CVD, n (%) | 8,687 | 932 | 4,335 | 2,839 | 291 | 294 |
| **Cumulative probability (95% CI) at 1 year** | 2.2 (2.1-2.4) | 0.9 (0.8-1.1) | 3.9 (3.7-4.2) | 1.9 (1.7-2.1) | 1.1 (0.7-1.5) | 2.0 (1.5-2.5) |
| **Current daily PED** |  |  |  |  |  |  |
| non-use | 1.4 (1.2-1.5) | 0.7 (0.6-0.9) | 2.6 (2.2-2.9) | 1.4 (1.2-1.6) | 0.9 (0.5-1.2) | 1.4 (0.9-1.8) |
| >0.0-4.9 mg | 3.4 (2.9-3.9) | 1.7 (0.3-3.0) | 3.3 (2.7-3.9) | 4.2 (2.8-5.7) | 1.7 (0.0-3.9) | 6.4 (1.3-11.3) |
| 5.0-14.9 mg | 4.4 (3.9-4.8) | 2.7 (1.6-3.9) | 5.1 (4.5-5.7) | 3.6 (2.8-4.4) | 1.4 (0.2-2.5) | 3.7 (1.4-6.0) |
| 15.0-24.9 mg | 6.3 (5.0-7.5) | 2.5 (0.0-4.9) | 7.4 (5.8-9.0) | 5.4 (2.1-8.6) | 1.6 (0.0-4.7) | 4.8 (0.0-10.0) |
| ≥25.0 mg | 7.8 (6.0-9.6) | 3.4 (1.1-5.8) | 10.3 (7.4-13.1) | 5.4 (1.4-9.2) | 15.7 (0.3-28.8) | 10.8 (1.4-19.3) |
| **Cumulative PED** |  |  |  |  |  |  |
| no-use | 1.5 (1.3-1.6) | 0.9 (0.7-1.0) | 3.8 (3.1-4.5) | 1.5 (1.3-1.7) | 1.0 (0.6-1.4) | 1.5 (1.0-2.1) |
| >0.0-959.9 mg | 3.6 (3.1-4.0) | 1.1 (0.6-1.5) | 7.1 (6.1-8.2) | 2.5 (1.8-3.2) | 1.4 (0.0-2.7) | 3.3 (1.5-5.0) |
| 960.0-3,054.9 mg | 4.0 (3.6-4.4) | 1.1 (0.6-1.5) | 5.2 (4.6-5.7) | 3.2 (2.4-4.1) | 2.2 (0.4-4.0) | 4.8 (1.8-7.6) |
| 3,055.0-7,299.9 mg | 2.0 (1.7-2.2) | 1.1 (0.3-1.8) | 2.0 (1.6-2.3) | 2.9 (2.0-3.8) | 1.0 (0.0-2.1) | 0.4 (0.0-1.2) |
| ≥7,300.0 mg | 1.6 (1.0-2.2) | 0.7 (0.0-1.9) | 2.2 (1.1-3.2) | 1.0 (0.1-1.9) | 0.0 (0.0-0.0) | 4.4 (0.0-9.2) |
| **Cumulative probability (95% CI) at 5 years** | 9.8 (9.5-10.0) | 3.9 (3.5-4.2) | 16.3 (15.7-16.9) | 8.9 (8.5-9.4) | 5.2 (4.4-6.1) | 7.4 (6.3-8.5) |
| **Current daily PED** |  |  |  |  |  |  |
| non-use | 6.6 (6.4-6.9) | 3.1 (2.7-3.4) | 10.5 (9.8-11.1) | 7.1 (6.7-7.6) | 4.5 (3.6-5.4) | 6.1 (5.0-7.1) |
| >0.0-4.9 mg | 18.3 (17.0-19.7) | 9.9 (5.9-13.7) | 21.4 (19.7-23.2) | 14.1 (11.5-16.7) | 8.7 (3.3-13.7) | 11.7 (4.7-18.0) |
| 5.0-14.9 mg | 20.4 (19.3-21.4) | 10.7 (8.1-13.2) | 27.0 (25.4-28.7) | 16.5 (14.8-18.1) | 6.9 (3.9-9.7) | 14.3 (9.2-19.1) |
| 15.0-24.9 mg | 26.2 (23.0-29.2) | 16.9 (9.2-23.9) | 32.7 (28.2-36.9) | 20.8 (14.0-27.0) | 8.1 (0.0-15.5) | 17.6 (4.1-29.1) |
| ≥25.0 mg | 27.9 (24.3-31.3) | 13.9 (8.9-18.7) | 41.2 (34.5-47.2) | 27.1 (19.5-33.8) | 20.6 (5.5-33.1) | 23.5 (8.0-36.3) |
| **Cumulative PED** |  |  |  |  |  |  |
| non-use | 6.9 (6.6-7.3) | 4.0 (3.5-4.5) | 14.8 (13.2-16.4) | 7.6 (7.0-8.1) | 5.0 (3.8-6.0) | 6.7 (5.3-8.0) |
| >0.0-959.9 mg | 9.1 (8.4-9.7) | 3.2 (2.4-4.0) | 17.7 (15.9-19.5) | 8.3 (7.2-9.5) | 6.2 (3.7-8.7) | 8.5 (5.8-11.2) |
| 960.0-3,054.9 mg | 13.2 (12.4-14.0) | 3.8 (3.0-4.7) | 19.8 (18.4-21.2) | 12.5 (10.7-14.2) | 6.4 (3.1-9.5) | 9.5 (5.2-13.6) |
| 3,055.0-7,299.9 mg | 15.3 (14.4-16.1) | 3.9 (2.8-5.1) | 18.4 (17.3-19.6) | 16.5 (14.4-18.5) | 7.8 (3.9-11.7) | 10.0 (5.1-14.6) |
| ≥7,300.0 mg | 9.1 (8.5-9.8) | 4.4 (2.8-6.0) | 11.0 (10.0-11.9) | 8.0 (6.7-9.3) | 2.7 (0.9-4.4) | 6.4 (3.3-9.4) |
| **Cumulative probability (95% CI) at 10 years** | 18.7 (18.3-19.1) | 7.7 (7.1-8.3) | 30.0 (29.1-30.8) | 18.2 (17.5-18.9) | 10.6 (9.2-12.0) | 13.2 (11.5-14.9) |
| **Current daily PED** |  |  |  |  |  |  |
| non-use | 14.0 (13.6-14.4) | 6.4 (5.8-6.9) | 22.9 (21.9-23.8) | 14.8 (14.1-15.6) | 8.9 (7.4-10.3) | 11.5 (9.8-13.2) |
| >0.0-4.9 mg | 31.8 (29.9-33.6) | 15.0 (9.9-19.7) | 38.0 (35.4-40.5) | 26.3 (22.7-29.7) | 21.5 (11.3-30.3) | 18.3 (8.5-26.8) |
| 5.0-14.9 mg | 34.3 (32.9-35.7) | 18.0 (14.3-21.5) | 45.5 (43.1-47.8) | 30.9 (28.6-33.1) | 14.3 (9.8-18.5) | 21.9 (15.1-28.1) |
| 15.0-24.9 mg | 40.9 (36.5-44.9) | 31.7 (18.7-42.2) | 49.6 (43.2-55.1) | 30.9 (22.6-38.2) | 25.4 (1.6-42.6) | 24.9 (5.7-39.6) |
| ≥25.0 mg | 39.1 (34.6-43.1) | 23.8 (16.7-30.1) | 55.2 (46.4-62.1) | 40.1 (31.2-47.7) | 30.1 (10.4-44.8) | 35.1 (6.8-53.4) |
| **Cumulative PED** |  |  |  |  |  |  |
| non-use | 13.8 (13.2-14.4) | 8.0 (7.1-8.9) | 26.2 (23.7-28.6) | 15.7 (14.8-16.7) | 9.8 (7.8-11.7) | 11.7 (9.5-13.8) |
| >0.0-959.9 mg | 16.7 (15.7-17.7) | 7.0 (5.7-8.3) | 30.6 (27.9-33.2) | 16.9 (15.2-18.6) | 11.1 (7.5-14.5) | 15.0 (10.8-18.9) |
| 960.0-3,054.9 mg | 20.4 (19.3-21.5) | 6.4 (5.2-7.6) | 32.4 (30.4-34.4) | 19.4 (17.1-21.7) | 9.9 (5.4-14.1) | 10.6 (5.9-15.1) |
| 3,055.0-7,299.9 mg | 24.9 (23.7-26.0) | 8.4 (6.7-10.0) | 31.4 (29.7-33.1) | 25.5 (22.8-28.0) | 11.2 (6.1-16.1) | 18.0 (10.5-24.8) |
| ≥7,300.0 mg | 21.7 (20.7-22.7) | 8.0 (6.1-9.8) | 26.8 (25.3-28.2) | 20.6 (18.7-22.4) | 11.1 (7.7-14.5) | 14.9 (10.0-19.4) |
| **Atrial fibrillation events** | **All disease** | **Inflammatory**  **bowel disease** | **PMR and/or GCA** | **Rheumatoid arthritis** | **Systemic lupus erythematosus** | **Vasculitis** |
| Incident CVD, n (%) | 3,936 | 426 | 2,092 | 1,194 | 104 | 122 |
| **Cumulative probability (95% CI) at 1 year** | 3,936 | 426 | 2,092 | 1,194 | 104 | 122 |
| **Current daily PED** | 0.9 (0.8-0.9) | 0.4 (0.3-0.5) | 1.7 (1.5-1.9) | 0.5 (0.4-0.6) | 0.2 (0.1-0.4) | 0.7 (0.4-1.0) |
| non-use |  |  |  |  |  |  |
| >0.0-4.9 mg | 0.5 (0.4-0.5) | 0.3 (0.2-0.4) | 0.9 (0.7-1.1) | 0.4 (0.3-0.5) | 0.2 (0.0-0.3) | 0.6 (0.3-0.9) |
| 5.0-14.9 mg | 1.4 (1.0-1.7) | 0.0 (0.0-0.0) | 1.5 (1.1-1.9) | 1.2 (0.4-2.0) | 0.8 (0.0-2.5) | 3.2 (0.0-6.7) |
| 15.0-24.9 mg | 1.8 (1.5-2.1) | 0.9 (0.2-1.6) | 2.4 (2.0-2.8) | 1.0 (0.6-1.4) | 0.3 (0.0-0.8) | 0.7 (0.0-1.7) |
| ≥25.0 mg | 2.7 (1.8-3.5) | 0.6 (0.0-1.8) | 3.5 (2.4-4.6) | 1.1 (0.0-2.6) | 0.0 (0.0-0.0) | 1.7 (0.0-4.8) |
| **Cumulative PED** | 3.0 (1.8-4.1) | 2.2 (0.3-4.0) | 4.3 (2.4-6.3) | 0.8 (0.0-2.5) | 0.0 (0.0-0.0) | 2.3 (0.0-6.6) |
| non-use |  |  |  |  |  |  |
| >0.0-959.9 mg | 0.5 (0.4-0.6) | 0.4 (0.3-0.5) | 1.5 (1.0-1.9) | 0.4 (0.3-0.5) | 0.2 (0.0-0.4) | 0.7 (0.3-1.0) |
| 960.0-3,054.9 mg | 1.3 (1.0-1.6) | 0.6 (0.3-0.9) | 2.7 (2.0-3.3) | 0.8 (0.4-1.2) | 0.0 (0.0-0.0) | 0.7 (0.0-1.6) |
| 3,055.0-7,299.9 mg | 1.6 (1.4-1.9) | 0.3 (0.1-0.6) | 2.3 (2.0-2.7) | 0.9 (0.4-1.3) | 0.7 (0.0-1.7) | 1.4 (0.0-3.0) |
| ≥7,300.0 mg | 0.9 (0.7-1.1) | 0.1 (0.0-0.4) | 1.1 (0.8-1.3) | 0.9 (0.4-1.5) | 0.0 (0.0-0.0) | 0.0 (0.0-0.0) |
| **Cumulative probability (95% CI) at 5 years** | 0.3 (0.0-0.6) | 0.0 (0.0-0.0) | 0.4 (0.0-0.8) | 0.2 (0.0-0.6) | 0.0 (0.0-0.0) | 1.5 (0.0-4.3) |
| **Current daily PED** | 3.9 (3.7-4.1) | 1.6 (1.3-1.8) | 7.1 (6.7-7.5) | 3.2 (2.9-3.5) | 1.3 (0.9-1.8) | 2.9 (2.2-3.6) |
| non-use |  |  |  |  |  |  |
| >0.0-4.9 mg | 2.6 (2.4-2.7) | 1.3 (1.1-1.5) | 4.3 (3.9-4.7) | 2.5 (2.3-2.8) | 1.3 (0.8-1.8) | 2.5 (1.8-3.2) |
| 5.0-14.9 mg | 8.0 (7.0-8.9) | 3.6 (0.9-6.2) | 9.7 (8.4-11.0) | 5.5 (3.7-7.1) | 2.6 (0.0-5.5) | 5.4 (0.5-10.1) |
| 15.0-24.9 mg | 8.5 (7.7-9.2) | 3.6 (2.0-5.1) | 12.4 (11.1-13.6) | 6.1 (5.0-7.2) | 1.0 (0.0-2.1) | 5.0 (1.7-8.2) |
| ≥25.0 mg | 11.9 (9.5-14.2) | 3.7 (0.0-7.4) | 17.1 (13.4-20.7) | 8.4 (3.6-12.9) | 0.0 (0.0-0.0) | 2.2 (0.0-6.3) |
| **Cumulative PED** | 12.8 (10.0-15.5) | 6.6 (3.1-9.9) | 21.0 (15.0-26.4) | 10.2 (4.6-15.5) | 3.6 (0.0-10.3) | 12.9 (0.0-24.1) |
| non-use |  |  |  |  |  |  |
| >0.0-959.9 mg | 2.6 (2.4-2.8) | 1.7 (1.3-2.0) | 6.6 (5.4-7.7) | 2.6 (2.2-2.9) | 1.5 (0.9-2.1) | 2.8 (2.0-3.7) |
| 960.0-3,054.9 mg | 3.6 (3.1-4.1) | 1.6 (1.0-2.1) | 7.4 (6.1-8.7) | 3.2 (2.4-3.9) | 1.7 (0.3-3.0) | 2.9 (1.3-4.6) |
| 3,055.0-7,299.9 mg | 5.6 (5.0-6.1) | 1.5 (0.9-2.1) | 9.1 (8.1-10.1) | 4.6 (3.4-5.8) | 1.6 (0.0-3.1) | 2.1 (0.0-4.2) |
| ≥7,300.0 mg | 6.6 (6.0-7.2) | 1.4 (0.7-2.1) | 8.5 (7.7-9.4) | 6.3 (4.9-7.6) | 0.0 (0.0-0.0) | 4.8 (1.2-8.3) |
| **Cumulative probability (95% CI) at 10 years** | 3.5 (3.0-3.9) | 0.8 (0.1-1.5) | 4.2 (3.6-4.8) | 3.4 (2.5-4.2) | 0.6 (0.0-1.3) | 2.3 (0.5-4.2) |
| **Current daily PED** | 8.6 (8.3-8.9) | 3.4 (3.1-3.8) | 15.0 (14.3-15.7) | 7.8 (7.3-8.3) | 3.7 (2.8-4.6) | 6.2 (4.9-7.4) |
| non-use |  |  |  |  |  |  |
| >0.0-4.9 mg | 6.4 (6.1-6.7) | 2.9 (2.5-3.3) | 11.3 (10.5-12.1) | 6.2 (5.7-6.7) | 2.9 (2.0-3.8) | 5.5 (4.2-6.8) |
| 5.0-14.9 mg | 16.3 (14.7-17.9) | 6.2 (2.7-9.4) | 19.5 (17.3-21.7) | 13.6 (10.6-16.5) | 9.5 (2.0-16.2) | 9.0 (1.8-15.6) |
| 15.0-24.9 mg | 16.2 (15.0-17.3) | 8.3 (5.6-11.0) | 23.2 (21.0-25.4) | 13.2 (11.4-15.0) | 4.8 (1.9-7.6) | 9.8 (4.5-14.8) |
| ≥25.0 mg | 19.2 (15.5-22.7) | 10.3 (1.6-18.2) | 26.1 (20.2-31.5) | 12.7 (6.8-18.2) | 10.2 (0.0-22.6) | 2.2 (0.0-6.4) |
| **Cumulative PED** | 20.7 (16.6-24.5) | 11.9 (6.1-17.4) | 38.1 (27.9-46.6) | 15.2 (8.3-21.6) | 10.2 (0.0-20.7) | 15.6 (0.6-28.0) |
| non-use |  |  |  |  |  |  |
| >0.0-959.9 mg | 6.1 (5.6-6.5) | 3.8 (3.2-4.4) | 13.9 (11.8-16.0) | 6.5 (5.8-7.2) | 3.1 (1.9-4.2) | 5.5 (3.9-7.1) |
| 960.0-3,054.9 mg | 7.6 (6.9-8.4) | 3.0 (2.2-3.9) | 15.3 (13.0-17.5) | 7.6 (6.3-8.9) | 4.5 (2.0-6.8) | 7.0 (3.8-10.1) |
| 3,055.0-7,299.9 mg | 9.7 (8.8-10.5) | 2.8 (2.0-3.7) | 16.6 (14.9-18.3) | 8.5 (6.8-10.2) | 3.9 (0.7-6.9) | 3.9 (0.4-7.3) |
| ≥7,300.0 mg | 12.2 (11.3-13.2) | 3.5 (2.4-4.7) | 16.7 (15.3-18.2) | 10.9 (8.9-12.9) | 0.9 (0.0-2.7) | 11.0 (4.5-17.0) |
| **Heart failure events** | **All disease** | **Inflammatory**  **bowel disease** | **PMR and/or GCA** | **Rheumatoid arthritis** | **Systemic lupus erythematosus** | **Vasculitis** |
| Incident CVD, n (%) | 3213 | 282 | 1,661 | 1,071 | 103 | 98 |
| **Cumulative probability (95% CI) at 1 year** | 0.7 (0.6-0.8) | 0.2 (0.2-0.3) | 1.2 (1.0-1.4) | 0.6 (0.5-0.7) | 0.4 (0.2-0.6) | 0.5 (0.3-0.8) |
| **Current daily PED** |  |  |  |  |  |  |
| non-use | 0.4 (0.3-0.5) | 0.2 (0.1-0.2) | 0.9 (0.7-1.1) | 0.4 (0.3-0.5) | 0.3 (0.1-0.5) | 0.3 (0.1-0.6) |
| >0.0-4.9 mg | 0.9 (0.6-1.2) | 0.3 (0.0-0.8) | 0.9 (0.6-1.2) | 1.2 (0.4-2.0) | 0.0 (0.0-0.0) | 2.2 (0.0-5.2) |
| 5.0-14.9 mg | 1.4 (1.2-1.6) | 1.3 (0.5-2.1) | 1.6 (1.3-1.9) | 1.3 (0.8-1.7) | 0.3 (0.0-0.8) | 0.3 (0.0-1.0) |
| 15.0-24.9 mg | 1.9 (1.2-2.6) | 0.7 (0.0-1.9) | 2.0 (1.1-2.9) | 3.3 (0.7-5.8) | 0.0 (0.0-0.0) | 1.6 (0.0-4.7) |
| ≥25.0 mg | 2.9 (1.7-4.0) | 0.4 (0.0-1.2) | 2.7 (1.1-4.3) | 5.3 (1.4-9.1) | 8.1 (0.0-18.3) | 7.0 (0.0-14.3) |
| **Cumulative PED** |  |  |  |  |  |  |
| non-use | 0.4 (0.4-0.5) | 0.2 (0.1-0.3) | 1.3 (0.8-1.7) | 0.5 (0.3-0.6) | 0.4 (0.1-0.6) | 0.3 (0.1-0.6) |
| >0.0-959.9 mg | 1.1 (0.8-1.3) | 0.1 (0.0-0.3) | 2.3 (1.7-3.0) | 0.8 (0.4-1.3) | 0.0 (0.0-0.0) | 1.5 (0.3-2.7) |
| 960.0-3,054.9 mg | 1.1 (0.9-1.3) | 0.5 (0.2-0.8) | 1.3 (1.0-1.6) | 1.2 (0.7-1.8) | 0.7 (0.0-1.8) | 0.0 (0.0-0.0) |
| 3,055.0-7,299.9 mg | 0.7 (0.6-0.9) | 0.3 (0.0-0.7) | 0.7 (0.5-1.0) | 1.1 (0.5-1.7) | 0.3 (0.0-1.0) | 0.4 (0.0-1.2) |
| ≥7,300.0 mg | 0.7 (0.3-1.1) | 0.0 (0.0-0.0) | 1.1 (0.3-1.8) | 0.4 (0.0-0.9) | 0.0 (0.0-0.0) | 1.5 (0.0-4.2) |
| **Cumulative probability (95% CI) at 5 years** | 3.3 (3.2-3.5) | 1.1 (0.9-1.3) | 5.7 (5.3-6.1) | 3.1 (2.8-3.4) | 1.6 (1.1-2.1) | 2.3 (1.7-2.9) |
| **Current daily PED** |  |  |  |  |  |  |
| non-use | 2.1 (2.0-2.3) | 0.7 (0.5-0.9) | 3.8 (3.4-4.2) | 2.2 (1.9-2.5) | 1.3 (0.8-1.7) | 1.8 (1.2-2.4) |
| >0.0-4.9 mg | 6.7 (5.8-7.5) | 4.0 (1.5-6.4) | 8.1 (6.9-9.3) | 4.3 (2.8-5.8) | 1.2 (0.0-3.4) | 4.3 (0.0-8.4) |
| 5.0-14.9 mg | 7.5 (6.8-8.2) | 5.0 (3.1-6.8) | 9.5 (8.4-10.7) | 6.8 (5.6-7.9) | 1.8 (0.2-3.3) | 3.5 (0.9-6.0) |
| 15.0-24.9 mg | 10.4 (8.1-12.6) | 4.5 (0.0-8.9) | 11.2 (8.1-14.3) | 13.3 (7.6-18.6) | 6.4 (0.0-13.1) | 8.8 (0.0-18.2) |
| ≥25.0 mg | 10.9 (8.3-13.4) | 6.4 (2.3-10.3) | 11.6 (7.1-15.9) | 12.8 (7.2-18.0) | 17.9 (3.6-29.9) | 14.9 (1.1-26.7) |
| **Cumulative PED** |  |  |  |  |  |  |
| non-use | 2.0 (1.8-2.2) | 0.9 (0.6-1.1) | 5.1 (4.1-6.1) | 2.3 (2.0-2.7) | 1.4 (0.8-2.0) | 1.7 (1.0-2.3) |
| >0.0-959.9 mg | 3.1 (2.7-3.5) | 0.7 (0.3-1.1) | 6.4 (5.3-7.6) | 2.9 (2.2-3.6) | 2.3 (0.7-3.9) | 3.7 (1.9-5.5) |
| 960.0-3,054.9 mg | 4.5 (4.0-5.0) | 1.4 (0.9-1.9) | 6.6 (5.7-7.5) | 5.0 (3.8-6.2) | 1.2 (0.0-2.5) | 2.5 (0.0-4.9) |
| 3,055.0-7,299.9 mg | 5.6 (5.0-6.1) | 1.6 (0.9-2.4) | 6.8 (6.0-7.6) | 6.1 (4.7-7.4) | 2.0 (0.0-3.9) | 3.7 (0.7-6.6) |
| ≥7,300.0 mg | 3.5 (3.1-3.9) | 2.0 (0.9-3.0) | 3.9 (3.3-4.5) | 3.6 (2.7-4.4) | 1.5 (0.2-2.8) | 2.7 (0.7-4.6) |
| **Cumulative probability (95% CI) at 10 years** | 7.0 (6.7-7.3) | 2.2 (1.9-2.5) | 12.1 (11.5-12.8) | 6.7 (6.2-7.2) | 3.9 (3.0-4.9) | 4.7 (3.6-5.8) |
| **Current daily PED** |  |  |  |  |  |  |
| non-use | 5.0 (4.7-5.2) | 1.6 (1.4-1.9) | 8.9 (8.2-9.6) | 5.2 (4.7-5.6) | 3.2 (2.3-4.2) | 3.6 (2.5-4.6) |
| >0.0-4.9 mg | 12.8 (11.3-14.2) | 6.0 (2.6-9.3) | 17.2 (14.9-19.4) | 7.5 (5.3-9.5) | 2.6 (0.0-6.1) | 11.5 (3.0-19.2) |
| 5.0-14.9 mg | 14.2 (13.1-15.3) | 7.1 (4.7-9.5) | 19.4 (17.3-21.5) | 13.0 (11.3-14.7) | 5.2 (2.1-8.1) | 5.8 (1.3-10.0) |
| 15.0-24.9 mg | 19.8 (16.0-23.3) | 9.3 (1.2-16.7) | 24.0 (18.1-29.4) | 16.4 (9.8-22.5) | 12.6 (0.0-24.2) | 30.2 (3.4-48.4) |
| ≥25.0 mg | 19.7 (15.5-23.7) | 12.0 (6.0-17.5) | 23.6 (14.5-31.6) | 21.4 (13.2-28.6) | 26.4 (6.7-41.3) | 30.4 (0.0-50.4) |
| **Cumulative PED** |  |  |  |  |  |  |
| non-use | 4.7 (4.3-5.1) | 2.1 (1.6-2.6) | 9.8 (8.1-11.5) | 5.5 (4.9-6.1) | 3.8 (2.4-5.1) | 4.1 (2.6-5.5) |
| >0.0-959.9 mg | 6.2 (5.5-6.9) | 2.1 (1.4-2.9) | 12.8 (10.7-14.8) | 6.1 (5.0-7.2) | 5.4 (2.6-8.0) | 4.2 (2.0-6.2) |
| 960.0-3,054.9 mg | 8.0 (7.3-8.8) | 1.9 (1.3-2.6) | 13.8 (12.2-15.4) | 8.3 (6.6-9.9) | 2.2 (0.2-4.2) | 2.9 (0.2-5.5) |
| 3,055.0-7,299.9 mg | 9.6 (8.7-10.4) | 2.9 (1.9-3.9) | 12.2 (11.0-13.5) | 10.1 (8.2-12.0) | 4.5 (0.9-8.1) | 7.4 (2.2-12.3) |
| ≥7,300.0 mg | 8.4 (7.8-9.1) | 2.6 (1.6-3.7) | 10.9 (9.8-12.0) | 7.5 (6.3-8.7) | 3.5 (1.5-5.4) | 7.2 (3.5-10.8) |
| **Acute myocardial infarction events** | **All disease** | **Inflammatory**  **bowel disease** | **PMR and/or GCA** | **Rheumatoid arthritis** | **Systemic lupus erythematosus** | **Vasculitis** |
| Incident CVD, n (%) | 1,646 | 129 | 795 | 617 | 61 | 45 |
| **Cumulative probability (95% CI) at 1 year** | 0.3 (0.3-0.4) | 0.1 (0.0-0.1) | 0.5 (0.4-0.7) | 0.3 (0.2-0.4) | 0.2 (0.1-0.4) | 0.2 (0.1-0.4) |
| **Current daily PED** |  |  |  |  |  |  |
| non-use | 0.2 (0.2-0.2) | 0.1 (0.0-0.1) | 0.4 (0.2-0.5) | 0.2 (0.2-0.3) | 0.1 (0.0-0.3) | 0.1 (0.0-0.3) |
| >0.0-4.9 mg | 0.5 (0.3-0.7) | 0.0 (0.0-0.0) | 0.5 (0.3-0.7) | 0.7 (0.1-1.3) | 0.8 (0.0-2.4) | 0.0 (0.0-0.0) |
| 5.0-14.9 mg | 0.6 (0.5-0.8) | 0.4 (0.0-0.8) | 0.7 (0.5-0.9) | 0.7 (0.3-1.1) | 0.0 (0.0-0.0) | 1.1 (0.0-2.4) |
| 15.0-24.9 mg | 0.6 (0.2-1.0) | 0.6 (0.0-1.8) | 0.8 (0.2-1.4) | 0.0 (0.0-0.0) | 0.0 (0.0-0.0) | 0.0 (0.0-0.0) |
| ≥25.0 mg | 1.3 (0.5-2.1) | 0.0 (0.0-0.0) | 1.8 (0.5-3.0) | 0.7 (0.0-2.2) | 8.7 (0.0-19.5) | 2.3 (0.0-6.7) |
| **Cumulative PED** |  |  |  |  |  |  |
| non-use | 0.2 (0.2-0.3) | 0.1 (0.0-0.1) | 0.7 (0.4-1.0) | 0.3 (0.2-0.3) | 0.2 (0.0-0.3) | 0.1 (0.0-0.2) |
| >0.0-959.9 mg | 0.6 (0.4-0.8) | 0.1 (0.0-0.3) | 1.1 (0.7-1.6) | 0.6 (0.2-0.9) | 0.7 (0.0-1.6) | 0.8 (0.0-1.6) |
| 960.0-3,054.9 mg | 0.5 (0.4-0.7) | 0.2 (0.0-0.3) | 0.7 (0.5-0.9) | 0.4 (0.1-0.7) | 0.4 (0.0-1.1) | 1.0 (0.0-2.4) |
| 3,055.0-7,299.9 mg | 0.2 (0.1-0.3) | 0.0 (0.0-0.0) | 0.2 (0.1-0.3) | 0.4 (0.1-0.8) | 0.0 (0.0-0.0) | 0.0 (0.0-0.0) |
| ≥7,300.0 mg | 0.2 (0.0-0.4) | 0.0 (0.0-0.0) | 0.4 (0.0-0.8) | 0.0 (0.0-0.0) | 0.0 (0.0-0.0) | 0.0 (0.0-0.0) |
| **Cumulative probability (95% CI) at 5 years** | 1.8 (1.7-1.9) | 0.5 (0.4-0.6) | 3.0 (2.7-3.3) | 1.9 (1.7-2.1) | 1.1 (0.7-1.4) | 1.2 (0.7-1.6) |
| **Current daily PED** |  |  |  |  |  |  |
| non-use | 1.2 (1.1-1.3) | 0.4 (0.3-0.6) | 1.8 (1.6-2.1) | 1.5 (1.3-1.7) | 1.0 (0.6-1.4) | 0.7 (0.4-1.1) |
| >0.0-4.9 mg | 3.2 (2.6-3.8) | 0.8 (0.0-1.9) | 3.9 (3.1-4.8) | 2.2 (1.1-3.3) | 2.3 (0.0-4.8) | 2.8 (0.0-6.6) |
| 5.0-14.9 mg | 4.3 (3.7-4.8) | 1.2 (0.4-2.0) | 5.5 (4.6-6.4) | 4.2 (3.3-5.1) | 0.8 (0.0-1.8) | 2.8 (0.5-5.1) |
| 15.0-24.9 mg | 4.5 (2.9-6.1) | 1.8 (0.0-4.2) | 6.0 (3.4-8.5) | 2.2 (0.0-4.6) | 0.0 (0.0-0.0) | 10.2 (0.0-20.7) |
| ≥25.0 mg | 7.1 (4.9-9.3) | 1.6 (0.0-3.4) | 11.4 (6.6-15.9) | 8.4 (3.2-13.4) | 5.6 (0.0-12.8) | 6.6 (0.0-15.4) |
| **Cumulative PED** |  |  |  |  |  |  |
| non-use | 1.3 (1.1-1.4) | 0.4 (0.3-0.6) | 2.6 (1.9-3.4) | 1.6 (1.4-1.9) | 1.2 (0.6-1.8) | 0.6 (0.2-1.0) |
| >0.0-959.9 mg | 1.6 (1.3-1.9) | 0.6 (0.2-0.9) | 2.9 (2.1-3.7) | 1.7 (1.1-2.2) | 1.3 (0.1-2.4) | 1.9 (0.6-3.3) |
| 960.0-3,054.9 mg | 2.2 (1.8-2.6) | 0.7 (0.3-1.0) | 3.4 (2.8-4.1) | 1.8 (1.1-2.6) | 1.2 (0.0-2.5) | 2.9 (0.3-5.4) |
| 3,055.0-7,299.9 mg | 2.9 (2.5-3.3) | 0.4 (0.1-0.8) | 3.4 (2.8-3.9) | 4.2 (3.1-5.4) | 1.3 (0.0-3.0) | 2.6 (0.0-5.0) |
| ≥7,300.0 mg | 1.9 (1.6-2.3) | 0.7 (0.1-1.3) | 2.4 (1.9-2.8) | 1.9 (1.3-2.5) | 0.0 (0.0-0.0) | 1.2 (0.0-2.6) |
| **Cumulative probability (95% CI) at 10 years** | 3.7 (3.5-3.9) | 1.0 (0.8-1.3) | 5.9 (5.4-6.3) | 4.1 (3.7-4.4) | 2.3 (1.6-3.0) | 2.2 (1.5-3.0) |
| **Current daily PED** |  |  |  |  |  |  |
| non-use | 2.7 (2.5-2.8) | 0.8 (0.6-1.0) | 4.2 (3.8-4.7) | 3.2 (2.8-3.5) | 2.2 (1.4-2.9) | 1.8 (1.0-2.5) |
| >0.0-4.9 mg | 6.5 (5.5-7.6) | 3.8 (0.8-6.7) | 8.0 (6.4-9.5) | 4.7 (3.0-6.4) | 5.9 (0.1-11.3) | 2.7 (0.0-6.3) |
| 5.0-14.9 mg | 7.8 (6.9-8.6) | 2.4 (0.9-3.9) | 10.1 (8.5-11.6) | 8.2 (6.8-9.6) | 1.1 (0.0-2.4) | 4.6 (1.4-7.6) |
| 15.0-24.9 mg | 9.0 (6.0-11.9) | 5.0 (0.0-11.4) | 13.0 (7.8-17.9) | 3.5 (0.3-6.6) | 4.8 (0.0-13.5) | 11.3 (0.0-22.7) |
| ≥25.0 mg | 9.4 (6.3-12.4) | 4.6 (0.3- 8.8) | 11.0 (6.3-15.4) | 13.6 (6.0-20.5) | 4.1 (0.0-9.4) | 5.2 (0.0-12.1) |
| **Cumulative PED** |  |  |  |  |  |  |
| non-use | 2.6 (2.3-2.9) | 1.0 (0.7-1.3) | 5.0 (3.7-6.2) | 3.3 (2.8-3.8) | 3.0 (1.8-4.2) | 1.6 (0.6-2.5) |
| >0.0-959.9 mg | 3.1 (2.6-3.6) | 0.9 (0.4-1.3) | 5.8 (4.4-7.2) | 3.8 (2.9-4.7) | 1.1 (0.1-2.0) | 2.9 (1.0-4.8) |
| 960.0-3,054.9 mg | 4.0 (3.5-4.6) | 1.3 (0.7-1.8) | 6.7 (5.5-7.8) | 3.7 (2.6-4.9) | 3.3 (0.4-6.2) | 2.3 (0.3-4.3) |
| 3,055.0-7,299.9 mg | 4.6 (4.0-5.1) | 0.9 (0.3-1.4) | 5.5 (4.6-6.4) | 6.7 (5.1-8.3) | 1.1 (0.0-2.6) | 2.6 (0.0-5.1) |
| ≥7,300.0 mg | 4.7 (4.2-5.2) | 1.3 (0.5-2.0) | 5.8 (5.0-6.6) | 4.9 (3.9-5.9) | 1.3 (0.0-2.5) | 3.3 (1.0-5.7) |
| **Peripheral arterial disease events** | **All disease** | **Inflammatory**  **bowel disease** | **PMR and/or GCA** | **Rheumatoid arthritis** | **Systemic lupus erythematosus** | **Vasculitis** |
| Incident CVD, n (%) | 1,227 | 155 | 561 | 400 | 53 | 61 |
| **Cumulative probability (95% CI) at 1 year** | 0.3 (0.3-0.3) | 0.1 (0.1-0.2) | 0.5 (0.4-0.6) | 0.2 (0.1-0.3) | 0.2 (0.1-0.4) | 0.5 (0.2-0.8) |
| **Current daily PED** |  |  |  |  |  |  |
| non-use | 0.2 (0.1-0.2) | 0.1 (0.1-0.2) | 0.3 (0.2-0.4) | 0.2 (0.1-0.2) | 0.2 (0.0-0.4) | 0.4 (0.2-0.6) |
| >0.0-4.9 mg | 0.4 (0.2-0.6) | 0.6 (0.0-1.3) | 0.4 (0.2-0.7) | 0.3 (0.0-0.6) | 0.0 (0.0-0.0) | 1.2 (0.0-3.4) |
| 5.0-14.9 mg | 0.6 (0.4-0.8) | 0.4 (0.0-0.9) | 0.7 (0.5-0.9) | 0.5 (0.2-0.8) | 0.3 (0.0-0.8) | 0.7 (0.0-1.7) |
| 15.0-24.9 mg | 0.9 (0.4-1.3) | 0.6 (0.0-1.9) | 0.8 (0.3-1.4) | 0.6 (0.0-1.8) | 1.6 (0.0-4.7) | 1.6 (0.0-4.5) |
| ≥25.0 mg | 0.7 (0.1-1.3) | 0.0 (0.0-0.0) | 1.3 (0.2-2.4) | 0.0 (0.0-0.0) | 0.0 (0.0-0.0) | 2.1 (0.0-6.2) |
| **Cumulative PED** |  |  |  |  |  |  |
| non-use | 0.2 (0.2-0.3) | 0.1 (0.0-0.2) | 0.4 (0.2-0.6) | 0.2 (0.1-0.3) | 0.2 (0.0-0.4) | 0.5 (0.2-0.8) |
| >0.0-959.9 mg | 0.4 (0.2-0.5) | 0.1 (0.0-0.2) | 0.8 (0.4-1.2) | 0.1 (0.0-0.3) | 0.3 (0.0-1.0) | 0.7 (0.0-1.6) |
| 960.0-3,054.9 mg | 0.6 (0.4-0.7) | 0.2 (0.0-0.4) | 0.8 (0.5-1.0) | 0.4 (0.1-0.8) | 0.0 (0.0-0.0) | 0.9 (0.0-2.2) |
| 3,055.0-7,299.9 mg | 0.3 (0.2-0.4) | 0.4 (0.0-0.9) | 0.3 (0.2-0.4) | 0.4 (0.0-0.7) | 0.3 (0.0-1.0) | 0.0 (0.0-0.0) |
| ≥7,300.0 mg | 0.1 (0.0-0.3) | 0.6 (0.0-1.9) | 0.0 (0.0-0.0) | 0.2 (0.0-0.6) | 0.0 (0.0-0.0) | 0.0 (0.0-0.0) |
| **Cumulative probability (95% CI) at 5 years** | 1.4 (1.2-1.5) | 0.6 (0.4-0.7) | 2.1 (1.9-2.3) | 1.3 (1.1-1.5) | 0.9 (0.6-1.3) | 1.5 (1.0-2.0) |
| **Current daily PED** |  |  |  |  |  |  |
| non-use | 1.0 (0.9-1.1) | 0.5 (0.3-0.6) | 1.3 (1.1-1.6) | 1.1 (0.9-1.2) | 0.8 (0.4-1.2) | 1.1 (0.7-1.6) |
| >0.0-4.9 mg | 2.1 (1.6-2.6) | 1.0 (0.0-2.1) | 2.3 (1.6-2.9) | 1.9 (0.9-3.0) | 1.7 (0.0-3.9) | 3.7 (0.0-7.7) |
| 5.0-14.9 mg | 3.2 (2.7-3.6) | 2.2 (1.0-3.4) | 4.4 (3.5-5.2) | 2.2 (1.5-2.9) | 1.6 (0.2-3.1) | 3.4 (0.9-5.9) |
| 15.0-24.9 mg | 3.4 (2.1-4.7) | 2.9 (0.0-6.1) | 3.9 (2.0-5.8) | 2.0 (0.0-4.3) | 1.7 (0.0-5.0) | 7.5 (0.0-15.3) |
| ≥25.0 mg | 3.3 (1.8-4.8) | 0.0 (0.0-0.0) | 5.8 (2.5-8.9) | 4.8 (0.9-8.6) | 0.0 (0.0-0.0) | 2.1 (0.0-6.2) |
| **Cumulative PED** |  |  |  |  |  |  |
| non-use | 1.1 (0.9-1.2) | 0.7 (0.5-0.9) | 2.2 (1.5-2.9) | 1.1 (0.9-1.3) | 0.7 (0.3-1.2) | 1.3 (0.7-1.9) |
| >0.0-959.9 mg | 1.2 (1.0-1.5) | 0.4 (0.1-0.6) | 2.3 (1.5-3.0) | 1.1 (0.7-1.6) | 1.3 (0.2-2.5) | 2.3 (0.8-3.8) |
| 960.0-3,054.9 mg | 1.8 (1.5-2.1) | 0.4 (0.1-0.7) | 2.7 (2.1-3.2) | 2.3 (1.4-3.1) | 0.4 (0.0-1.3) | 0.9 (0.0-2.1) |
| 3,055.0-7,299.9 mg | 1.9 (1.6-2.2) | 0.6 (0.2-1.1) | 2.2 (1.7-2.6) | 2.2 (1.4-3.0) | 2.6 (0.3-4.8) | 2.6 (0.0-5.1) |
| ≥7,300.0 mg | 1.2 (1.0-1.5) | 0.7 (0.1-1.4) | 1.5 (1.1-1.8) | 1.0 (0.5-1.4) | 0.6 (0.0-1.4) | 1.1 (0.0-2.4) |
| **Cumulative probability (95% CI) at 10 years** | 2.8 (2.6-3.0) | 1.4 (1.1-1.6) | 4.2 (3.8-4.6) | 2.7 (2.4-3.0) | 2.1 (1.4-2.7) | 2.8 (1.9-3.6) |
| **Current daily PED** |  |  |  |  |  |  |
| non-use | 2.0 (1.8-2.2) | 1.1 (0.9-1.4) | 2.7 (2.4-3.1) | 2.2 (1.9-2.5) | 1.6 (1.0-2.3) | 2.2 (1.3-3.0) |
| >0.0-4.9 mg | 5.5 (4.5-6.6) | 3.3 (0.7-5.8) | 5.9 (4.4-7.3) | 5.1 (3.2-7.0) | 8.7 (1.9-14.9) | 3.4 (0.0-7.1) |
| 5.0-14.9 mg | 5.9 (5.1-6.7) | 2.8 (1.4-4.3) | 8.9 (7.3-10.5) | 4.5 (3.4-5.6) | 1.9 (0.3-3.4) | 6.4 (2.2-10.5) |
| 15.0-24.9 mg | 6.9 (4.3-9.4) | 8.3 (0.0-5.9) | 7.8 (3.8-11.5) | 2.6 (0.0-5.3) | 8.0 (0.0-19.2) | 14.8 (0.0-28.7) |
| ≥25.0 mg | 7.0 (4.3-9.6) | 5.5 (0.7-10.0) | 8.0 (3.4-12.3) | 8.6 (2.9-14.0) | 3.4 (0.0- 9.6) | 1.7 (0.0-4.9) |
| **Cumulative PED** |  |  |  |  |  |  |
| non-use | 2.1 (1.9-2.4) | 1.4 (1.0-1.7) | 3.9 (2.8-5.1) | 2.3 (1.9-2.7) | 1.9 (1.0-2.8) | 2.2 (1.2-3.2) |
| >0.0-959.9 mg | 2.4 (2.0-2.9) | 1.0 (0.5-1.5) | 3.8 (2.7-5.0) | 2.8 (2.0-3.5) | 1.5 (0.3-2.7) | 3.5 (1.2-5.8) |
| 960.0-3,054.9 mg | 2.7 (2.2-3.1) | 1.2 (0.6-1.7) | 4.1 (3.2-4.9) | 2.8 (1.8-3.7) | 0.4 (0.0-1.1) | 1.6 (0.0-3.6) |
| 3,055.0-7,299.9 mg | 3.3 (2.8-3.8) | 1.6 (0.8-2.3) | 3.8 (3.0-4.5) | 3.9 (2.6-5.2) | 2.2 (0.3-4.1) | 5.1 (0.6-9.4) |
| ≥7,300.0 mg | 3.7 (3.2-4.1) | 2.0 (1.0-2.9) | 4.5 (3.7-5.2) | 3.0 (2.2-3.8) | 3.5 (1.5-5.4) | 3.1 (0.7-5.5) |
| **Cerebrovascular disease events** | **All disease** | **Inflammatory**  **bowel disease** | **PMR and/or GCA** | **Rheumatoid arthritis** | **Systemic lupus erythematosus** | **Vasculitis** |
| Incident CVD, n (%) | 2,031 | 215 | 1,039 | 648 | 72 | 57 |
| **Cumulative probability (95% CI) at 1 year** | 0.4 (0.4-0.5) | 0.2 (0.1-0.3) | 0.7 (0.6-0.8) | 0.4 (0.3-0.5) | 0.2 (0.1-0.4) | 0.3 (0.1-0.5) |
| **Current daily PED** |  |  |  |  |  |  |
| non-use | 0.3 (0.2-0.3) | 0.1 (0.1-0.2) | 0.5 (0.3-0.7) | 0.3 (0.2-0.4) | 0.2 (0.0-0.4) | 0.2 (0.0-0.4) |
| >0.0-4.9 mg | 0.6 (0.4-0.9) | 0.8 (0.0-1.8) | 0.6 (0.3-0.8) | 1.1 (0.3-1.9) | 0.0 (0.0-0.0) | 0.0 (0.0-0.0) |
| 5.0-14.9 mg | 0.7 (0.6-0.9) | 0.6 (0.1-1.2) | 0.8 (0.6-1.0) | 0.6 (0.3-0.9) | 0.6 (0.0-1.3) | 1.1 (0.0-2.3) |
| 15.0-24.9 mg | 1.1 (0.6-1.7) | 0.0 (0.0-0.0) | 1.5 (0.7-2.2) | 1.1 (0.0-2.6) | 0.0 (0.0-0.0) | 0.0 (0.0-0.0) |
| ≥25.0 mg | 1.3 (0.5-2.1) | 0.9 (0.0-2.0) | 2.2 (0.8-3.7) | 0.0 (0.0-0.0) | 0.0 (0.0-0.0) | 0.0 (0.0-0.0) |
| **Cumulative PED** |  |  |  |  |  |  |
| non-use | 0.3 (0.2-0.4) | 0.2 (0.1-0.2) | 0.8 (0.5-1.1) | 0.3 (0.2-0.4) | 0.2 (0.0-0.3) | 0.2 (0.0-0.4) |
| >0.0-959.9 mg | 0.7 (0.5-0.9) | 0.2 (0.0-0.4) | 1.3 (0.8-1.7) | 0.6 (0.2-0.9) | 0.7 (0.0-1.6) | 0.5 (0.0-1.2) |
| 960.0-3,054.9 mg | 0.8 (0.6-0.9) | 0.3 (0.0-0.5) | 1.0 (0.7-1.2) | 0.7 (0.3-1.1) | 0.4 (0.0-1.2) | 0.5 (0.0-1.5) |
| 3,055.0-7,299.9 mg | 0.3 (0.2-0.4) | 0.4 (0.0-0.8) | 0.2 (0.1-0.4) | 0.4 (0.0-0.7) | 0.3 (0.0-1.0) | 0.0 (0.0-0.0) |
| ≥7,300.0 mg | 0.4 (0.1-0.8) | 0.0 (0.0-0.0) | 0.5 (0.0-1.0) | 0.4 (0.0-0.9) | 0.0 (0.0-0.0) | 1.4 (0.0-4.2) |
| **Cumulative probability (95% CI) at 5 years** | 2.1 (2.0-2.3) | 0.8 (0.7-1.0) | 3.6 (3.3-3.9) | 2.0 (1.7-2.2) | 1.4 (1.0-1.9) | 1.4 (0.9-1.8) |
| **Current daily PED** |  |  |  |  |  |  |
| non-use | 1.6 (1.4-1.7) | 0.6 (0.5-0.7) | 2.6 (2.3-3.0) | 1.6 (1.4-1.8) | 1.3 (0.8-1.8) | 1.1 (0.7-1.6) |
| >0.0-4.9 mg | 4.0 (3.3-4.7) | 2.3 (0.4-4.1) | 4.3 (3.4-5.2) | 4.4 (2.9-6.0) | 0.9 (0.0-2.6) | 2.4 (0.0-5.7) |
| 5.0-14.9 mg | 4.3 (3.8-4.9) | 2.3 (1.1-3.4) | 6.0 (5.0-6.9) | 3.1 (2.3-3.9) | 2.4 (0.7-4.1) | 3.7 (0.8-6.6) |
| 15.0-24.9 mg | 4.2 (2.7-5.6) | 7.4 (1.9-12.6) | 4.7 (2.6-6.7) | 2.5 (0.0-4.9) | 0.0 (0.0-0.0) | 0.0 (0.0-0.0) |
| ≥25.0 mg | 5.7 (3.7-7.7) | 3.3 (0.8-5.7) | 11.6 (6.5-16.5) | 2.9 (0.0-5.6) | 0.0 (0.0-0.0) | 0.0 (0.0-0.0) |
| **Cumulative PED** |  |  |  |  |  |  |
| non-use | 1.6 (1.4-1.7) | 0.9 (0.6-1.1) | 3.3 (2.5-4.1) | 1.8 (1.5-2.1) | 1.3 (0.7-1.8) | 1.3 (0.7-1.9) |
| >0.0-959.9 mg | 2.1 (1.7-2.4) | 0.6 (0.3-1.0) | 4.6 (3.6-5.7) | 1.8 (1.2-2.4) | 1.6 (0.3-3.0) | 1.3 (0.1-2.4) |
| 960.0-3,054.9 mg | 3.0 (2.6-3.4) | 0.8 (0.4-1.2) | 4.6 (3.8-5.3) | 3.1 (2.1-4.0) | 2.9 (0.5-5.2) | 1.7 (0.0-3.6) |
| 3,055.0-7,299.9 mg | 3.2 (2.8-3.7) | 0.6 (0.2-1.1) | 4.2 (3.6-4.8) | 3.0 (2.0-4.0) | 2.1 (0.0-4.2) | 0.5 (0.0-1.5) |
| ≥7,300.0 mg | 1.8 (1.5-2.1) | 1.3 (0.5-2.2) | 2.1 (1.7-2.5) | 1.4 (0.9-2.0) | 0.6 (0.0-1.3) | 2.0 (0.2-3.8) |
| **Cumulative probability (95% CI) at 10 years** | 4.6 (4.4-4.8) | 1.9 (1.6-2.1) | 7.7 (7.1-8.2) | 4.5 (4.1-4.9) | 2.5 (1.8-3.2) | 3.0 (2.1-3.9) |
| **Current daily PED** |  |  |  |  |  |  |
| non-use | 3.8 (3.5-4.0) | 1.6 (1.3-1.9) | 6.4 (5.9-7.0) | 3.8 (3.4-4.2) | 2.3 (1.6-3.1) | 2.6 (1.7-3.5) |
| >0.0-4.9 mg | 7.4 (6.3-8.6) | 2.5 (0.5-4.5) | 8.7 (7.0-10.3) | 7.4 (5.2-9.5) | 2.6 (0.0-6.3) | 4.3 (0.0-9.2) |
| 5.0-14.9 mg | 8.1 (7.2-9.0) | 3.9 (1.9-5.8) | 11.1 (9.5-12.7) | 6.8 (5.5-8.1) | 4.1 (1.8-6.3) | 6.4 (1.8-10.6) |
| 15.0-24.9 mg | 6.4 (4.1-8.6) | 10.6 (3.4-17.1) | 7.1 (3.7-10.4) | 4.4 (0.4-8.1) | 0.0 (0.0-0.0) | 0.0 (0.0-0.0) |
| ≥25.0 mg | 9.8 (6.9-12.6) | 5.7 (2.0-9.2) | 19.4 (11.3-26.5) | 8.0 (2.7-12.8) | 0.0 (0.0-0.0) | 6.2 (0.0-17.2) |
| **Cumulative PED** |  |  |  |  |  |  |
| non-use | 3.3 (3.0-3.6) | 1.7 (1.3-2.1) | 6.0 (4.6-7.3) | 4.1 (3.5-4.6) | 2.5 (1.5-3.4) | 2.7 (1.6-3.7) |
| >0.0-959.9 mg | 4.5 (3.9-5.1) | 2.2 (1.4-3.0) | 9.1 (7.3-10.9) | 4.5 (3.5-5.4) | 2.4 (0.8-4.0) | 3.3 (1.1-5.5) |
| 960.0-3,054.9 mg | 5.3 (4.7-5.9) | 1.6 (0.9-2.2) | 9.7 (8.3-11.1) | 4.0 (2.8-5.1) | 3.1 (0.7-5.3) | 1.4 (0.0-2.9) |
| 3,055.0-7,299.9 mg | 6.5 (5.8-7.2) | 1.8 (1.0-2.6) | 9.0 (7.8-10.1) | 5.9 (4.3-7.5) | 3.5 (0.5-6.5) | 4.3 (0.0-8.5) |
| ≥7,300.0 mg | 4.7 (4.2-5.2) | 2.3 (1.3-3.2) | 5.6 (4.8-6.4) | 4.7 (3.8-5.7) | 1.9 (0.5-3.2) | 3.9 (1.2-6.5) |
| **Abdominal aortic aneurysm events** | **All disease** | **Inflammatory**  **bowel disease** | **PMR and/or GCA** | **Rheumatoid arthritis** | **Systemic lupus erythematosus** | **Vasculitis** |
| Incident CVD, n (%) | 308 | 24 | 179 | 89 | 3 | 13 |
| **Cumulative probability (95% CI) at 1 year** | 0.1 (0.0-0.1) | 0.0 (0.0-0.0) | 0.1 (0.1-0.1) | 0.1 (0.0-0.1) | 0.0 (0.0-0.0) | 0.1 (0.0-0.2) |
| **Current daily PED** |  |  |  |  |  |  |
| non-use | 0.0 (0.0-0.1) | 0.0 (0.0-0.0) | 0.1 (0.0-0.1) | 0.1 (0.0-0.1) | 0.0 (0.0-0.0) | 0.0 (0.0-0.1) |
| >0.0-4.9 mg | 0.1 (0.0-0.2) | 0.0 (0.0-0.0) | 0.1 (0.0-0.2) | 0.1 (0.0-0.4) | 0.0 (0.0-0.0) | 2.2 (0.0-5.1) |
| 5.0-14.9 mg | 0.1 (0.1-0.2) | 0.0 (0.0-0.0) | 0.2 (0.1-0.3) | 0.1 (0.0-0.3) | 0.0 (0.0-0.0) | 0.0 (0.0-0.0) |
| 15.0-24.9 mg | 0.0 (0.0-0.0) | 0.0 (0.0-0.0) | 0.0 (0.0-0.0) | 0.0 (0.0-0.0) | 0.0 (0.0-0.0) | 0.0 (0.0-0.0) |
| ≥25.0 mg | 0.1 (0.0-0.3) | 0.0 (0.0-0.0) | 0.2 (0.0-0.7) | 0.0 (0.0-0.0) | 0.0 (0.0-0.0) | 0.0 (0.0-0.0) |
| **Cumulative PED** |  |  |  |  |  |  |
| non-use | 0.0 (0.0-0.1) | 0.0 (0.0-0.0) | 0.1 (0.0-0.2) | 0.1 (0.0-0.1) | 0.0 (0.0-0.0) | 0.1 (0.0-0.2) |
| >0.0-959.9 mg | 0.1 (0.0-0.2) | 0.0 (0.0-0.0) | 0.2 (0.0-0.4) | 0.1 (0.0-0.3) | 0.0 (0.0-0.0) | 0.0 (0.0-0.0) |
| 960.0-3,054.9 mg | 0.1 (0.0-0.2) | 0.0 (0.0-0.0) | 0.2 (0.1-0.3) | 0.0 (0.0-0.0) | 0.0 (0.0-0.0) | 0.9 (0.0-2.2) |
| 3,055.0-7,299.9 mg | 0.0 (0.0-0.1) | 0.0 (0.0-0.0) | 0.0 (0.0-0.1) | 0.1 (0.0-0.3) | 0.0 (0.0-0.0) | 0.0 (0.0-0.0) |
| ≥7,300.0 mg | 0.0 (0.0-0.0) | 0.0 (0.0-0.0) | 0.0 (0.0-0.0) | 0.0 (0.0-0.0) | 0.0 (0.0-0.0) | 0.0 (0.0-0.0) |
| **Cumulative probability (95% CI) at 5 years** | 0.2 (0.2-0.3) | 0.0 (0.0-0.1) | 0.5 (0.3-0.6) | 0.2 (0.2-0.3) | 0.0 (0.0-0.1) | 0.3 (0.1-0.6) |
| **Current daily PED** |  |  |  |  |  |  |
| non-use | 0.1 (0.1-0.2) | 0.0 (0.0-0.1) | 0.2 (0.1-0.3) | 0.2 (0.1-0.2) | 0.0 (0.0-0.1) | 0.3 (0.0-0.5) |
| >0.0-4.9 mg | 0.8 (0.5-1.1) | 0.0 (0.0-0.0) | 1.0 (0.5-1.4) | 0.4 (0.0-0.9) | 0.0 (0.0-0.0) | 1.8 (0.0-4.2) |
| 5.0-14.9 mg | 0.7 (0.5-0.9) | 0.0 (0.0-0.0) | 1.0 (0.6-1.3) | 0.6 (0.3-1.0) | 0.0 (0.0-0.0) | 0.6 (0.0-1.8) |
| 15.0-24.9 mg | 0.4 (0.0-0.9) | 0.0 (0.0-0.0) | 0.7 (0.0-1.8) | 0.0 (0.0-0.0) | 0.0 (0.0-0.0) | 0.0 (0.0-0.0) |
| ≥25.0 mg | 0.2 (0.0-0.6) | 0.0 (0.0-0.0) | 0.7 (0.0-1.7) | 0.0 (0.0-0.0) | 0.0 (0.0-0.0) | 0.0 (0.0-0.0) |
| **Cumulative PED** |  |  |  |  |  |  |
| non-use | 0.2 (0.1-0.2) | 0.1 (0.0-0.1) | 0.3 (0.0-0.5) | 0.2 (0.1-0.3) | 0.1 (0.0-0.2) | 0.3 (0.0-0.6) |
| >0.0-959.9 mg | 0.2 (0.1-0.3) | 0.0 (0.0-0.0) | 0.5 (0.1-0.8) | 0.1 (0.0-0.2) | 0.0 (0.0-0.0) | 0.3 (0.0-1.0) |
| 960.0-3,054.9 mg | 0.3 (0.2-0.5) | 0.0 (0.0-0.0) | 0.6 (0.3-0.9) | 0.2 (0.0-0.4) | 0.0 (0.0-0.0) | 0.9 (0.0-2.2) |
| 3,055.0-7,299.9 mg | 0.4 (0.3-0.6) | 0.0 (0.0-0.0) | 0.5 (0.3-0.7) | 0.6 (0.2-1.1) | 0.0 (0.0-0.0) | 0.0 (0.0-0.0) |
| ≥7,300.0 mg | 0.3 (0.2-0.4) | 0.0 (0.0-0.0) | 0.4 (0.2-0.5) | 0.2 (0.0-0.4) | 0.0 (0.0-0.0) | 0.4 (0.0-1.1) |
| **Cumulative probability (95% CI) at 10 years** | 0.7 (0.6-0.8) | 0.2 (0.1-0.4) | 1.4 (1.1-1.6) | 0.5 (0.4-0.7) | 0.1 (0.0-0.2) | 0.5 (0.2-0.9) |
| **Current daily PED** |  |  |  |  |  |  |
| non-use | 0.5 (0.4-0.6) | 0.2 (0.1-0.4) | 1.0 (0.7-1.2) | 0.3 (0.2-0.5) | 0.0 (0.0-0.1) | 0.5 (0.1-0.9) |
| >0.0-4.9 mg | 1.9 (1.3-2.5) | 0.0 (0.0-0.0) | 2.5 (1.5-3.5) | 1.6 (0.5-2.6) | 0.0 (0.0-0.0) | 1.6 (0.0-3.8) |
| 5.0-14.9 mg | 1.5 (1.1-1.9) | 0.6 (0.0-1.5) | 2.5 (1.6-3.4) | 1.1 (0.6-1.6) | 0.5 (0.0-1.5) | 0.5 (0.0-1.5) |
| 15.0-24.9 mg | 0.6 (0.0-1.4) | 0.0 (0.0-0.0) | 0.6 (0.0-1.5) | 1.1 (0.0-3.3) | 0.0 (0.0-0.0) | 0.0 (0.0-0.0) |
| ≥25.0 mg | 0.9 (0.0-2.0) | 0.0 (0.0-0.0) | 2.2 (0.0-5.2) | 1.3 (0.0-3.8) | 0.0 (0.0-0.0) | 0.0 (0.0-0.0) |
| **Cumulative PED** |  |  |  |  |  |  |
| non-use | 0.4 (0.3-0.6) | 0.4 (0.1-0.6) | 1.4 (0.6-2.2) | 0.4 (0.2-0.5) | 0.1 (0.0-0.2) | 0.4 (0.0-0.9) |
| >0.0-959.9 mg | 0.6 (0.3-0.8) | 0.4 (0.0-0.7) | 1.7 (0.7-2.6) | 0.2 (0.0-0.5) | 0.0 (0.0-0.0) | 0.9 (0.0-2.1) |
| 960.0-3,054.9 mg | 0.6 (0.4-0.8) | 0.0 (0.0-0.0) | 1.2 (0.7-1.7) | 0.4 (0.0-0.8) | 0.0 (0.0-0.0) | 0.8 (0.0-1.8) |
| 3,055.0-7,299.9 mg | 1.0 (0.7-1.3) | 0.2 (0.0-0.5) | 1.3 (0.8-1.8) | 1.2 (0.5-2.0) | 0.0 (0.0-0.0) | 0.0 (0.0-0.0) |
| ≥7,300.0 mg | 1.0 (0.7-1.2) | 0.1 (0.0-0.3) | 1.4 (1.0-1.8) | 0.8 (0.4-1.2) | 0.3 (0.0-0.9) | 0.3 (0.0-0.9) |

Note: CI, confidence interval; CVD, cardiovascular disease; GCA, giant cell arteritis; IQR, interquartile range; PED, prednisolone-equivalent dose; PMR, polymyalgia rheumatica

**Table H. Associations between time variant oral glucocorticoid prednisolone-equivalent dose and incident all-cause cardiovascular disease by immune-mediated inflammatory disease, reported as crude hazard ratios with 95% CI**

|  | **Crude hazard ratios with 95% CI** | | | | | |
| --- | --- | --- | --- | --- | --- | --- |
|  | **All diseases*** | **PMR and/or GCA** | **Inflammatory bowel disease** | **Rheumatoid arthritis** | **Systemic lupus erythematosus** | **Vasculitis** |
| **No. of events** | 13,426 | 6,267 | 1,937 | 4,236 | 375 | 611 |
| **Ever use** (ref: non-use since 1 year prior to follow-up start) | 2.13 (2.05-2.21) | 1.50 (1.38- 1.63) | 1.27 (1.16-1.40) | 1.90 (1.79-2.02) | 1.60 (1.30-1.97) | 1.83 (1.55-2.15) |
| **Current use** (ref: non-use) | 2.88 (2.77-2.98) | 1.81 (1.72- 1.91) | 3.23 (2.91-3.59) | 2.66 (2.49-2.83) | 2.43 (1.95-3.01) | 2.49 (2.09-2.96) |
| **Current daily dose per 5 mg/day** | 1.47 (1.45-1.49) | 1.17 (1.15- 1.20) | 1.42 (1.40-1.44) | 1.32 (1.30-1.35) | 1.28 (1.20-1.37) | 1.23 (1.17-1.29) |
| **Current daily dose category** (ref: non-use) | 1.00 | 1.00 | 1.00 | 1.00 | 1.00 | 1.00 |
| >0.0-4.9 mg | 2.66 (2.49-2.84) | 1.63 (1.49- 1.78) | 2.52 (1.94-3.28) | 2.30 (2.03-2.61) | 2.83 (1.95-4.10) | 2.22 (1.58-3.13) |
| 5.0-14.9 mg | 2.78 (2.66-2.91) | 1.83 (1.72- 1.96) | 2.90 (2.46-3.43) | 2.53 (2.35-2.73) | 2.04 (1.55-2.70) | 2.36 (1.87-2.99) |
| 15.0-24.9 mg | 3.31 (2.96-3.71) | 2.08 (1.81- 2.40) | 4.00 (2.89-5.53) | 3.30 (2.61-4.17) | 2.23 (0.97-5.09) | 2.86 (1.78-4.60) |
| ≥25 mg | 4.33 (3.91-4.80) | 2.82 (2.35- 3.37) | 5.12 (4.01-6.55) | 6.05 (5.01-7.29) | 5.80 (3.37-10.00) | 3.62 (2.27-5.79) |
| **Cumulative dose per 1000 mg** | 1.01 (1.01-1.01) | 1.02 (1.02- 1.02) | 1.01 (1.00-1.01) | 1.03 (1.02-1.03) | 1.02 (1.01-1.03) | 1.03 (1.02-1.04) |
| **Cumulative dose category** (ref: non-use) | 1.00 | 1.00 | 1.00 | 1.00 | 1.00 | 1.00 |
| >0.0-959.9 mg | 1.65 (1.56-1.74) | 1.38 (1.24- 1.54) | 1.22 (1.07-1.39) | 1.54 (1.41-1.69) | 1.50 (1.11-2.03) | 1.78 (1.42-2.22) |
| 960-3054.9 mg | 1.87 (1.78-1.97) | 1.38 (1.25- 1.52) | 1.14 (1.00-1.30) | 1.78 (1.61-1.98) | 1.42 (0.97-2.07) | 1.41 (1.03-1.93) |
| 3055-7299.9 mg | 2.32 (2.21-2.44) | 1.45 (1.32- 1.59) | 1.28 (1.10-1.49) | 2.20 (1.99-2.42) | 1.64 (1.14-2.35) | 1.60 (1.20-2.12) |
| ≥7300 mg | 2.81 (2.67-2.95) | 1.78 (1.62- 1.96) | 1.68 (1.45-1.96) | 2.23 (2.04-2.42) | 1.80 (1.35-2.39) | 2.43 (1.94-3.05) |

Note: CI, confidence interval; Unadjusted hazard ratios from Cox proportional imputed models.

**Table I. Association between time variant oral glucocorticoid dose and incident cardiovascular disease in patients with 6 immune-mediated inflammatory diseases from complete case analysis**

|  | **Adjusted hazard ratios with 95% CI** | | | | | |  |
| --- | --- | --- | --- | --- | --- | --- | --- |
|  | **All CVD** | **Atrial fibrillation** | **Heart failure** | **Myocardial infarction** | **Peripheral arterial disease** | **Cerebrovascular disease** | **Abdominal aortic aneurysm** |
| **No. of events** | 768 | 229 | 162 | 102 | 90 | 113 | 72 |
| **Ever use** (ref: non-use since 1 year prior to follow-up start) | 1.67 (1.30-2.15) | 1.18 (0.75-1.86) | 1.92 (0.99-3.72) | 2.45 (1.30-4.59) | 3.98 (1.60-9.90) | 0.98 (0.48-2.00) | 1.22 (0.36-4.10) |
| **Current use** (ref: non-use) | 2.01 (1.67-2.42) | 2.08 (1.51-2.88) | 2.25 (1.52-3.33) | 1.83 (1.22-2.75) | 3.37 (1.93-5.87) | 0.97 (0.56-1.69) | 2.32 (0.92-5.84) |
| **Current daily dose per 5 mg/day** | 1.21 (1.14-1.28) | 1.19 (1.08-1.31) | 1.25 (1.12-1.41) | 1.17 (1.02-1.34) | 1.34 (1.16-1.53) | 0.93 (0.70-1.23) | 1.31 (1.03-1.67) |
| **Current daily dose category** (ref: non-use) | 1.00 | 1.00 | 1.00 | 1.00 | 1.00 | 1.00 | 1.00 |
| >0.0-4.9 mg | 1.75 (1.35-2.28) | 1.83 (1.10-3.06) | 2.20 (1.19-4.07) | 1.66 (0.87-3.16) | 2.96 (1.14-7.67) | 0.91 (0.36-2.28) | 1.09 (0.16-7.42) |
| 5.0-14.9 mg | 1.94 (1.54-2.45) | 2.00 (1.29-3.10) | 1.98 (1.17-3.36) | 1.81 (1.11-2.95) | 2.56 (1.26-5.22) | 1.03 (0.48-2.19) | 2.57 (0.84-7.80) |
| 15.0-24.9 mg | 2.08 (1.17-3.71) | 2.54 (1.22-5.31) | 2.25 (0.63-8.02) | 1.93 (0.75-5.02) | 3.18 (0.61-16.70) | 0.79 (0.08-7.59) | - |
| ≥25 mg | 4.44 (2.88-6.86) | 3.71 (1.51-9.10) | 5.18 (2.06-12.99) | 3.11 (0.97-9.94) | 12.89 (5.39-30.84) | - | 8.31 (1.58-43.63) |
| **Cumulative dose per 1000 mg** | 1.03 (1.01-1.05) | 1.04 (1.01-1.07) | 1.06 (1.03-1.09) | 1.03 (0.99-1.07) | 1.01 (0.96-1.07) | 0.94 (0.88-1.01) | 1.03 (0.95-1.12) |
| **Cumulative dose category** (ref: non-use) | 1.00 | 1.00 | 1.00 | 1.00 | 1.00 | 1.00 | 1.00 |
| >0.0-959.9 mg | 1.74 (1.31-2.33) | 1.28 (0.76-2.17) | 2.07 (0.98-4.34) | 2.45 (1.20-5.00) | 4.31 (1.60-11.62) | 1.08 (0.47-2.50) | 1.02 (0.22-4.73) |
| 960-3054.9 mg | 1.64 (1.22-2.19) | 1.10 (0.65-1.86) | 1.41 (0.65-3.05) | 2.31 (1.12-4.75) | 4.11 (1.52-11.12) | 0.95 (0.41-2.18) | 1.03 (0.25-4.23) |
| 3055-7299.9 mg | 1.49 (1.09-2.03) | 1.08 (0.62-1.88) | 1.84 (0.86-3.95) | 2.36 (1.13-4.94) | 2.83 (0.96- 8.33) | 1.11 (0.49-2.54) | 2.18 (0.48-9.88) |
| ≥7300 mg | 1.97 (1.41-2.76) | 1.39 (0.76-2.54) | 3.77 (1.73-8.20) | 3.05 (1.40-6.63) | 4.46 (1.50-13.25) | 0.41 (0.13-1.25) | 2.11 (0.37-11.93) |

Note: CI, confidence interval; CVD, cardiovascular disease; Hazard ratios from Cox proportional models adjusted for baseline age, sex, index of multiple deprivation, smoking status, ethnicity, body mass index, type of immune-mediated inflammatory disease, comorbidities (diabetes, diagnosed hypertension, cancer, asthma, chronic obstructive pulmonary disease, and renal disease), biomarkers (systolic blood pressure, total cholesterol, high-density lipoprotein cholesterol, low-density lipoprotein cholesterol, c-reactive protein, creatinine), number of hospital admissions in last year, and prescribed non-oral glucocorticoids; and time-varying use of disease-modifying anti-rheumatic drugs and non-steroidal anti-inflammatory drugs; the practice identifier was included as a random intercept to account for clustering effect.

**Table J. Association between time variant oral glucocorticoid dose and incident all-cause cardiovascular disease by type of immune-mediated inflammatory disease from analysis in which missing covariate values were coded as a separate category**

|  | **Adjusted hazard ratios with 95% CI** | | | | | |
| --- | --- | --- | --- | --- | --- | --- |
|  | **All diseases*** | **PMR and/or GCA** | **Inflammatory bowel disease** | **Rheumatoid arthritis** | **Systemic lupus erythematosus** | **Vasculitis** |
| **Ever** use (ref: non-use since 1 year prior to follow-up start) | 1.46 (1.40-1.52) | 1.29 (1.18-1.40) | 1.36 (1.23-1.49) | 1.60 (1.51-1.71) | 1.68 (1.34-2.10) | 1.45 (1.22-1.72) |
| **Current use** (ref: non-use) | 1.92 (1.85-2.00) | 1.68 (1.59-1.77) | 2.67 (2.40-2.98) | 2.06 (1.93-2.19) | 2.53 (2.00-3.21) | 1.95 (1.62-2.35) |
| **Current daily dose per 5 mg/day** | 1.09 (1.07-1.11) | 1.17 (1.15-1.19) | 1.08 (1.06-1.09) | 1.27 (1.25-1.30) | 1.28 (1.20-1.37) | 1.18 (1.12-1.24) |
| **Current daily dose category** (ref: non-use) | 1.00 | 1.00 | 1.00 | 1.00 | 1.00 | 1.00 |
| >0.0-4.9 mg | 1.67 (1.55-1.79) | 1.50 (1.37-1.64) | 2.12 (1.63-2.75) | 1.80 (1.59-2.05) | 2.88 (1.95-4.25) | 1.83 (1.28-2.60) |
| 5.0-14.9 mg | 1.87 (1.78-1.95) | 1.69 (1.58-1.81) | 2.36 (2.00-2.79) | 1.94 (1.80-2.10) | 2.14 (1.59-2.88) | 1.80 (1.40-2.31) |
| 15.0-24.9 mg | 2.36 (2.11-2.65) | 2.07 (1.80-2.38) | 3.38 (2.45-4.65) | 2.70 (2.14-3.40) | 2.57 (1.12-5.90) | 2.33 (1.45-3.76) |
| ≥25 mg | 3.59 (3.24-3.99) | 2.74 (2.29-3.29) | 4.30 (3.38-5.47) | 4.88 (4.04-5.89) | 5.51 (3.15-9.63) | 2.87 (1.77-4.64) |
| **Cumulative dose per 1000 mg** | 1.01 (1.01-1.01) | 1.02 (1.01-1.02) | 1.01 (1.01-1.01) | 1.02 (1.02-1.02) | 1.03 (1.01-1.04) | 1.02 (1.01-1.03) |
| **Cumulative dose category** (ref: non-use) | 1.00 | 1.00 | 1.00 | 1.00 | 1.00 | 1.00 |
| >0.0-959.9 mg | 1.36 (1.28-1.44) | 1.25 (1.12-1.39) | 1.18 (1.04-1.35) | 1.46 (1.33-1.60) | 1.62 (1.19-2.22) | 1.52 (1.21-1.90) |
| 960-3054.9 mg | 1.35 (1.27-1.42) | 1.17 (1.07-1.29) | 1.32 (1.15-1.51) | 1.52 (1.37-1.69) | 1.38 (0.94-2.04) | 1.27 (0.93-1.76) |
| 3055-7299.9 mg | 1.44 (1.36-1.52) | 1.25 (1.14-1.38) | 1.43 (1.22-1.66) | 1.69 (1.53-1.87) | 1.62 (1.11-2.37) | 1.16 (0.86-1.55) |
| ≥7300 mg | 1.72 (1.63-1.82) | 1.52 (1.38-1.68) | 1.80 (1.54-2.10) | 1.74 (1.60-1.90) | 2.04 (1.49-2.78) | 1.74 (1.36-2.24) |

Note: CI, confidence interval; GCA, giant cell arteritis; Hazard ratios from Cox proportional models adjusted for baseline age, sex, index of multiple deprivation, smoking status, ethnicity, body mass index, comorbidities (diabetes, diagnosed hypertension, cancer, asthma, chronic obstructive pulmonary disease, and renal disease), quintiles of biomarkers (systolic blood pressure, total cholesterol, high-density lipoprotein cholesterol, low-density lipoprotein cholesterol, c-reactive protein, creatinine), number of hospital admissions in last year, and prescribed non-oral glucocorticoids; and time-varying use of disease-modifying anti-rheumatic drugs and non-steroidal anti-inflammatory drugs; the practice identifier was included as a random intercept to account for clustering effect. *These estimates were additionally adjusted for the type of immune-mediated inflammatory disease diagnosed. PMR, polymyalgia rheumatica.

**Table K. Association between time variant oral glucocorticoid dose and incident all-cause cardiovascular disease by type of immune-mediated inflammatory disease from analysis in which biomarkers with over 60% missing data were excluded.**

|  | **Adjusted hazard ratios with 95% CI** | | | | | |
| --- | --- | --- | --- | --- | --- | --- |
|  | **All diseases*** | **PMR and/or GCA** | **Inflammatory bowel disease** | **Rheumatoid arthritis** | **Systemic lupus erythematosus** | **Vasculitis** |
| **Ever** use (ref: non-use since 1 year prior to study entry) | 1.45 (1.39-1.51) | 1.26 (1.16-1.37) | 1.36 (1.24-1.50) | 1.61 (1.51-1.71) | 1.65 (1.32-2.06) | 1.43 (1.20-1.69) |
| **Current use** (ref: non-use) | 1.92 (1.85-2.00) | 1.68 (1.59-1.77) | 2.70 (2.43-3.01) | 2.07 (1.94-2.21) | 2.47 (1.95-3.13) | 1.92 (1.60-2.32) |
| **Current daily dose per 5 mg/day** | 1.09 (1.07-1.11) | 1.17 (1.15-1.19) | 1.08 (1.06-1.09) | 1.28 (1.25-1.30) | 1.27 (1.20-1.36) | 1.17 (1.11-1.24) |
| **Current daily dose category** (ref: no-use) | 1.00 | 1.00 | 1.00 | 1.00 | 1.00 | 1.00 |
| >0.0-4.9 mg | 1.67 (1.55-1.79) | 1.49 (1.37-1.63) | 2.15 (1.65-2.79) | 1.81 (1.59-2.05) | 2.68 (1.81-3.96) | 1.79 (1.26-2.54) |
| 5.0-14.9 mg | 1.87 (1.78-1.95) | 1.69 (1.58-1.80) | 2.39 (2.02-2.82) | 1.96 (1.82-2.11) | 2.11 (1.57-2.84) | 1.79 (1.40-2.28) |
| 15.0-24.9 mg | 2.35 (2.10-2.64) | 2.06 (1.79-2.36) | 3.42 (2.48-4.71) | 2.71 (2.15-3.42) | 2.57 (1.12-5.89) | 2.29 (1.43-3.68) |
| ≥25 mg | 3.58 (3.22-3.97) | 2.72 (2.27-3.26) | 4.35 (3.42-5.53) | 4.86 (4.03-5.87) | 5.38 (3.09-9.39) | 2.79 (1.73-4.50) |
| **Cumulative dose per 1000 mg** | 1.01 (1.01-1.01) | 1.02 (1.01-1.02) | 1.01 (1.01-1.01) | 1.02 (1.02-1.03) | 1.03 (1.01-1.04) | 1.02 (1.01-1.03) |
| **Cumulative dose category** (ref: non-use) | 1.00 | 1.00 | 1.00 | 1.00 | 1.00 | 1.00 |
| >0.0-959.9 mg | 1.36 (1.28-1.44) | 1.24 (1.11-1.38) | 1.19 (1.04-1.35) | 1.45 (1.32-1.59) | 1.61 (1.18-2.20) | 1.49 (1.19-1.87) |
| 960-3054.9 mg | 1.33 (1.26-1.41) | 1.15 (1.04-1.27) | 1.32 (1.15-1.51) | 1.51 (1.36-1.68) | 1.36 (0.92-1.99) | 1.27 (0.92-1.74) |
| 3055-7299.9 mg | 1.43 (1.36-1.51) | 1.22 (1.11-1.34) | 1.44 (1.23-1.68) | 1.70 (1.54-1.88) | 1.58 (1.08-2.30) | 1.14 (0.85-1.53) |
| ≥7300 mg | 1.72 (1.63-1.81) | 1.49 (1.35-1.64) | 1.83 (1.57-2.14) | 1.75 (1.61-1.91) | 2.01 (1.47-2.73) | 1.72 (1.34-2.20) |

Note: CI, confidence interval; GCA, giant cell arteritis; Hazard ratios from Cox proportional models adjusted for baseline age, sex, index of multiple deprivation, smoking status, ethnicity, body mass index, comorbidities (diabetes, diagnosed hypertension, cancer, asthma, chronic obstructive pulmonary disease, and renal disease), biomarkers (systolic blood pressure, creatinine), number of hospital admissions in last year, and prescribed non-oral glucocorticoids; and time-varying use of disease-modifying anti-rheumatic drugs and non-steroidal anti-inflammatory drugs; the practice identifier was included as a random intercept to account for clustering effect. *These estimates were additionally adjusted for the type of immune-mediated inflammatory disease diagnosed. PMR, polymyalgia rheumatica.

**Table L. Association between time variant oral glucocorticoid dose and 6 incident cardiovascular disease in patients with 6 immune-mediated inflammatory diseases from analysis in which missing covariate values were coded as a separate category**

|  | **Adjusted hazard ratios with 95% CI** | | | | | |  | |  |
| --- | --- | --- | --- | --- | --- | --- | --- | --- | --- |
|  | **All CVD** | **Atrial fibrillation** | **Heart failure** | **Myocardial infarction** | **Peripheral arterial disease** | **Cerebrovascular disease** | | **Abdominal aortic aneurysm** | |
| **Ever use** (ref: non-use since 1 year prior to study entry) | 1.46 (1.40-1.52) | 1.37 (1.29-1.47) | 1.60 (1.48-1.72) | 1.53 (1.40-1.68) | 1.46 (1.31-1.63) | 1.37 (1.25-1.50) | | 1.34 (1.11-1.63) | |
| **Current use** (ref = non-use) | 1.92 (1.85-2.00) | 1.92 (1.82-2.03) | 2.18 (2.05-2.32) | 2.05 (1.89-2.22) | 1.98 (1.80-2.18) | 1.44 (1.33-1.56) | | 1.81 (1.54-2.12) | |
| **Current daily dose per 5 mg/day** | 1.09 (1.07-1.11) | 1.09 (1.07-1.11) | 1.10 (1.08-1.12) | 1.08 (1.06-1.10) | 1.08 (1.06-1.10) | 1.08 (1.06-1.10) | | 1.09 (1.06-1.12) | |
| **Current daily dose category** (ref: non-use) | 1.00 | 1.00 | 1.00 | 1.00 | 1.00 | 1.00 | | 1.00 | |
| >0.0-4.9 mg | 1.67 (1.55-1.79) | 1.68 (1.53-1.84) | 1.73 (1.54-1.95) | 1.73 (1.48-2.02) | 1.76 (1.51-2.05) | 1.30 (1.14-1.48) | | 1.90 (1.45-2.49) | |
| 5.0-14.9 mg | 1.87 (1.78-1.95) | 1.86 (1.73-2.00) | 2.11 (1.96-2.27) | 2.01 (1.82-2.23) | 1.94 (1.71-2.19) | 1.44 (1.30-1.58) | | 1.74 (1.42-2.13) | |
| 15.0-24.9 mg | 2.36 (2.11-2.65) | 2.26 (1.92-2.67) | 3.00 (2.54-3.54) | 2.42 (1.82-3.21) | 2.45 (1.84-3.26) | 1.64 (1.17-2.29) | | 1.77 (1.03-3.04) | |
| ≥25 mg | 3.59 (3.24-3.99) | 3.68 (3.12-4.36) | 4.85 (4.11-5.72) | 3.88 (3.12-4.82) | 3.14 (2.28-4.32) | 2.25 (1.68-3.01) | | 2.08 (1.16-3.74) | |
| **Cumulative dose per 1000 mg** | 1.01 (1.01-1.01) | 1.01 (1.01-1.01) | 1.01 (1.01-1.01) | 1.01 (1.01-1.01) | 1.01 (1.01-1.01) | 1.01 (1.01-1.01) | | 1.01 (1.00-1.01) | |
| **Cumulative dose category** (ref: non-use) | 1.00 | 1.00 | 1.00 | 1.00 | 1.00 | 1.00 | | 1.00 | |
| >0.0-959.9 mg | 1.36 (1.28-1.44) | 1.28 (1.17-1.40) | 1.48 (1.34-1.63) | 1.39 (1.22-1.58) | 1.29 (1.11-1.51) | 1.35 (1.20-1.53) | | 1.28 (0.99-1.65) | |
| 960-3054.9 mg | 1.35 (1.27-1.42) | 1.29 (1.19-1.41) | 1.42 (1.29-1.57) | 1.33 (1.17-1.51) | 1.30 (1.11-1.50) | 1.32 (1.17-1.49) | | 1.24 (0.97-1.60) | |
| 3055-7299.9 mg | 1.44 (1.36-1.52) | 1.37 (1.26-1.49) | 1.55 (1.41-1.71) | 1.49 (1.32-1.68) | 1.43 (1.24-1.66) | 1.35 (1.20-1.52) | | 1.26 (0.98-1.61) | |
| ≥7300 mg | 1.72 (1.63-1.82) | 1.57 (1.45-1.71) | 1.94 (1.78-2.13) | 1.94 (1.73-2.17) | 1.88 (1.64-2.16) | 1.45 (1.29-1.63) | | 1.59 (1.26-2.01) | |

Note: CI, confidence interval; CVD, cardiovascular disease; Hazard ratios from Cox proportional models adjusted for baseline age, sex, index of multiple deprivation, smoking status, ethnicity, body mass index, type of immune-mediated inflammatory disease, comorbidities (diabetes, diagnosed hypertension, cancer, asthma, chronic obstructive pulmonary disease, and renal disease), quintiles of biomarkers (systolic blood pressure, total cholesterol, high-density lipoprotein cholesterol, low-density lipoprotein cholesterol, c-reactive protein, creatinine), number of hospital admissions in last year, and prescribed non-oral glucocorticoids; and time-varying use of disease-modifying anti-rheumatic drugs and non-steroidal anti-inflammatory drugs; the practice identifier was included as a random intercept to account for clustering effect.

**Table M. Association between time variant oral glucocorticoid dose and 6 incident cardiovascular disease in patients with 6 immune-mediated inflammatory diseases from analysis in which biomarkers with over 60% missing data were excluded**

|  | **Adjusted hazard ratios with 95% CI** | | | | | |  | |  |
| --- | --- | --- | --- | --- | --- | --- | --- | --- | --- |
|  | **All CVD** | **Atrial fibrillation** | **Heart failure** | **Myocardial infarction** | **Peripheral arterial disease** | **Cerebrovascular disease** | | **Abdominal aortic aneurysm** | |
| **Ever use** (ref: non-use since 1 year prior to study entry) | 1.45 (1.39-1.51) | 1.37 (1.28-1.46) | 1.59 (1.48-1.72) | 1.53 (1.39-1.68) | 1.45 (1.30-1.62) | 1.36 (1.24-1.49) | | 1.34 (1.11-1.62) | |
| **Current use** (ref: non-use) | 1.92 (1.85-2.00) | 1.91 (1.81-2.02) | 2.19 (2.06-2.33) | 2.05 (1.89-2.22) | 1.99 (1.80-2.19) | 1.44 (1.33-1.57) | | 1.82 (1.55-2.13) | |
| **Current daily dose per 5 mg/day** | 1.09 (1.07-1.11) | 1.09 (1.07-1.11) | 1.10 (1.08-1.12) | 1.08 (1.06-1.10) | 1.08 (1.06-1.10) | 1.08 (1.06-1.10) | | 1.09 (1.06-1.12) | |
| **Current daily dose category** (ref: non-use) | 1.00 | 1.00 | 1.00 | 1.00 | 1.00 | 1.00 | | 1.00 | |
| >0.0-4.9 mg | 1.67 (1.55-1.79) | 1.68 (1.53-1.84) | 1.74 (1.55-1.96) | 1.73 (1.49-2.02) | 1.76 (1.51-2.05) | 1.30 (1.14-1.48) | | 1.91 (1.46-2.50) | |
| 5.0-14.9 mg | 1.87 (1.78-1.95) | 1.85 (1.73-1.99) | 2.12 (1.97-2.28) | 2.02 (1.83-2.24) | 1.95 (1.72-2.20) | 1.44 (1.31-1.59) | | 1.74 (1.42-2.13) | |
| 15.0-24.9 mg | 2.35 (2.10-2.64) | 2.26 (1.91-2.67) | 3.00 (2.54-3.54) | 2.42 (1.82-3.21) | 2.43 (1.83-3.24) | 1.63 (1.16-2.28) | | 1.78 (1.03-3.05) | |
| ≥25 mg | 3.58 (3.22-3.97) | 3.66 (3.10-4.33) | 4.85 (4.11-5.71) | 3.87 (3.11-4.80) | 3.12 (2.27-4.30) | 2.24 (1.67-2.99) | | 2.07 (1.15-3.72) | |
| **Cumulative dose per 1000 mg** | 1.01 (1.01-1.01) | 1.01 (1.01-1.01) | 1.01 (1.01-1.01) | 1.01 (1.01-1.01) | 1.01 (1.01-1.01) | 1.01 (1.01-1.01) | | 1.01 (1.00-1.01) | |
| **Cumulative dose category** (ref: non-use) | 1.00 | 1.00 | 1.00 | 1.00 | 1.00 | 1.00 | | 1.00 | |
| >0.0-959.9 mg | 1.36 (1.28-1.44) | 1.28 (1.17-1.40) | 1.48 (1.34-1.63) | 1.39 (1.22-1.58) | 1.29 (1.11-1.50) | 1.35 (1.19-1.52) | | 1.27 (0.98-1.65) | |
| 960-3054.9 mg | 1.33 (1.26-1.41) | 1.29 (1.18-1.40) | 1.41 (1.28-1.56) | 1.32 (1.16-1.50) | 1.27 (1.10-1.48) | 1.31 (1.16-1.47) | | 1.24 (0.97-1.60) | |
| 3055-7299.9 mg | 1.43 (1.36-1.51) | 1.37 (1.26-1.49) | 1.55 (1.41-1.71) | 1.48 (1.31-1.68) | 1.42 (1.23-1.64) | 1.33 (1.18-1.50) | | 1.25 (0.97-1.60) | |
| ≥7300 mg | 1.72 (1.63-1.81) | 1.56 (1.44-1.70) | 1.95 (1.78-2.14) | 1.94 (1.73-2.18) | 1.88 (1.64-2.15) | 1.44 (1.29-1.62) | | 1.59 (1.26-2.01) | |

Note: CI, confidence interval; CVD, cardiovascular disease; Hazard ratios from Cox proportional models adjusted for baseline age, sex, index of multiple deprivation, smoking status, ethnicity, body mass index, type of immune-mediated inflammatory disease, comorbidities (diabetes, diagnosed hypertension, cancer, asthma, chronic obstructive pulmonary disease, and renal disease), biomarkers (systolic blood pressure, creatinine), number of hospital admissions in last year, and prescribed non-oral glucocorticoids; and time-varying use of disease-modifying anti-rheumatic drugs and non-steroidal anti-inflammatory drugs; the practice identifier was included as a random intercept to account for clustering effect.

**Table N. Association between time variant oral glucocorticoid dose and incident all-cause cardiovascular disease by type of immune-mediated inflammatory disease, restricted to patients with newly diagnosed immune-mediated inflammatory disease**

|  | **Adjusted hazard ratios with 95% CI** | | | | | |
| --- | --- | --- | --- | --- | --- | --- |
|  | **All diseases*** | **PMR and/or GCA** | **Inflammatory bowel disease** | **Rheumatoid arthritis** | **Systemic lupus erythematosus** | **Vasculitis** |
| **No. of events** | 7,034 | 4,084 | 800 | 1,606 | 147 | 397 |
| **Ever use** (ref: non-use since 1 year prior to follow-up start) | 1.34 (1.25-1.43) | 1.11 (0.97-1.27) | 1.25 (1.08-1.46) | 1.55 (1.40-1.72) | 1.22 (0.60-2.47) | 1.52 (1.18-1.96) |
| **Current use** (ref: non-use) | 1.85 (1.75-1.96) | 1.64 (1.53-1.76) | 2.83 (2.39-3.36) | 2.18 (1.95-2.43) | 2.45 (1.06-5.67) | 3.25 (2.35-4.50) |
| **Current daily dose per 5 mg/day** | 1.14 (1.13-1.15) | 1.15 (1.12-1.18) | 1.12 (1.11-1.14) | 1.28 (1.24-1.32) | 1.51 (1.21-1.88) | 1.29 (1.20-1.38) |
| **Current daily dose category** (ref: non-use) | 1.00 | 1.00 | 1.00 | 1.00 | 1.00 | 1.00 |
| >0.0-4.9 mg | 1.66 (1.50-1.84) | 1.53 (1.36-1.72) | 2.23 (1.55-3.22) | 1.83 (1.46-2.29) | 2.97 (0.73-11.97) | 2.38 (1.08-5.25) |
| 5.0-14.9 mg | 1.76 (1.65-1.89) | 1.60 (1.47-1.74) | 2.45 (1.91-3.14) | 1.98 (1.72-2.27) | 0.90 (0.18-4.48) | 3.07 (1.84-5.13) |
| 15.0-24.9 mg | 2.20 (1.88-2.56) | 1.97 (1.66-2.33) | 3.34 (2.04-5.46) | 3.18 (2.25-4.49) | - | 2.69 (0.86-8.37) |
| ≥25 mg | 3.44 (2.95-4.01) | 2.61 (2.09-3.26) | 4.73 (3.42-6.56) | 5.44 (4.19-7.06) | 14.38 (3.43-60.25) | 5.94 (3.19-11.03) |
| **Cumulative dose per 1000 mg** | 1.02 (1.02-1.03) | 1.02 (1.01-1.02) | 1.03 (1.02-1.05) | 1.03 (1.02-1.04) | 1.01 (0.90-1.14) | 1.06 (1.01-1.10) |
| **Cumulative dose category** (ref: non-use) | 1.00 | 1.00 | 1.00 | 1.00 | 1.00 | 1.00 |
| >0.0-959.9 mg | 1.28 (1.17-1.39) | 1.07 (0.91-1.25) | 1.11 (0.91-1.36) | 1.44 (1.25-1.66) | 1.16 (0.41-3.24) | 1.48 (1.10-1.99) |
| 960-3054.9 mg | 1.25 (1.15-1.36) | 1.00 (0.87-1.16) | 1.23 (1.00-1.51) | 1.46 (1.24-1.71) | 1.88 (0.59-6.01) | 1.36 (0.83-2.24) |
| 3055-7299.9 mg | 1.39 (1.28-1.52) | 1.12 (0.97-1.29) | 1.41 (1.09-1.83) | 1.64 (1.39-1.94) | 1.07 (0.23-4.93) | 1.60 (0.93-2.73) |
| ≥7300 mg | 1.63 (1.49-1.79) | 1.30 (1.12-1.51) | 1.74 (1.31-2.32) | 1.78 (1.51-2.10) | 0.89 (0.21-3.78) | 2.81 (1.38-5.69) |

Note: CI, confidence interval; GCA, giant cell arteritis; Hazard ratios from Cox proportional imputed models adjusted for baseline age, sex, index of multiple deprivation, smoking status, ethnicity, body mass index, comorbidities (diabetes, diagnosed hypertension, cancer, asthma, chronic obstructive pulmonary disease, and renal disease), biomarkers (systolic blood pressure, total cholesterol, high-density lipoprotein cholesterol, low-density lipoprotein cholesterol, c-reactive protein, creatinine), number of hospital admissions in last year, and prescribed non-oral glucocorticoids; and time-varying use of disease-modifying anti-rheumatic drugs and non-steroidal anti-inflammatory drugs; the practice identifier was included as a random intercept to account for clustering effect. *These estimates were additionally adjusted for the type of immune-mediated inflammatory disease diagnosed. PMR, polymyalgia rheumatica.

**Table O. Association between time variant oral glucocorticoid dose and 6 incident cardiovascular diseases in patients with 6 immune-mediated inflammatory diseases, restricted to patients with newly diagnosed immune-mediated inflammatory disease**

|  | **Adjusted hazard ratios with 95% CI** | | | | | |  | |  |
| --- | --- | --- | --- | --- | --- | --- | --- | --- | --- |
|  | **All CVD** | **Atrial fibrillation** | **Heart failure** | **Myocardial infarction** | **Peripheral arterial disease** | **Cerebrovascular disease** | | **Abdominal aortic aneurysm** | |
| **Ever use** (ref: non-use since 1 year prior to follow-up start) | 1.34 (1.25-1.43) | 1.22 (1.10-1.35) | 1.45 (1.29-1.64) | 1.46 (1.25-1.70) | 1.37 (1.15-1.64) | 1.36 (1.17-1.59) | | 1.44 (1.07-1.96) | |
| **Current use** (ref: non-use) | 1.85 (1.75-1.96) | 1.87 (1.72-2.02) | 2.03 (1.85-2.22) | 1.89 (1.68-2.14) | 2.02 (1.75-2.34) | 1.36 (1.20-1.53) | | 1.73 (1.36-2.19) | |
| **Current daily dose per 5 mg/day** | 1.14 (1.13-1.15) | 1.15 (1.13-1.16) | 1.16 (1.15-1.18) | 1.15 (1.13-1.17) | 1.14 (1.12-1.17) | 1.11 (1.07-1.16) | | 1.11 (1.04-1.19) | |
| **Current daily dose category** (ref: non-use) | 1.00 | 1.00 | 1.00 | 1.00 | 1.00 | 1.00 | | 1.00 | |
| >0.0-4.9 mg | 1.66 (1.50-1.84) | 1.71 (1.49-1.96) | 1.67 (1.45-1.93) | 1.58 (1.25-1.99) | 1.89 (1.46-2.45) | 1.23 (1.00-1.52) | | 1.79 (1.15-2.77) | |
| 5.0-14.9 mg | 1.76 (1.65-1.89) | 1.79 (1.62-1.97) | 1.94 (1.73-2.18) | 1.87 (1.59-2.20) | 1.88 (1.53-2.32) | 1.35 (1.15-1.59) | | 1.65 (1.19-2.27) | |
| 15.0-24.9 mg | 2.20 (1.88-2.56) | 2.01 (1.66-2.44) | 2.74 (2.16-3.47) | 2.20 (1.54-3.15) | 2.60 (1.84-3.67) | 1.46 (0.92-2.33) | | 1.64 (0.77-3.49) | |
| ≥25 mg | 3.44 (2.95-4.01) | 3.56 (2.83-4.46) | 4.47 (3.58-5.57) | 3.80 (2.81-5.13) | 3.33 (2.22-5.01) | 2.12 (1.49-3.01) | | 2.12 (0.94-4.76) | |
| **Cumulative dose per 1000 mg** | 1.02 (1.02-1.03) | 1.02 (1.01-1.02) | 1.03 (1.02-1.03) | 1.02 (1.02-1.03) | 1.03 (1.02-1.03) | 1.01 (1.00-1.02) | | 1.01 (0.99-1.02) | |
| **Cumulative dose category** (ref: non-use) | 1.00 | 1.00 | 1.00 | 1.00 | 1.00 | 1.00 | | 1.00 | |
| >0.0-959.9 mg | 1.28 (1.17-1.39) | 1.14 (1.00-1.29) | 1.39 (1.20-1.61) | 1.39 (1.15-1.68) | 1.19 (0.94-1.49) | 1.36 (1.13-1.63) | | 1.42 (0.98-2.06) | |
| 960-3054.9 mg | 1.25 (1.15-1.36) | 1.15 (1.02-1.30) | 1.31 (1.13-1.51) | 1.30 (1.07-1.57) | 1.29 (1.04-1.62) | 1.27 (1.06-1.53) | | 1.39 (0.96-2.00) | |
| 3055-7299.9 mg | 1.39 (1.28-1.52) | 1.31 (1.15-1.48) | 1.48 (1.28-1.71) | 1.43 (1.18-1.73) | 1.42 (1.13-1.78) | 1.41 (1.17-1.69) | | 1.44 (0.99-2.10) | |
| ≥7300 mg | 1.63 (1.49-1.79) | 1.44 (1.26-1.64) | 1.82 (1.57-2.12) | 1.92 (1.59-2.33) | 1.98 (1.57-2.50) | 1.46 (1.21-1.78) | | 1.64 (1.10-2.43) | |

Note: CI, confidence interval; CVD, cardiovascular disease; Hazard ratios from Cox proportional imputed models adjusted for baseline age, sex, index of multiple deprivation, smoking status, ethnicity, body mass index, type of immune-mediated inflammatory disease, comorbidities (diabetes, diagnosed hypertension, cancer, asthma, chronic obstructive pulmonary disease, and renal disease), biomarkers (systolic blood pressure, total cholesterol, high-density lipoprotein cholesterol, low-density lipoprotein cholesterol, c-reactive protein, creatinine), number of hospital admissions in last year, and prescribed non-oral glucocorticoids; and time-varying use of disease-modifying anti-rheumatic drugs and non-steroidal anti-inflammatory drugs; the practice identifier was included as a random intercept to account for clustering effect.

**Table P. Association between time variant oral glucocorticoid dose and 6 incident cardiovascular diseases in patients with 6 immune-mediated inflammatory diseases, restricted to patients diagnosed with immune-mediated inflammatory diseases within 2 years**

|  | **Adjusted hazard ratios with 95% CI** | | | | | |  |
| --- | --- | --- | --- | --- | --- | --- | --- |
|  | **All CVD** | **Atrial fibrillation** | **Heart failure** | **Myocardial infarction** | **Peripheral arterial disease** | **Cerebrovascular disease** | **Abdominal aortic aneurysm** |
| **No. of events** | 1,845 | 800 | 682 | 399 | 279 | 441 | 94 |
| **Ever use** (ref: non-use since 1 year prior to follow-up start) | 1.40 (1.24-1.57) | 1.38 (1.15-1.67) | 1.49 (1.21-1.83) | 1.19 (0.93-1.53) | 1.60 (1.19-2.17) | 1.24 (0.97-1.59) | 2.14 (1.17- 3.92) |
| **Current use** (ref: non-use) | 1.73 (1.57-1.92) | 1.92 (1.64-2.24) | 1.85 (1.57-2.18) | 1.90 (1.53-2.36) | 1.70 (1.31-2.21) | 1.29 (1.04-1.60) | 2.20 (1.41- 3.44) |
| **Current daily dose per 5 mg/day** | 1.09 (1.03-1.15) | 1.04 (0.90-1.21) | 1.08 (1.06-1.11) | 1.06 (1.04-1.09) | 1.07 (1.03-1.10) | 1.11 (0.89-1.39) | 1.09 (1.01- 1.17) |
| **Current daily dose category** (ref: non-use) | 1.00 | 1.00 | 1.00 | 1.00 | 1.00 | 1.00 | 1.00 |
| >0.0-4.9 mg | 1.39 (1.18-1.64) | 1.45 (1.12-1.89) | 1.44 (1.11-1.87) | 1.70 (1.17-2.47) | 1.28 (0.74-2.22) | 1.22 (0.85-1.74) | 2.38 (1.20- 4.70) |
| 5.0-14.9 mg | 1.74 (1.54-1.97) | 2.03 (1.69-2.44) | 1.82 (1.48-2.24) | 1.86 (1.38-2.50) | 1.79 (1.30-2.47) | 1.24 (0.93-1.66) | 2.18 (1.20- 3.97) |
| 15.0-24.9 mg | 2.31 (1.69-3.15) | 2.50 (1.54-4.08) | 2.19 (1.35-3.57) | 2.01 (0.96-4.20) | 2.20 (0.97-4.96) | 2.00 (1.14-3.50) | - |
| ≥25 mg | 3.39 (2.47-4.66) | 3.22 (2.00-5.19) | 5.12 (3.40-7.70) | 3.53 (1.83-6.81) | 2.48 (0.93-6.63) | 1.41 (0.52-3.85) | 2.09 (0.32-13.84) |
| **Cumulative dose per 1000 mg** | 1.01 (1.00-1.01) | 1.01 (1.00-1.01) | 1.01 (1.00-1.01) | 1.01 (1.00-1.01) | 1.01 (1.00-1.01) | 1.00 (0.99-1.01) | 1.01 (1.00- 1.02) |
| **Cumulative dose category** (ref: non-use) | 1.00 | 1.00 | 1.00 | 1.00 | 1.00 | 1.00 | 1.00 |
| >0.0-959.9 mg | 1.40 (1.19-1.64) | 1.28 (1.00-1.66) | 1.61 (1.24-2.09) | 1.03 (0.72-1.48) | 1.77 (1.20-2.60) | 1.38 (1.00-1.91) | 1.64 (0.73- 3.69) |
| 960-3054.9 mg | 1.28 (1.09-1.50) | 1.24 (0.97-1.59) | 1.28 (0.97-1.68) | 1.02 (0.72-1.44) | 1.55 (1.03-2.31) | 1.20 (0.86-1.67) | 1.95 (0.90- 4.23) |
| 3055-7299.9 mg | 1.28 (1.10-1.49) | 1.29 (1.02-1.64) | 1.35 (1.04-1.74) | 1.05 (0.75-1.47) | 1.35 (0.90-2.03) | 1.07 (0.77-1.47) | 2.28 (1.11- 4.70) |
| ≥7300 mg | 1.63 (1.41-1.89) | 1.70 (1.36-2.12) | 1.69 (1.32-2.15) | 1.68 (1.24-2.27) | 1.69 (1.17-2.45) | 1.30 (0.96-1.75) | 2.68 (1.35- 5.32) |

Note: CI, confidence interval; CVD, cardiovascular disease; Hazard ratios from Cox proportional imputed models adjusted for baseline age, sex, index of multiple deprivation, smoking status, ethnicity, body mass index, comorbidities (diabetes, diagnosed hypertension, cancer, asthma, chronic obstructive pulmonary disease, and renal disease), biomarkers (systolic blood pressure, total cholesterol, high-density lipoprotein cholesterol, low-density lipoprotein cholesterol, c-reactive protein, creatinine), number of hospital admissions in last year, and prescribed non-oral glucocorticoids; and time-varying use of disease-modifying anti-rheumatic drugs and non-steroidal anti-inflammatory drugs; the practice identifier was included as a random intercept to account for clustering effect. *These estimates were additionally adjusted for the type of immune-mediated inflammatory disease diagnosed

**Table Q. Association between time variant oral glucocorticoid dose and 6 incident cardiovascular diseases in patients with 6 immune-mediated inflammatory diseases, restricted to patients diagnosed with immune-mediated inflammatory diseases for over 2 years**

|  | **Adjusted hazard ratios with 95% CI** | | | | | |  |
| --- | --- | --- | --- | --- | --- | --- | --- |
|  | **All CVD** | **Atrial fibrillation** | **Heart failure** | **Myocardial infarction** | **Peripheral arterial disease** | **Cerebrovascular disease** | **Abdominal aortic aneurysm** |
| **No. of events** | 4,547 | 1,924 | 1,595 | 1,028 | 709 | 1,003 | 243 |
| **Ever use** (ref: non-use since 1 year prior to follow-up start) | 1.56 (1.47-1.67) | 1.50 (1.36-1.66) | 1.78 (1.59-2.00) | 1.19 (0.93-1.53) | 1.58 (1.34-1.86) | 1.37 (1.19-1.57) | 1.21 (0.91-1.61) |
| **Current use** (ref: non-use) | 2.03 (1.91-2.16) | 1.94 (1.76-2.13) | 2.55 (2.30-2.83) | 1.90 (1.53-2.36) | 2.10 (1.80-2.47) | 1.60 (1.40-1.83) | 1.76 (1.34-2.30) |
| **Current daily dose per 5 mg/day** | 1.18 (1.16-1.20) | 1.18 (1.16-1.21) | 1.21 (1.19-1.24) | 1.06 (1.04-1.09) | 1.17 (1.13-1.21) | 1.15 (1.10-1.19) | 1.14 (1.05-1.23) |
| **Current daily dose category** (ref: non-use) | 1.00 | 1.00 | 1.00 | 1.00 | 1.00 | 1.00 | 1.00 |
| >0.0-4.9 mg | 1.71 (1.54-1.90) | 1.67 (1.43-1.95) | 1.95 (1.58-2.42) | 1.70 (1.17-2.47) | 1.78 (1.32-2.39) | 1.42 (1.08-1.86) | 1.81 (1.10-2.96) |
| 5.0-14.9 mg | 1.99 (1.85-2.15) | 1.85 (1.64-2.10) | 2.49 (2.20-2.82) | 1.86 (1.38-2.50) | 2.14 (1.78-2.56) | 1.59 (1.35-1.87) | 1.70 (1.19-2.43) |
| 15.0-24.9 mg | 2.61 (2.17-3.13) | 2.57 (1.87-3.52) | 3.97 (3.05-5.16) | 2.01 (0.96-4.20) | 2.26 (1.21-4.24) | 1.75 (1.06-2.86) | 1.81 (0.67-4.90) |
| ≥25 mg | 3.93 (3.24-4.78) | 3.93 (2.96-5.20) | 5.50 (4.14-7.29) | 3.53 (1.83-6.81) | 3.34 (2.05-5.44) | 2.78 (1.56-4.94) | 1.92 (0.67-5.48) |
| **Cumulative dose per 1000 mg** | 1.02 (1.02-1.02) | 1.01 (1.01-1.02) | 1.02 (1.02-1.02) | 1.01 (1.00-1.01) | 1.02 (1.02-1.03) | 1.01 (1.00-1.02) | 1.01 (1.00-1.03) |
| **Cumulative dose category** (ref: non-use) | 1.00 | 1.00 | 1.00 | 1.00 | 1.00 | 1.00 | 1.00 |
| >0.0-959.9 mg | 1.36 (1.24-1.50) | 1.39 (1.20-1.61) | 1.47 (1.25-1.74) | 1.03 (0.72-1.48) | 1.23 (0.96-1.59) | 1.26 (1.02-1.54) | 1.04 (0.67-1.60) |
| 960-3054.9 mg | 1.47 (1.33-1.62) | 1.47 (1.27-1.71) | 1.62 (1.36-1.92) | 1.02 (0.72-1.44) | 1.24 (0.96-1.62) | 1.44 (1.18-1.77) | 1.09 (0.70-1.68) |
| 3055-7299.9 mg | 1.59 (1.45-1.74) | 1.46 (1.26-1.69) | 1.76 (1.50-2.06) | 1.05 (0.75-1.47) | 1.72 (1.37-2.17) | 1.33 (1.09-1.63) | 1.10 (0.72-1.68) |
| ≥7300 mg | 1.78 (1.64-1.93) | 1.63 (1.44-1.85) | 2.15 (1.87-2.46) | 1.68 (1.24-2.27) | 1.99 (1.63-2.44) | 1.43 (1.20-1.71) | 1.48 (1.05-2.09) |

Note: CI, confidence interval; CVD, cardiovascular disease; Hazard ratios from Cox proportional imputed models adjusted for baseline age, sex, index of multiple deprivation, smoking status, ethnicity, body mass index, comorbidities (diabetes, diagnosed hypertension, cancer, asthma, chronic obstructive pulmonary disease, and renal disease), biomarkers (systolic blood pressure, total cholesterol, high-density lipoprotein cholesterol, low-density lipoprotein cholesterol, c-reactive protein, creatinine), number of hospital admissions in last year, and prescribed non-oral glucocorticoids; and time-variant use of disease-modifying anti-rheumatic drugs and non-steroidal anti-inflammatory drugs; the practice identifier was included as a random intercept to account for clustering effect. *These estimates were additionally adjusted for the type of immune-mediated inflammatory disease diagnosed

**Table R. Association between time variant oral glucocorticoid dose and 6 incident cardiovascular diseases in patients with 6 immune-mediated inflammatory diseases, adjusted for periods of flare during follow-up (defined by biomarker or 5 mg daily dose increase)**

|  | **Adjusted hazard ratios with 95% CI** | | | | | |  |
| --- | --- | --- | --- | --- | --- | --- | --- |
|  | **All CVD** | **Atrial fibrillation** | **Heart failure** | **Myocardial infarction** | **Peripheral arterial disease** | **Cerebrovascular disease** | **Abdominal aortic aneurysm** |
| **Ever use** (ref: non-use since 1 year prior to follow-up start) | 1.34 (1.27-1.41) | 1.26 (1.16-1.37) | 1.39 (1.27-1.52) | 1.40 (1.25-1.58) | 1.35 (1.17-1.56) | 1.35 (1.21-1.51) | 1.20 (0.95-1.52) |
| **Current use** (ref: non-use) | 1.86 (1.79-1.94) | 1.88 (1.77-1.99) | 2.07 (1.95-2.21) | 1.97 (1.81-2.14) | 1.92 (1.74-2.13) | 1.41 (1.30-1.53) | 1.75 (1.48-2.07) |
| **Current daily dose per 5 mg/day** | 1.09 (1.07-1.10) | 1.09 (1.07-1.11) | 1.10 (1.08-1.12) | 1.08 (1.06-1.10) | 1.08 (1.06-1.10) | 1.08 (1.06-1.10) | 1.08 (1.05-1.11) |
| **Current daily dose category** (ref: non-use) | 1.00 | 1.00 | 1.00 | 1.00 | 1.00 | 1.00 | 1.00 |
| >0.0-4.9 mg | 1.62 (1.51-1.74) | 1.65 (1.50-1.80) | 1.66 (1.47-1.87) | 1.67 (1.44-1.95) | 1.71 (1.46-2.00) | 1.27 (1.11-1.46) | 1.84 (1.40-2.42) |
| 5.0-14.9 mg | 1.81 (1.73-1.89) | 1.82 (1.69-1.96) | 2.01 (1.87-2.17) | 1.94 (1.75-2.15) | 1.88 (1.65-2.13) | 1.40 (1.27-1.55) | 1.68 (1.37-2.07) |
| 15.0-24.9 mg | 2.28 (2.03-2.56) | 2.21 (1.87-2.61) | 2.85 (2.41-3.36) | 2.33 (1.74-3.12) | 2.37 (1.77-3.17) | 1.60 (1.14-2.24) | 1.71 (0.99-2.95) |
| ≥25 mg | 3.48 (3.13-3.86) | 3.60 (3.05-4.25) | 4.61 (3.91-5.44) | 3.73 (2.99-4.64) | 3.04 (2.20-4.20) | 2.19 (1.64-2.93) | 2.01 (1.11-3.63) |
| **Cumulative dose per 1000 mg** | 1.01 (1.01-1.01) | 1.01 (1.01-1.01) | 1.01 (1.01-1.01) | 1.01 (1.01-1.01) | 1.01 (1.01-1.01) | 1.01 (1.00-1.01) | 1.01 (1.00-1.01) |
| **Cumulative dose category** (ref: non-use) | 1.00 | 1.00 | 1.00 | 1.00 | 1.00 | 1.00 | 1.00 |
| >0.0-959.9 mg | 1.27 (1.19-1.35) | 1.19 (1.08-1.31) | 1.31 (1.17-1.46) | 1.28 (1.11-1.48) | 1.21 (1.02-1.44) | 1.34 (1.17-1.53) | 1.16 (0.87-1.54) |
| 960-3054.9 mg | 1.24 (1.17-1.32) | 1.19 (1.07-1.32) | 1.24 (1.11-1.38) | 1.22 (1.05-1.41) | 1.20 (1.01-1.43) | 1.31 (1.14-1.50) | 1.11 (0.83-1.48) |
| 3055-7299.9 mg | 1.32 (1.24-1.41) | 1.25 (1.13-1.38) | 1.34 (1.20-1.50) | 1.36 (1.17-1.57) | 1.32 (1.11-1.58) | 1.33 (1.16-1.53) | 1.11 (0.83-1.49) |
| ≥7300 mg | 1.59 (1.49-1.69) | 1.44 (1.31-1.59) | 1.69 (1.53-1.88) | 1.78 (1.55-2.04) | 1.74 (1.48-2.06) | 1.43 (1.25-1.64) | 1.42 (1.08-1.87) |

Note: CI, confidence interval; CVD, cardiovascular disease; Hazard ratios from Cox proportional imputed models adjusted for baseline age, sex, index of multiple deprivation, smoking status, ethnicity, body mass index, type of immune-mediated inflammatory disease, comorbidities (diabetes, diagnosed hypertension, cancer, asthma, chronic obstructive pulmonary disease, and renal disease), biomarkers (systolic blood pressure, total cholesterol, high-density lipoprotein cholesterol, low-density lipoprotein cholesterol, c-reactive protein, creatinine), number of hospital admissions in last year, and prescribed non-oral glucocorticoids; time-variant use of disease-modifying anti-rheumatic drugs and non-steroidal anti-inflammatory drugs; and time-variant disease activity period; the practice identifier was included as a random intercept to account for clustering effect.

**Table S. Association between time variant oral glucocorticoid dose and 6 incident cardiovascular diseases in patients with 6 immune-mediated inflammatory diseases, adjusted for periods of flare during follow-up (defined by biomarker or 10 mg daily dose increase)**

|  | **Adjusted hazard ratios with 95% CI** | | | | | |  |
| --- | --- | --- | --- | --- | --- | --- | --- |
|  | **All CVD** | **Atrial fibrillation** | **Heart failure** | **Myocardial infarction** | **Peripheral arterial disease** | **Cerebrovascular disease** | **Abdominal aortic aneurysm** |
| **Ever use** (ref: non-use since 1 year prior to follow-up start) | 1.34 (1.28-1.41) | 1.26 (1.17-1.36) | 1.40 (1.28-1.52) | 1.39 (1.25-1.54) | 1.35 (1.19-1.54) | 1.36 (1.23-1.51) | 1.19 (0.96-1.48) |
| **Current use** (ref: non-use) | 1.86 (1.79-1.94) | 1.87 (1.76-1.98) | 2.06 (1.93-2.20) | 1.96 (1.80-2.13) | 1.92 (1.73-2.13) | 1.42 (1.31-1.55) | 1.73 (1.47-2.05) |
| **Current daily dose per 5 mg/day** | 1.09 (1.07-1.10) | 1.09 (1.07-1.11) | 1.10 (1.08-1.12) | 1.08 (1.06-1.10) | 1.08 (1.06-1.10) | 1.08 (1.06-1.10) | 1.08 (1.05-1.11) |
| **Current daily dose category** (ref: non-use) | 1.00 | 1.00 | 1.00 | 1.00 | 1.00 | 1.00 | 1.00 |
| >0.0-4.9 mg | 1.62 (1.50-1.74) | 1.64 (1.49-1.80) | 1.65 (1.46-1.87) | 1.67 (1.42-1.95) | 1.71 (1.47-1.99) | 1.28 (1.12-1.47) | 1.82 (1.39-2.39) |
| 5.0-14.9 mg | 1.81 (1.72-1.89) | 1.81 (1.68-1.95) | 2.00 (1.86-2.16) | 1.93 (1.74-2.14) | 1.88 (1.64-2.15) | 1.42 (1.28-1.57) | 1.66 (1.35-2.04) |
| 15.0-24.9 mg | 2.27 (2.03-2.54) | 2.19 (1.85-2.59) | 2.82 (2.38-3.33) | 2.31 (1.73-3.07) | 2.36 (1.78-3.13) | 1.61 (1.16-2.25) | 1.69 (0.98-2.91) |
| ≥25 mg | 3.46 (3.11-3.84) | 3.57 (3.03-4.22) | 4.56 (3.86-5.38) | 3.69 (2.98-4.58) | 3.03 (2.19-4.19) | 2.21 (1.65-2.97) | 1.98 (1.09-3.58) |
| **Cumulative dose per 1000 mg** | 1.01 (1.01-1.01) | 1.01 (1.01-1.01) | 1.01 (1.01-1.01) | 1.01 (1.01-1.01) | 1.01 (1.01-1.01) | 1.01 (1.00-1.01) | 1.01 (1.00-1.01) |
| **Cumulative dose category** (ref: non-use) | 1.00 | 1.00 | 1.00 | 1.00 | 1.00 | 1.00 | 1.00 |
| >0.0-959.9 mg | 1.28 (1.20-1.36) | 1.20 (1.09-1.31) | 1.33 (1.20-1.48) | 1.29 (1.13-1.47) | 1.22 (1.04-1.44) | 1.35 (1.19-1.53) | 1.16 (0.88-1.52) |
| 960-3054.9 mg | 1.25 (1.18-1.33) | 1.20 (1.09-1.31) | 1.26 (1.13-1.40) | 1.22 (1.07-1.40) | 1.22 (1.03-1.43) | 1.32 (1.16-1.50) | 1.11 (0.85-1.46) |
| 3055-7299.9 mg | 1.33 (1.25-1.41) | 1.25 (1.14-1.37) | 1.35 (1.21-1.50) | 1.35 (1.18-1.54) | 1.33 (1.13-1.58) | 1.34 (1.18-1.53) | 1.10 (0.84-1.45) |
| ≥7300 mg | 1.58 (1.49-1.68) | 1.44 (1.31-1.57) | 1.69 (1.53-1.87) | 1.76 (1.55-1.99) | 1.75 (1.49-2.05) | 1.45 (1.27-1.65) | 1.39 (1.07-1.81) |

Note: CI, confidence interval; CVD, cardiovascular disease; Hazard ratios from Cox proportional imputed models adjusted for baseline age, sex, index of multiple deprivation, smoking status, ethnicity, body mass index, type of immune-mediated inflammatory disease, comorbidities (diabetes, diagnosed hypertension, cancer, asthma, COPD, and renal disease), biomarkers (systolic blood pressure, total cholesterol, high-density lipoprotein cholesterol, low-density lipoprotein cholesterol, c-reactive protein, creatinine), number of hospital admissions in last year, and prescribed non-oral glucocorticoids; time-variant use of disease-modifying anti-rheumatic drugs and non-steroidal anti-inflammatory drugs; and time-variant disease activity period; the practice identifier was included as a random intercept to account for clustering effect.

**Table T.** **Associations between time variant oral glucocorticoid prednisolone-equivalent dose and incident all-cause cardiovascular disease by immune-mediated inflammatory disease, according to the number of years of exposure considered prior to follow-up start [Additional sensitivity analysis]**

|  | **Adjusted hazard ratios with 95% CI** | | | | | |
| --- | --- | --- | --- | --- | --- | --- |
|  | **All diseases*** | **PMR and/or GCA** | **Inflammatory bowel disease** | **Rheumatoid arthritis** | **Systemic lupus erythematosus** | **Vasculitis** |
| **No. of events** | 13,426 | 6,267 | 1,937 | 4,236 | 375 | 611 |
| **Ever use** (ref: non-use since 1 year prior to follow-up start) | 1.46 (1.40-1.53) | 1.25 (1.15-1.37) | 1.39 (1.26-1.52) | 1.63 (1.52-1.73) | 1.69 (1.35-2.12) | 1.52 (1.28-1.81) |
| **Ever use** (ref: non-use since 2 years prior to follow-up start) | 1.50 (1.42-1.58) | 1.28 (1.16-1.41) | 1.44 (1.29-1.59) | 1.67 (1.54-1.80) | 1.73 (1.29-2.28) | 1.53 (1.34-1.89) |
| **Ever use** (ref: non-use since 5 years prior to follow-up start) | 1.57 (1.50-1.65) | 1.33 (1.21- 1.45) | 1.49 (1.34-1.64) | 1.75 (1.63-1.87) | 1.80 (1.35-2.39) | 1.58 (1.38-1.77) |
| **Cumulative dose category** (ref: non-use since 1 year prior to follow-up start) | 1.00 | 1.00 | 1.00 | 1.00 | 1.00 | 1.00 |
| >0.0-959.9 mg | 1.37 (1.29-1.45) | 1.24 (1.11-1.38) | 1.21 (1.06-1.38) | 1.47 (1.34-1.61) | 1.65 (1.20- 2.26) | 1.54 (1.23-1.93) |
| 960-3054.9 mg | 1.35 (1.28-1.43) | 1.14 (1.04-1.26) | 1.35 (1.18-1.54) | 1.52 (1.36-1.68) | 1.40 (0.95- 2.06) | 1.31 (0.95-1.82) |
| 3055-7299.9 mg | 1.44 (1.36-1.52) | 1.21 (1.10-1.33) | 1.45 (1.24-1.69) | 1.72 (1.55-1.90) | 1.60 (1.10- 2.34) | 1.27 (0.94-1.70) |
| ≥7300 mg | 1.76 (1.66-1.86) | 1.50 (1.36-1.66) | 1.85 (1.58-2.16) | 1.80 (1.65-1.97) | 2.09 (1.53- 2.85) | 1.91 (1.49-2.45) |
| **Cumulative dose category** (ref: non-use since 2 years prior to follow-up start) | 1.00 | 1.00 | 1.00 | 1.00 | 1.00 | 1.00 |
| >0.0-959.9 mg | 1.37 (1.30-1.45) | 1.25 (1.11-1.39) | 1.21 (1.07-1.38) | 1.47 (1.34-1.60) | 1.66 (1.22-2.27) | 1.54 (1.24-1.93) |
| 960-3054.9 mg | 1.37 (1.31-1.45) | 1.15 (1.06-1.25) | 1.35 (1.19-1.52) | 1.54 (1.39-1.69) | 1.41 (0.97-2.05) | 1.30 (0.96-1.81) |
| 3055-7299.9 mg | 1.47 (1.40-1.55) | 1.25 (1.15-1.35) | 1.47 (1.26-1.72) | 1.77 (1.60-1.94) | 1.63 (1.13-2.36) | 1.29 (0.95-1.72) |
| ≥7300 mg | 1.78 (1.69-1.87) | 1.52 (1.39 -1.66) | 1.87 (1.61-2.18) | 1.81 (1.67-1.96) | 2.13 (1.56-2.88) | 1.92 (1.50-2.46) |
| **Cumulative dose category** (ref: non-use since 5 years prior to follow-up start) | 1.00 | 1.00 | 1.00 | 1.00 | 1.00 | 1.00 |
| >0.0-959.9 mg | 1.37 (1.30-1.44) | 1.25 (1.12-1.38) | 1.21 (1.07-1.38) | 1.47 (1.34-1.60) | 1.66 (1.22- 2.26) | 1.54 (1.24-1.92) |
| 960-3054.9 mg | 1.37 (1.32-1.43) | 1.15 (1.05-1.26) | 1.36 (1.20-1.52) | 1.55 (1.40-1.66) | 1.41 (0.97- 2.04) | 1.31 (0.96-1.82) |
| 3055-7299.9 mg | 1.49 (1.42-1.54) | 1.27 (1.18-1.36) | 1.48 (1.28-1.69) | 1.78 (1.62-1.94) | 1.63 (1.13- 2.34) | 1.29 (0.95-1.71) |
| ≥7300 mg | 1.79 (1.72-1.86) | 1.54 (1.42-1.66) | 1.87 (1.61-2.17) | 1.83 (1.70-1.94) | 2.14 (1.58- 2.89) | 1.93 (1.51-2.46) |

Note: CI, confidence interval; Hazard ratios from Cox proportional imputed models adjusted for baseline age, sex, index of multiple deprivation, smoking status, ethnicity, body mass index, comorbidities (diabetes, diagnosed hypertension, cancer, asthma, chronic obstructive pulmonary disease, and renal disease), biomarkers (total cholesterol, high-density lipoprotein cholesterol, low-density lipoprotein cholesterol, c-reactive protein, creatinine), number of hospital admissions in last year, and prescribed non-oral glucocorticoids; and time-variant use of disease-modifying anti-rheumatic drugs and non-steroidal anti-inflammatory drugs; the practice identifier was included as a random intercept to account for clustering effect. *These estimates were additionally adjusted for the type of immune-mediated inflammatory disease diagnosed.

**Table U. Associations between time variant oral glucocorticoid prednisolone-equivalent dose and incident all-cause cardiovascular disease by immune-mediated inflammatory disease, adjusted for propensity score for prescribing indication [Additional sensitivity analysis]**

|  | **Adjusted hazard ratios with 95% CI** | | | | | |
| --- | --- | --- | --- | --- | --- | --- |
|  | **All diseases*** | **PMR and/or GCA** | **Inflammatory bowel disease** | **Rheumatoid arthritis** | **Systemic lupus erythematosus** | **Vasculitis** |
| **No. of events** | 13,426 | 6,267 | 1,937 | 4,236 | 375 | 611 |
| **Ever use** (ref: non-use since 1 year prior to follow-up start) | 1.46 (1.40-1.52) | 1.25 (1.15-1.36) | 1.38 (1.25-1.52) | 1.62 (1.52-1.73) | 1.69 (1.35- 2.12) | 1.54 (1.30-1.83) |
| **Current use** (ref: non-use) | 1.95 (1.87-2.02) | 1.69 (1.60-1.78) | 2.70 (2.42-3.02) | 2.10 (1.97-2.24) | 2.56 (2.02- 3.25) | 2.08 (1.72-2.51) |
| **Current daily dose per 5 mg/day** | 1.09 (1.07-1.12) | 1.17 (1.15-1.19) | 1.08 (1.06-1.09) | 1.28 (1.25-1.30) | 1.28 (1.20- 1.37) | 1.19 (1.13-1.26) |
| **Current daily dose category** (ref: non-use) | 1.00 | 1.00 | 1.00 | 1.00 | 1.00 | 1.00 |
| >0.0-4.9 mg | 1.68 (1.57-1.81) | 1.50 (1.37-1.64) | 2.16 (1.65-2.81) | 1.83 (1.61-2.09) | 2.81 (1.92- 4.12) | 1.93 (1.35-2.75) |
| 5.0-14.9 mg | 1.89 (1.81-1.98) | 1.70 (1.59-1.82) | 2.39 (2.03-2.81) | 1.99 (1.85-2.15) | 2.18 (1.63- 2.94) | 1.92 (1.51-2.45) |
| 15.0-24.9 mg | 2.38 (2.13-2.67) | 2.07 (1.80-2.38) | 3.43 (2.49-4.73) | 2.78 (2.21-3.50) | 2.61 (1.13- 6.01) | 2.46 (1.54-3.95) |
| ≥25 mg | 3.63 (3.27-4.03) | 2.76 (2.30-3.32) | 4.35 (3.43-5.51) | 4.95 (4.09-5.99) | 5.67 (3.21-10.01) | 3.11 (1.95-4.97) |
| **Cumulative dose per 1000 mg** | 1.01 (1.01-1.01) | 1.02 (1.01-1.02) | 1.01 (1.01-1.01) | 1.02 (1.02-1.03) | 1.03 (1.01- 1.04) | 1.03 (1.02-1.04) |
| **Cumulative dose category** (ref: non-use) | 1.00 | 1.00 | 1.00 | 1.00 | 1.00 | 1.00 |
| >0.0-959.9 mg | 1.37 (1.29-1.45) | 1.24 (1.11-1.38) | 1.20 (1.05-1.37) | 1.46 (1.33-1.60) | 1.65 (1.20- 2.26) | 1.56 (1.25-1.96) |
| 960-3054.9 mg | 1.35 (1.27-1.43) | 1.14 (1.04-1.26) | 1.34 (1.17-1.53) | 1.51 (1.36-1.68) | 1.40 (0.95- 2.06) | 1.32 (0.95-1.83) |
| 3055-7299.9 mg | 1.43 (1.36-1.52) | 1.21 (1.10-1.33) | 1.44 (1.24-1.68) | 1.71 (1.55-1.89) | 1.60 (1.10- 2.34) | 1.28 (0.95-1.72) |
| ≥7300 mg | 1.75 (1.66-1.85) | 1.50 (1.36-1.66) | 1.84 (1.58-2.15) | 1.79 (1.64-1.96) | 2.10 (1.53- 2.88) | 1.93 (1.51-2.47) |

Note: CI, confidence interval; Hazard ratios from Cox proportional imputed models adjusted for baseline age, sex, index of multiple deprivation, smoking status, ethnicity, body mass index, comorbidities (diabetes, diagnosed hypertension, cancer, asthma, chronic obstructive pulmonary disease, and renal disease), biomarkers (total cholesterol, high-density lipoprotein cholesterol, low-density lipoprotein cholesterol, c-reactive protein, creatinine), number of hospital admissions in last year, and prescribed non-oral glucocorticoids; and time-variant use of disease-modifying anti-rheumatic drugs and non-steroidal anti-inflammatory drugs; the practice identifier was included as a random intercept to account for clustering effect. *These estimates were additionally adjusted for the type of immune-mediated inflammatory disease diagnosed. Propensity score for prescribing indication was also adjusted.

**Table V. Summary of the methodology and major findings of previous studies investigating the association between glucocorticoid dose and cardiovascular diseases**

| **Publication** | **IMID** | **Sample size** | **Glucocorticoid quantification** | **Confounders considered** | **Outcome measure** | **Major findings** |
| --- | --- | --- | --- | --- | --- | --- |
| Karp et al (1) | SLE | 310 | Mean dose in past year | Age, sex, other IMID medication, IMID duration and activity | Cardiovascular risk profile | Dose correlation with estimated 2 year cardiovascular risk (p<0.05) |
| Souverin et al (2) | RA | 1515 | Current, recent or past use | Other IMID and CV drug use, smoking, BMI, number of physician visits | IHD; HF; TIA or stroke | OR 1.37 (1.16 to 1.62) for current use vs. none |
| Wei et al (3) | IA, IBD | 2918 | Mean daily dose (low, medium or high) | Ages, sex, SED, IMID and CV drug use; comorbid illness | IHD; HF; TIA/stroke; CV death | RR 3.30 (1.56-6.96) IA high-dose users vs. IA non-users. No association found within IBD group |
| Christiansen et al (7) | CTD, RA | 258 | Current, former or never used | Other IMID and CV drug use, comorbid illness | AF/flutter | OR 1.34 (0.83-2.15) for current use vs. never used |
| Davis et al (11) | RA | 603 | Mean daily dose (none, lower or higher than 7.5mg prednisolone /day); cumulative dose in tertiles | Age, sex, smoking, year of recruitment, comorbidity, IMID activity and treatment | MI, HF, CV death | HR 1.75 (1.05-2.91) for high current dose vs. non-use; HR 1.90 (1.28-2.82) for high cumulative dose vs. non-use |
| Van Sijl et al (12) | RA | 353 | Cumulative dose (none, lower or higher than 10g prednisolone); duration of use (never, short, long) | Age, sex, estimated CV risk, IMID disease activity; disability index | IHD events, stroke/TIA | HR 1.80 (0.37-8.74) for high cumulative dose vs. non-se; HR 1.48 (0.21-10.45) for long-term use (both vs. non-use) |
| Van der Hooft et al (13) | Unsp. | 316 | Mean daily dose (none, low, or high) | Age, sex, BMI, comorbidity, CV medications | AF/flutter | HR 7.90 (4.47-13.98) for high current dose, vs. none |
| Wolfe et al (14) | RA | 17738 | Ever or current | Education, ethnicity, smoking, exercise, BMI, comorbidity, aspirin, PAS score, RA duration | MI | OR 1.4 (1.0–1.8) for ever vs. none, 1.5 (1.2–2.0) for current vs. none |

Citations are listed in the article.

Note: AF, atrial fibrillation; BMI, body mass index; CTD, connective tissue disease; CV, cardiovascular; HF, heart failure; HR, hazard ratio; IA, inflammatory arthritis; IBD, inflammatory bowel disease; IHD, ischaemic heart disease; IMID, immune-mediated inflammatory disorder; OR, odds ratio; RA, rheumatoid arthritis; SED, socio-economic deprivation; SLE, systemic lupus erythematosus; Unsp, unspecified.
