# Supplementary material for: Dose-dependent oral glucocorticoid cardiovascular risks in people with immune-mediated inflammatory diseases: A population-based cohort study
Source: PLoS Med. 2020 Dec 3;17(12):e1003432. doi: 10.1371/journal.pmed.1003432 (PMC7714202; doi:10.1371/journal.pmed.1003432)
Supplement: S1 Fig — Fig A. Flow diagram of the study cohort. Fig B. Association between time-variant oral glucocorticoid dose and incident atrial fibrillation and heart failure in patients with polymyalgia rheumatica and/or giant cell arteritis. Fig C. Association between time-variant oral glucocorticoid dose and incident acute myocardial infarction and peripheral arterial disease in patients with polymyalgia rheumatica and/or giant cell arteritis. Fig D. Association between time-variant oral glucocorticoid dose and incident cerebrovascular disease and abdominal aortic aneurysm in patients with polymyalgia rheumatica and/or giant cell arteritis. Fig E. Association between time-variant oral glucocorticoid dose and incident atrial fibrillation and heart failure in patients with rheumatoid arthritis. Fig F. Association between time-variant oral glucocorticoid dose and incident acute myocardial infarction and peripheral arterial disease in patients with rheumatoid arthritis. Fig G. Association between time-variant oral glucocorticoid dose and incident cerebrovascular disease and abdominal aortic aneurysm in patients with rheumatoid arthritis (DOCX) [file pmed.1003432.s003.docx]

##### Dose-dependent oral glucocorticoid cardiovascular risks in people with immune-mediated inflammatory diseases: a population-based cohort study

Mar Pujades-Rodriguez, Ann W Morgan, Richard M Cubbon, Jianhua Wu

S1 FIGURE

Figure A. Flow diagram of the study cohort

Figure B. Association between time variant oral glucocorticoid dose and incident atrial fibrillation and heart failure in patients with polymyalgia rheumatica and/or giant cell arteritis

Figure C. Association between time variant oral glucocorticoid dose and incident acute myocardial infarction and peripheral arterial disease in patients with polymyalgia rheumatica and/or giant cell arteritis

Figure D. Association between time variant oral glucocorticoid dose and incident cerebrovascular disease and abdominal aortic aneurysm in patients with polymyalgia rheumatica and/or giant cell arteritis

Figure E. Association between time variant oral glucocorticoid dose and incident atrial fibrillation and heart failure in patients with rheumatoid arthritis

Figure F. Association between time variant oral glucocorticoid dose and incident acute myocardial infarction and peripheral arterial disease in patients with rheumatoid arthritis

Figure G. Association between time variant oral glucocorticoid dose and incident cerebrovascular disease and abdominal aortic aneurysm in patients with rheumatoid arthritis

Note: Estimates of dose-response associations for specific types of CVD in patients with systemic lupus erythematosus are not presented because of the small numbers of events available for the analysis

Figure A. Flow chart of the study cohort

No. of people eligible for study obtained from CPRD*

**N=120,584**

History of CVD

**N=32,790**

No. of people included

**N=87,794**

No. with SLE

**N=3,950**

No. with PMR/GCA

**N=25,581**

No. with vasculitis

**N=5,199**

No. with RA

**N=25,324**

No. with IBD

**N=27,740**

No. of events

**N=6,267**

No. of events

**N=1,937**

No. of events

**N=4,236**

No. of events

**N=375**

No. of events

**N=611**

***** All eligibility study inclusion criteria except for a history of cardiovascular disease (CVD)

Note: CPRD, the Clinical Practice Research Datalink; CVD, cardiovascular diseases; IBD, inflammatory bowel disease; PRM/GCA, polymyalgia rheumatica and/or giant cell arteritis; RA, rheumatoid arthritis; SLE, systemic lupus erythematosus

**Figure B. Association between time variant oral glucocorticoid dose and incident atrial fibrillation and heart failure in patients with polymyalgia rheumatica and/or giant cell arteritis**


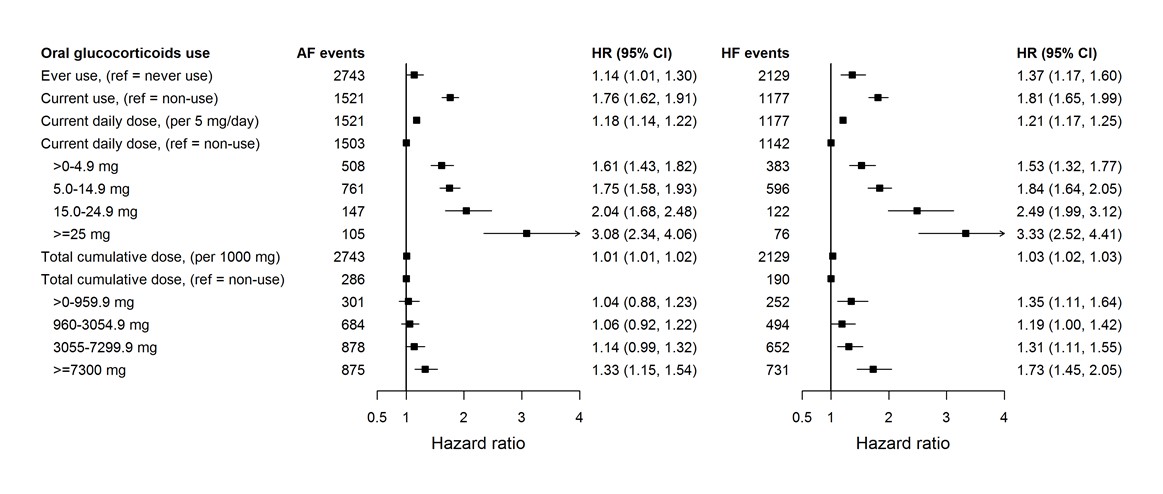


Note: AF, atrial fibrillation; CI, confidence interval; HF, heart failure; Hazard ratios (HR) from Cox proportional imputed models adjusted for baseline age, sex, index of multiple deprivation, smoking status, ethnicity, body mass index, comorbidities (diabetes, diagnosed hypertension, cancer, asthma, chronic obstructive pulmonary disease, and renal disease), biomarkers (systolic blood pressure, total cholesterol, high-density lipoprotein cholesterol, low-density lipoprotein cholesterol, c-reactive protein, creatinine), number of hospital admissions in last year, and prescribed non-oral glucocorticoids; and time-varying use of disease-modifying anti-rheumatic drugs and non-steroidal anti-inflammatory drugs; the practice identifier was included as a random intercept to account for clustering effect.

**Figure C. Association between time variant oral glucocorticoid dose and incident acute myocardial infarction and peripheral arterial disease in patients with polymyalgia rheumatica and/or giant cell arteritis**


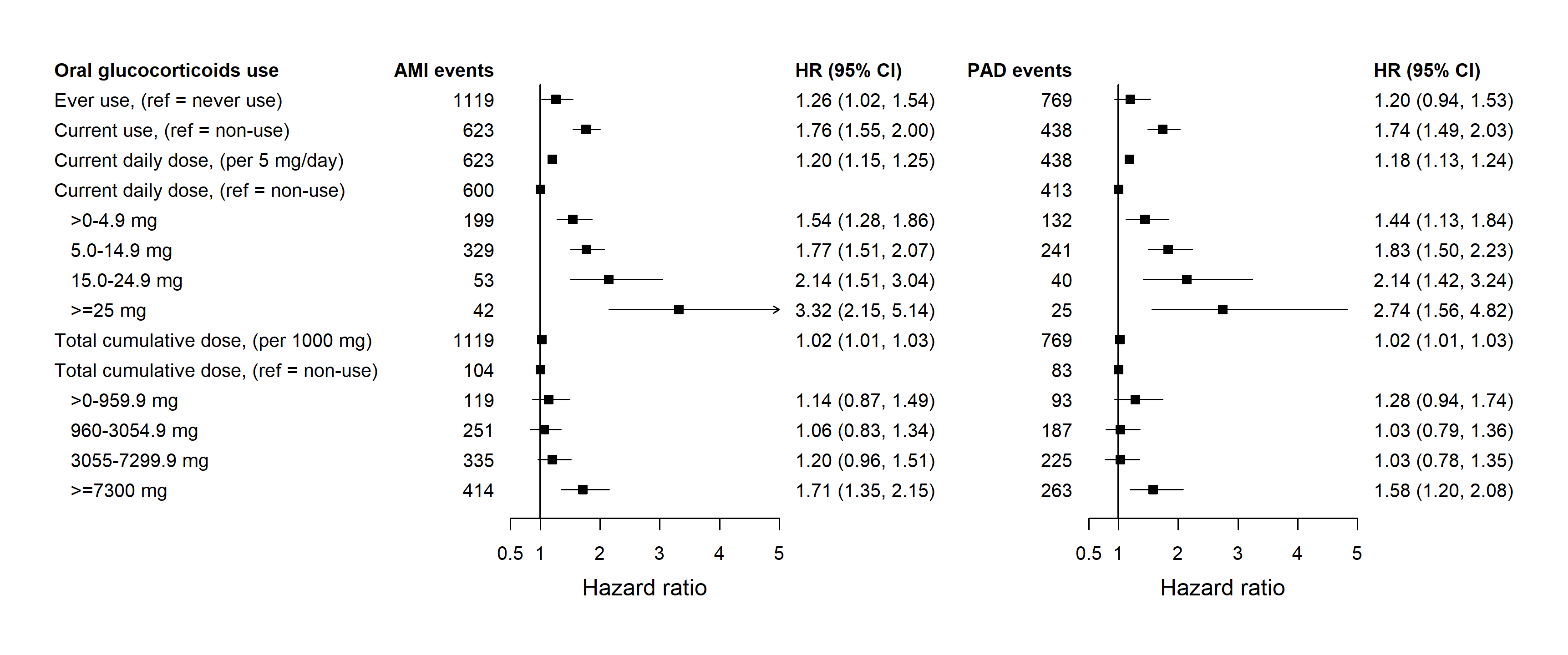


Note: AMI, acute myocardial infarction; CI, confidence interval; PAD, peripheral arterial disease, Hazard ratios (HR) from Cox proportional imputed models adjusted for baseline age, sex, index of multiple deprivation, smoking status, ethnicity, body mass index, comorbidities (diabetes, diagnosed hypertension, cancer, asthma, chronic obstructive pulmonary disease, and renal disease), biomarkers (systolic blood pressure, total cholesterol, high-density lipoprotein cholesterol, low-density lipoprotein cholesterol, c-reactive protein, creatinine), number of hospital admissions in last year, and prescribed non-oral glucocorticoids; and time-varying use of disease-modifying anti-rheumatic drugs and non-steroidal anti-inflammatory drugs; the practice identifier was included as a random intercept to account for clustering effect.

**Figure D. Association between time variant oral glucocorticoid dose and incident cerebrovascular disease and abdominal aortic aneurysm in patients with polymyalgia rheumatica and/or giant cell arteritis**


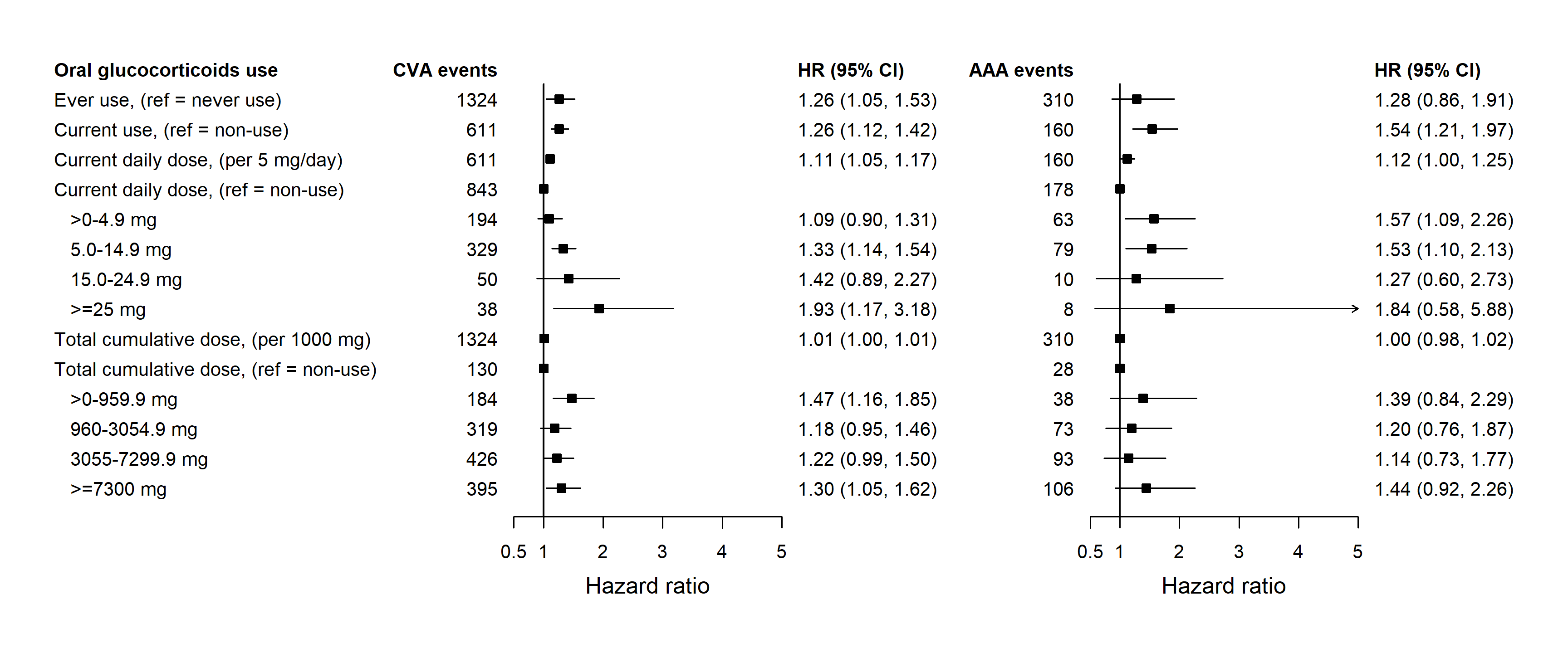


Note: AAA, abdominal aortic aneurysm; CI, confidence interval; CVA, cerebrovascular disease. Hazard ratios (HR) from Cox proportional imputed models adjusted for baseline age, sex, index of multiple deprivation, smoking status, ethnicity, body mass index, comorbidities (diabetes, diagnosed hypertension, cancer, asthma, chronic obstructive pulmonary disease, and renal disease), biomarkers (systolic blood pressure, total cholesterol, high-density lipoprotein cholesterol, low-density lipoprotein cholesterol, c-reactive protein, creatinine), number of hospital admissions in last year, and prescribed non-oral glucocorticoids; and time-varying use of disease-modifying anti-rheumatic drugs and non-steroidal anti-inflammatory drugs; the practice identifier was included as a random intercept to account for clustering effect.

**Figure E. Association between time variant oral glucocorticoid dose and incident atrial fibrillation and heart failure in patients with rheumatoid arthritis**


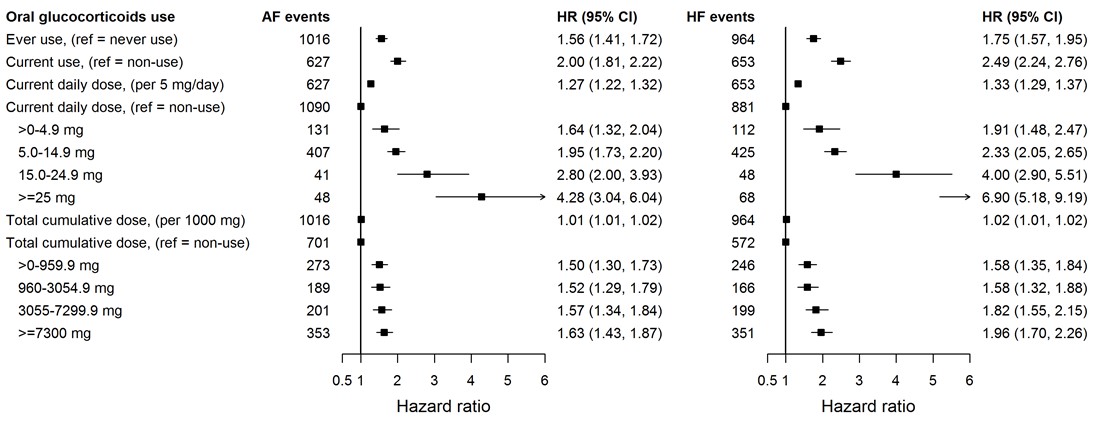


Note: AF, atrial fibrillation; CI, confidence interval; HF, heart failure. Hazard ratios (HR) from Cox proportional imputed models adjusted for baseline age, sex, index of multiple deprivation, smoking status, ethnicity, body mass index, comorbidities (diabetes, diagnosed hypertension, cancer, asthma, chronic obstructive pulmonary disease, and renal disease), biomarkers (systolic blood pressure, total cholesterol, high-density lipoprotein cholesterol, low-density lipoprotein cholesterol, c-reactive protein, creatinine), number of hospital admissions in last year, and prescribed non-oral glucocorticoids; and time-varying use of disease-modifying anti-rheumatic drugs and non-steroidal anti-inflammatory drugs; the practice identifier was included as a random intercept to account for clustering effect.

**Figure F. Association between time variant oral glucocorticoid dose and incident acute myocardial infarction and peripheral arterial disease in patients with rheumatoid arthritis**


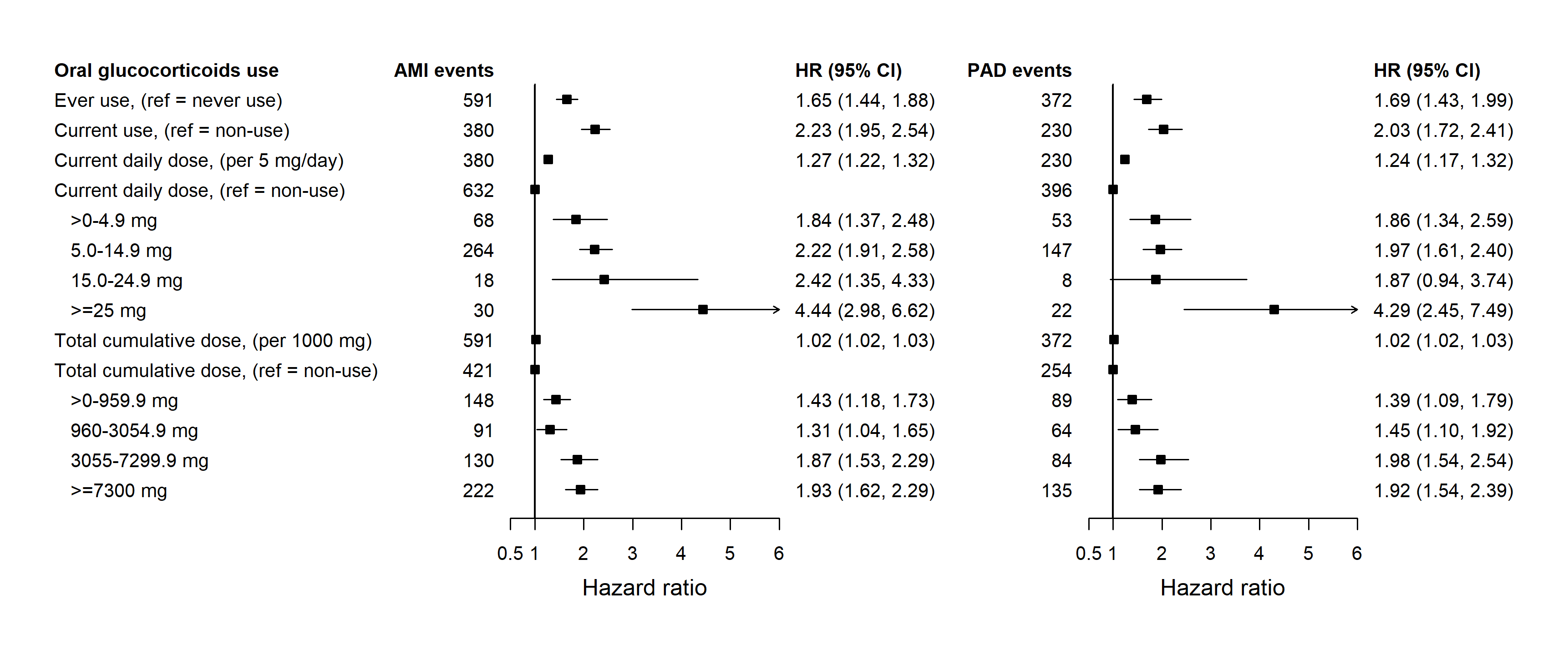


Note: AMI, acute myocardial infarction; CI, confidence interval; PAD, peripheral arterial disease. Hazard ratios (HR) from Cox proportional imputed models adjusted for baseline age, sex, index of multiple deprivation, smoking status, ethnicity, body mass index, comorbidities (diabetes, diagnosed hypertension, cancer, asthma, chronic obstructive pulmonary disease, and renal disease), biomarkers (systolic blood pressure, total cholesterol, high-density lipoprotein cholesterol, low-density lipoprotein cholesterol, c-reactive protein, creatinine), number of hospital admissions in last year, and prescribed non-oral glucocorticoids; and time-varying use of disease-modifying anti-rheumatic drugs and non-steroidal anti-inflammatory drugs; the practice identifier was included as a random intercept to account for clustering effect.

**Figure G. Association between time variant oral glucocorticoid dose and incident cerebrovascular disease and abdominal aortic aneurysm in patients with rheumatoid arthritis**


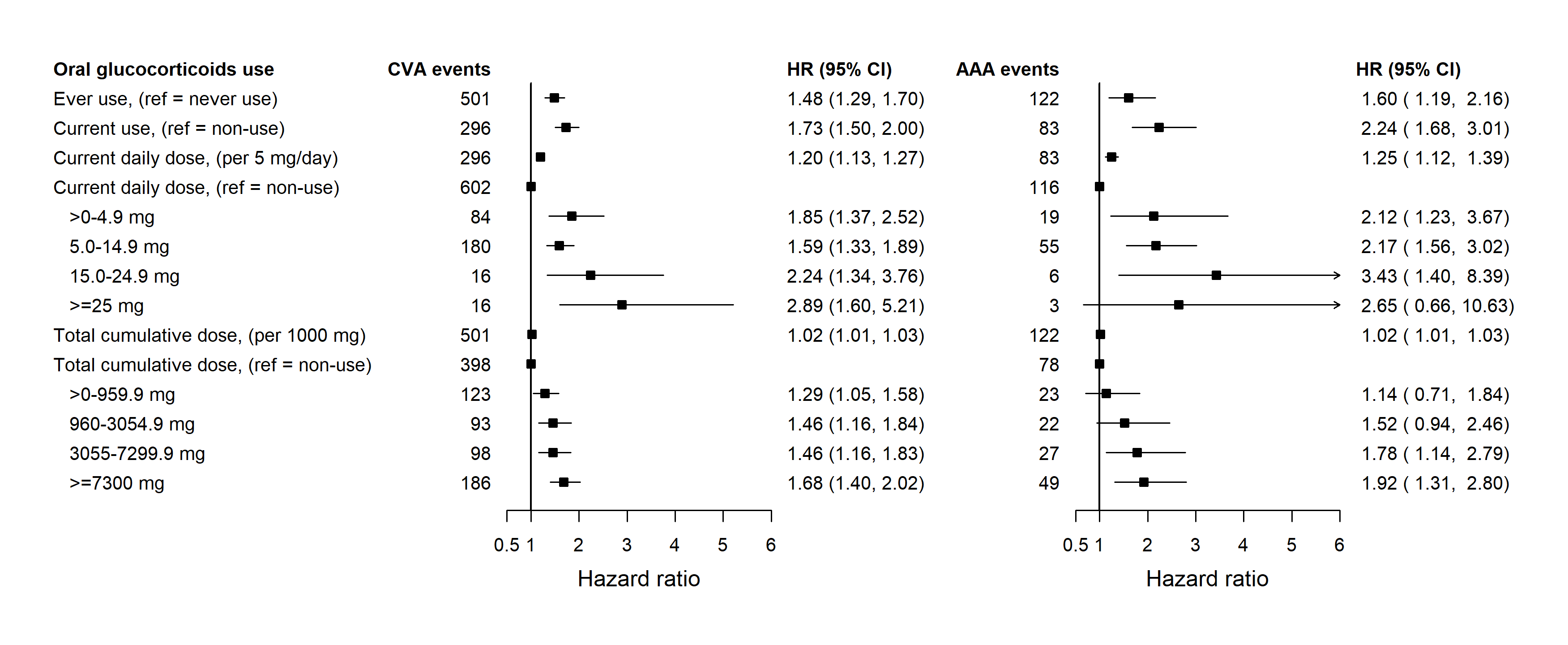


Note: AAA, abdominal aortic aneurysm; CI, confidence interval; CVA, cerebrovascular disease. Hazard ratios (HR) from Cox proportional imputed models adjusted for baseline age, sex, index of multiple deprivation, smoking status, ethnicity, body mass index, comorbidities (diabetes, diagnosed hypertension, cancer, asthma, chronic obstructive pulmonary disease, and renal disease), biomarkers (systolic blood pressure, total cholesterol, high-density lipoprotein cholesterol, low-density lipoprotein cholesterol, c-reactive protein, creatinine), number of hospital admissions in last year, and prescribed non-oral glucocorticoids; and time-varying use of disease-modifying anti-rheumatic drugs and non-steroidal anti-inflammatory drugs; the practice identifier was included as a random intercept to account for clustering effect.
