# Supplementary material for: Dose-dependent oral glucocorticoid cardiovascular risks in people with immune-mediated inflammatory diseases: A population-based cohort study
Source: PLoS Med. 2020 Dec 3;17(12):e1003432. doi: 10.1371/journal.pmed.1003432 (PMC7714202; doi:10.1371/journal.pmed.1003432)
Supplement: S2 Fig — Fig A. Association between time-variant oral glucocorticoid dose and incident atrial fibrillation and heart failure in patients with inflammatory bowel disease. Fig B. Association between time-variant oral glucocorticoid dose and incident acute myocardial infarction and peripheral arterial disease in patients with inflammatory bowel disease. Fig C. Association between time-variant oral glucocorticoid dose and incident cerebrovascular disease and abdominal aortic aneurysm in patients with inflammatory bowel disease. Fig D. Association between time-variant oral glucocorticoid dose and incident atrial fibrillation and heart failure in patients with vasculitis. Fig E. Association between time-variant oral glucocorticoid dose and incident acute myocardial infarction and peripheral arterial disease in patients with vasculitis. Fig F. Association between time-variant oral glucocorticoid dose and incident cerebrovascular disease and abdominal aortic aneurysm in patients with vasculitis. (DOCX) [file pmed.1003432.s004.docx]

##### Dose-dependent oral glucocorticoid cardiovascular risks in people with immune-mediated inflammatory diseases: a population-based cohort study

Mar Pujades-Rodriguez, Ann W Morgan, Richard M Cubbon, Jianhua Wu

S2 FIGURE

Figure A. Association between time variant oral glucocorticoid dose and incident atrial fibrillation and heart failure in patients with inflammatory bowel disease

Figure B. Association between time variant oral glucocorticoid dose and incident acute myocardial infarction and peripheral arterial disease in patients with inflammatory bowel disease

Figure C. Association between time variant oral glucocorticoid dose and incident cerebrovascular disease and abdominal aortic aneurysm in patients with inflammatory bowel disease

Figure D. Association between time variant oral glucocorticoid dose and incident atrial fibrillation and heart failure in patients with vasculitis

Figure E. Association between time variant oral glucocorticoid dose and incident acute myocardial infarction and peripheral arterial disease in patients with vasculitis

Figure F. Association between time variant oral glucocorticoid dose and incident cerebrovascular disease and abdominal aortic aneurysm in patients with vasculitis

Note: Estimates of dose-response associations for specific types of CVD in patients with systemic lupus erythematosus are not presented because of the small numbers of events available for the analysis

**Figure A. Association between time variant oral glucocorticoid dose and incident atrial fibrillation and heart failure in patients with inflammatory bowel disease**


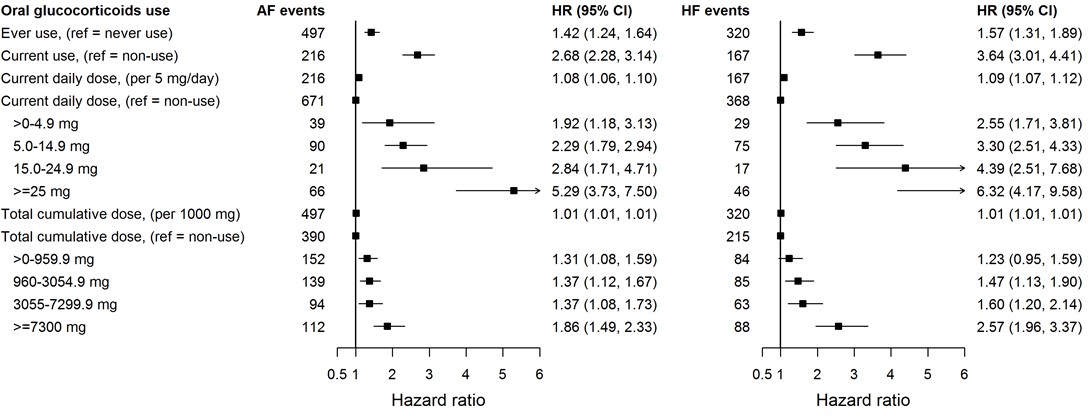


Note: AF, atrial fibrillation; CI, confidence interval; HF, heart failure. Hazard ratios (HR) from Cox proportional imputed models adjusted for baseline age, sex, index of multiple deprivation, smoking status, ethnicity, body mass index, comorbidities (diabetes, diagnosed hypertension, cancer, asthma, chronic obstructive pulmonary disease, and renal disease), biomarkers (systolic blood pressure, total cholesterol, high-density lipoprotein cholesterol, low-density lipoprotein cholesterol, c-reactive protein, creatinine), number of hospital admissions in last year, and prescribed non-oral glucocorticoids; and time-varying use of disease-modifying anti-rheumatic drugs and non-steroidal anti-inflammatory drugs; the practice identifier was included as a random intercept to account for clustering effect.

**Figure B. Association between time variant oral glucocorticoid dose and incident acute myocardial infarction and peripheral arterial disease in patients with inflammatory bowel disease**


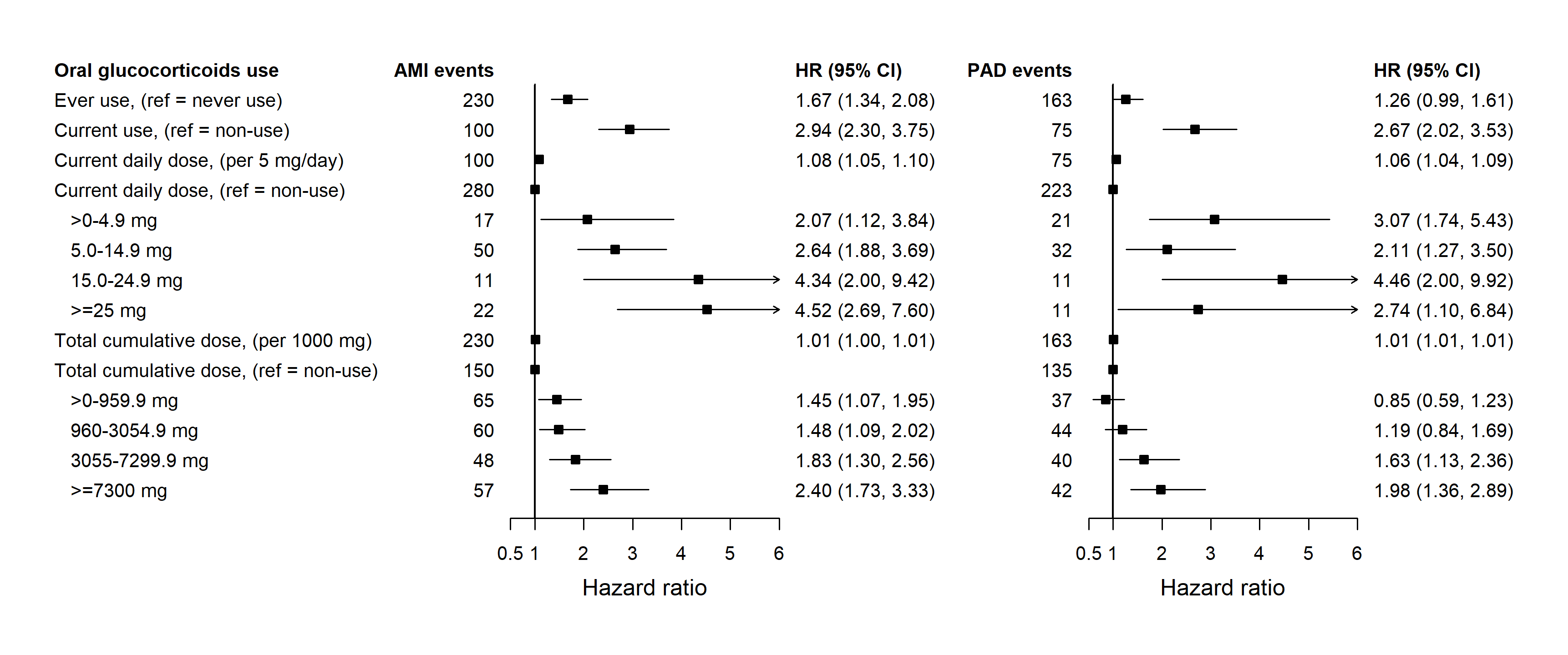


Note: AMI, acute myocardial infarction; CI, confidence interval; PAD, peripheral arterial disease. Hazard ratios (HR) from Cox proportional imputed models adjusted for baseline age, sex, index of multiple deprivation, smoking status, ethnicity, body mass index, comorbidities (diabetes, diagnosed hypertension, cancer, asthma, chronic obstructive pulmonary disease, and renal disease), biomarkers (systolic blood pressure, total cholesterol, high-density lipoprotein cholesterol, low-density lipoprotein cholesterol, c-reactive protein, creatinine), number of hospital admissions in last year, and prescribed non-oral glucocorticoids; and time-varying use of disease-modifying anti-rheumatic drugs and non-steroidal anti-inflammatory drugs; the practice identifier was included as a random intercept to account for clustering effect.

**Figure C. Association between time variant oral glucocorticoid dose and incident cerebrovascular disease and abdominal aortic aneurysm in patients with inflammatory bowel disease**


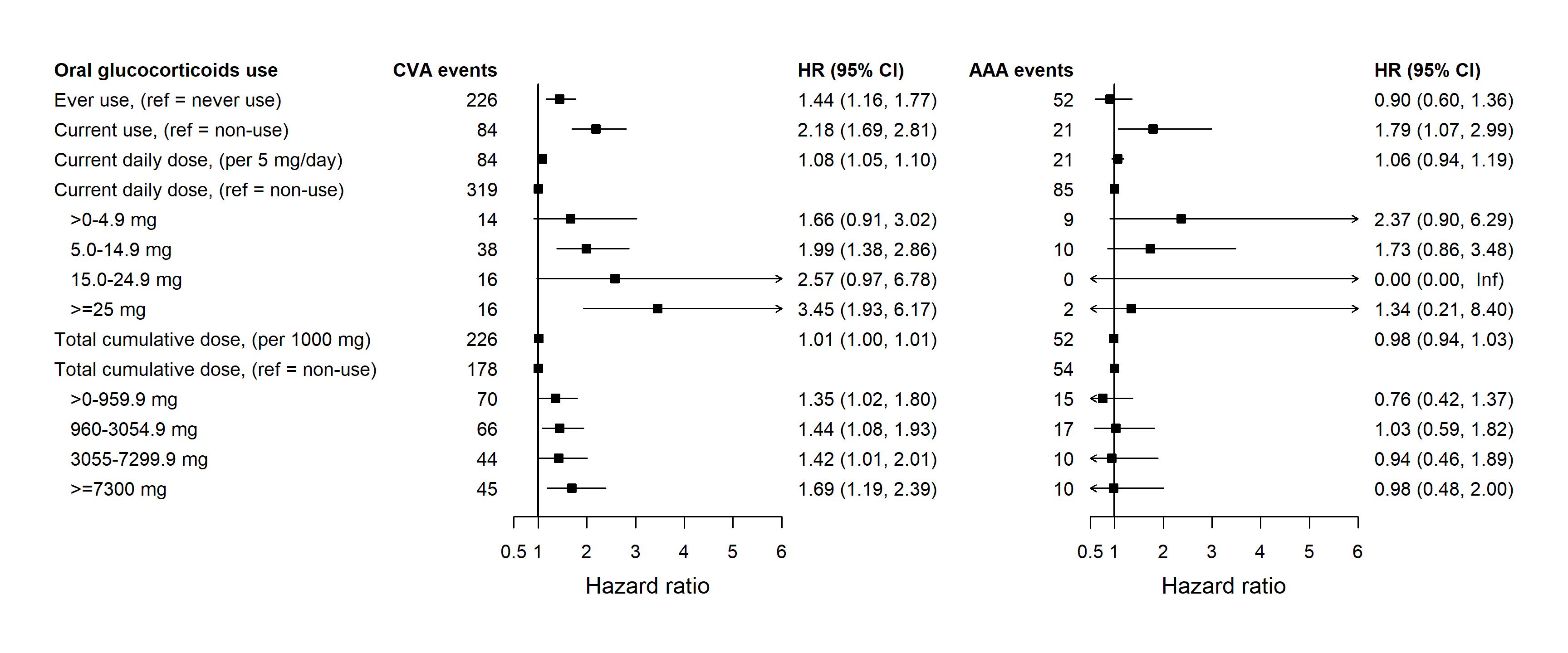


Note: AAA. Abdominal aortic aneurysm; CI, confidence interval; CVA, cerebrovascular disease. Hazard ratios (HR) from Cox proportional imputed models adjusted for baseline age, sex, index of multiple deprivation, smoking status, ethnicity, body mass index, comorbidities (diabetes, diagnosed hypertension, cancer, asthma, chronic obstructive pulmonary disease, and renal disease), biomarkers (systolic blood pressure, total cholesterol, high-density lipoprotein cholesterol, low-density lipoprotein cholesterol, c-reactive protein, creatinine), number of hospital admissions in last year, and prescribed non-oral glucocorticoids; and time-varying use of disease-modifying anti-rheumatic drugs and non-steroidal anti-inflammatory drugs; the practice identifier was included as a random intercept to account for clustering effect.

**Figure D. Association between time variant oral glucocorticoid dose and incident atrial fibrillation and heart failure in patients with vasculitis**

**
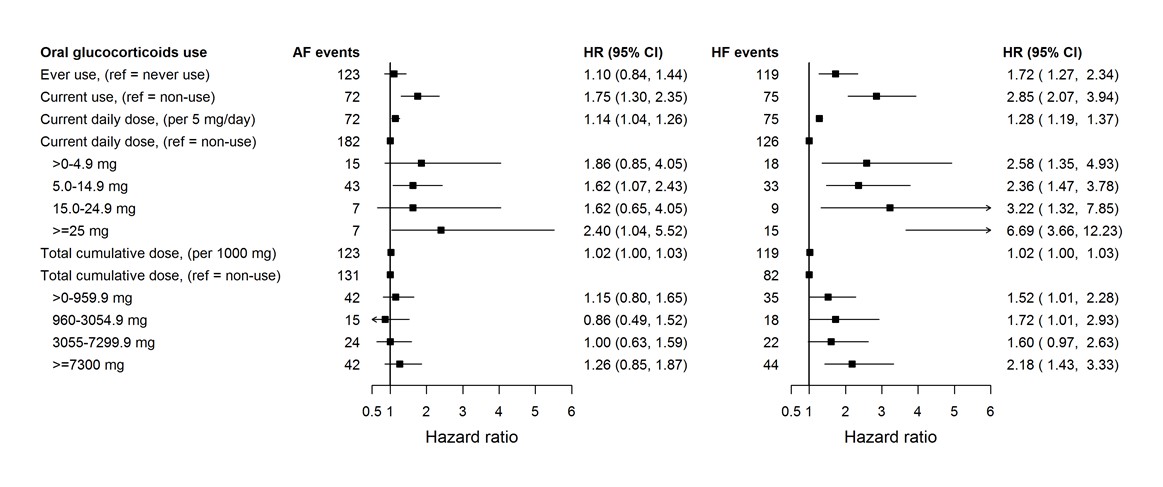
**

Note: AF, atrial fibrillation; CI, confidence interval; HF, heart failure. Hazard ratios (HR) from Cox proportional imputed models adjusted for baseline age, sex, index of multiple deprivation, smoking status, ethnicity, body mass index, comorbidities (diabetes, diagnosed hypertension, cancer, asthma, chronic obstructive pulmonary disease, and renal disease), biomarkers (systolic blood pressure, total cholesterol, high-density lipoprotein cholesterol, low-density lipoprotein cholesterol, c-reactive protein, creatinine), number of hospital admissions in last year, and prescribed non-oral glucocorticoids; and time-varying use of disease-modifying anti-rheumatic drugs and non-steroidal anti-inflammatory drugs; the practice identifier was included as a random intercept to account for clustering effect.

**Figure E. Association between time variant oral glucocorticoid dose and incident acute myocardial infarction and peripheral arterial disease in patients with vasculitis**


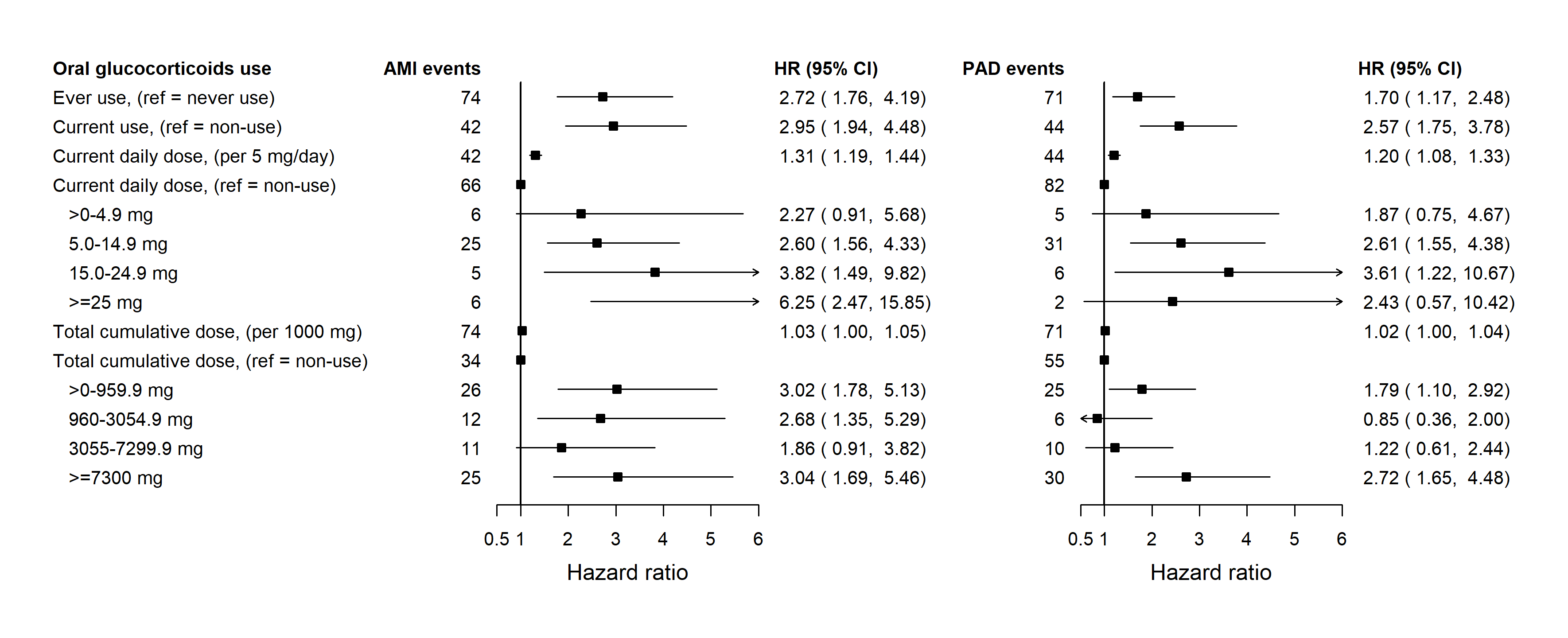


Note: AMI, acute myocardial infarction; CI, confidence interval; PAD, peripheral arterial disease. Hazard ratios (HR) from Cox proportional imputed models adjusted for baseline age, sex, index of multiple deprivation, smoking status, ethnicity, body mass index, comorbidities (diabetes, diagnosed hypertension, cancer, asthma, chronic obstructive pulmonary disease, and renal disease), biomarkers (systolic blood pressure, total cholesterol, high-density lipoprotein cholesterol, low-density lipoprotein cholesterol, c-reactive protein, creatinine), number of hospital admissions in last year, and prescribed non-oral glucocorticoids; and time-varying use of disease-modifying anti-rheumatic drugs and non-steroidal anti-inflammatory drugs; the practice identifier was included as a random intercept to account for clustering effect.

**Figure F. Association between time variant oral glucocorticoid dose and incident cerebrovascular disease and abdominal aortic aneurysm in patients with vasculitis**


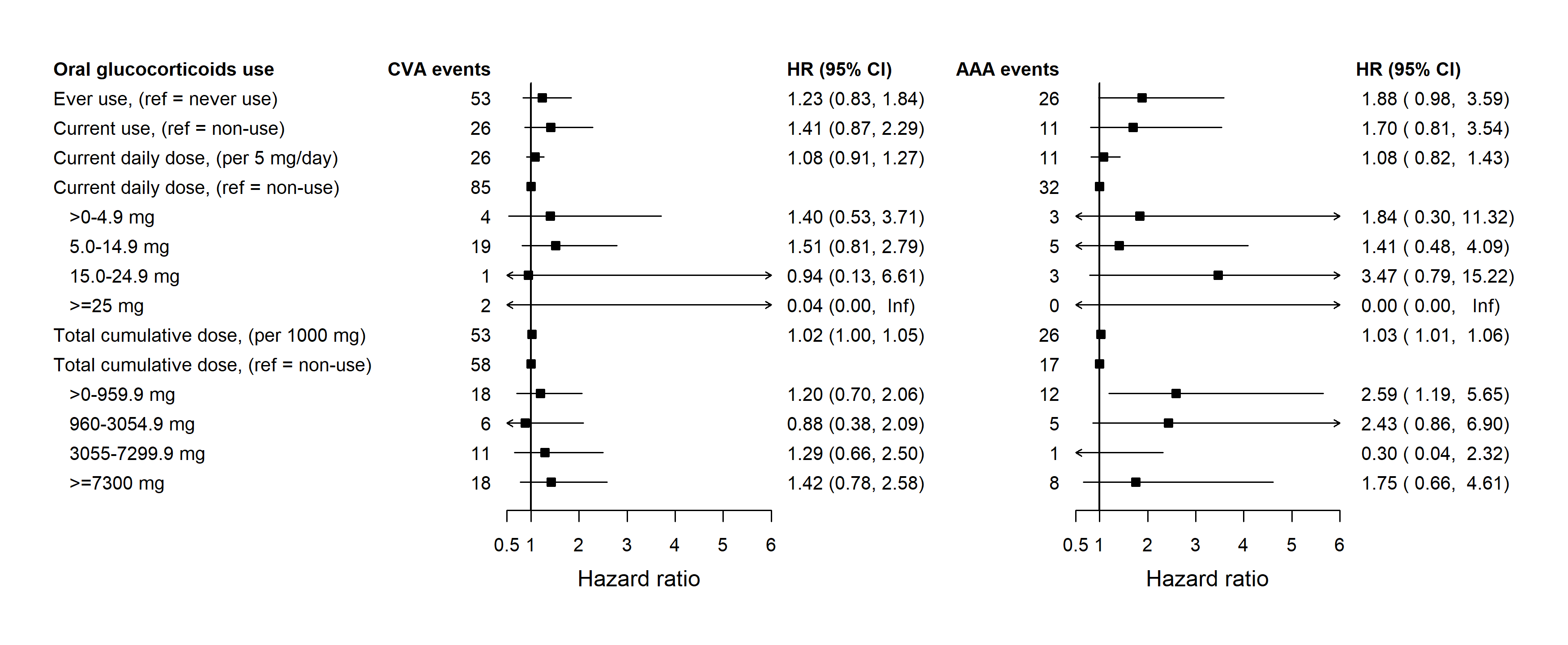


Note: AAA, abdominal aortic aneurysm; CI, confidence interval; CVA, cerebrovascular disease. Hazard ratios (HR) from Cox proportional imputed models adjusted for baseline age, sex, index of multiple deprivation, smoking status, ethnicity, body mass index, comorbidities (diabetes, diagnosed hypertension, cancer, asthma, chronic obstructive pulmonary disease, and renal disease), biomarkers (systolic blood pressure, total cholesterol, high-density lipoprotein cholesterol, low-density lipoprotein cholesterol, c-reactive protein, creatinine), number of hospital admissions in last year, and prescribed non-oral glucocorticoids; and time-varying use of disease-modifying anti-rheumatic drugs and non-steroidal anti-inflammatory drugs; the practice identifier was included as a random intercept to account for clustering effect.
