# Supplementary material for: Dose-dependent oral glucocorticoid cardiovascular risks in people with immune-mediated inflammatory diseases: A population-based cohort study
Source: PLoS Med. 2020 Dec 3;17(12):e1003432. doi: 10.1371/journal.pmed.1003432 (PMC7714202; doi:10.1371/journal.pmed.1003432)
Supplement: Attachment — Submitted filename: Response_PMEDICINE-D-19-02196.docx [file pmed.1003432.s006.docx]

PMEDICINE-D-19-02196l

Leeds, 5 August 2020

RE: Dose-dependent oral glucocorticoid cardiovascular risk in people with immune-mediated inflammatory diseases

Dear Dr McBride,

Thank you for reviewing our manuscript investigating the incidence of cardiovascular diseases in people diagnosed with chronic inflammatory diseases. We appreciate the thoughtful reviews of the referees that will help to strengthen this important research. We have summarised our response to their comments below.

Please note that I will be away, without internet access, between 12^th^ and 22^nd^ August 2020. For urgent matters during this period you can contact my co-author Jianhua Wu.

**EDITORS**

**1. We'd suggest revising the title into PLOS Medicine's usual style, this should have the study design (eg "A randomized controlled trial," "A retrospective study," "A modelling study," etc.) in the subtitle after a colon.**
Thank you. We have amended the title as requested:

“Dose-dependent oral glucocorticoid cardiovascular risk in people with immune-mediated inflammatory diseases***: a retrospective cohort study***”

**2. Please redraft parts of the abstract so that this uses complete sentence structure rather than fragments (ie, "**WE AIMED** to quantify glucocorticoid dose-dependent cardiovascular risk.." etc).**
Thank you. We have edited the abstract accordingly.

**3. In the last sentence of the Abstract Methods and Findings section, please include a brief summary of any key limitations of the study's methodology.**
We have added the following information:

“***The lack of hospital medication records and drug adherence data, might have led to underestimation of the dose prescribed when specialists provided care and overestimation of the dose taken during periods of low disease activity. The resulting dose misclassification in some patients is likely to have reduced the size of dose-response estimates.***”

**4. At this stage, we ask that you include a short, non-technical Author Summary of your research to make findings accessible to a wide audience that includes both scientists and non-scientists. The Author Summary should immediately follow the Abstract in your revised manuscript.**
This has been added.

**5. The authors might like to consider using an appropriate reporting guideline to enhance the detail of reporting of key aspects of the study methods and findings. Options might include RECORD, RECORD-PE. If doing so the completed reporting guideline checklist should be appended as a supporting information file with the revised paper.**
We have completed the RECORD-PE checklist and have submitted the document with the revised paper as a supporting information file.

**6. Please clarify whether the analysis reported here corresponds to one laid out in a prospective protocol or analysis plan? Please state this (either way) early in the Methods section.**

a) If a prospective analysis plan (from your funding proposal, IRB or other ethics committee submission, study protocol, or other planning document written before analyzing the data) was used in designing the study, please include the relevant prospectively written document with your revised manuscript as a Supporting Information file to be published alongside your study, and cite it in the Methods section. A legend for this file should be included at the end of your manuscript.

b) If no such document exists, please make sure that the Methods section transparently describes when analyses were planned, and when/why any data-driven changes to analyses took place. 

**c) In either case, changes in the analysis-- including those made in response to peer review comments-- should be identified as such in the Methods section of the paper, with rationale.**
This analysis is part of a broader research program aiming at quantifying glucocorticoid dose-response for different types of adverse events, including CVD, hypertension (doi: 10.1503/cmaj.191012), diabetes (10.1136/bmjdrc-2020-001220), infections (10.1503/cmaj.190178), etc. A generic protocol was written and approved by the Independent Scientific Advisory Committee for Medicines and Healthcare products Regulatory Agency database research (ISAC), reference 16_146. No specific protocol was written for the analyses of CVD risk. All analyses included in the submission were planned. Only the supplementary analyses requested by the reviewers were unplanned. As requested, we have now documented this in the Methods.

“***All the analyses were planned except for the sensitivity analyses additionally performed in response to peer review comments to test the robustness of the primary analyses. These were: 1) consider longer periods of prescribing prior to the index date (i.e. 2 years and 5 years) to control for protopathic bias (as CVD is associated both with drug exposure and the indication for the exposure); 2) propensity score adjustment of Cox proportional hazard models to balance covariates between exposure groups, in order to control for residual confounding by indication.***”

Comments from the academic editor:

**I would also like the authors to discuss the quantitative difference between some of the earlier works and this paper (perhaps in a supplementary table). In addition, given that one of the key rationales for this paper is incomplete confounder adjustment in previous papers, it might be good to provide a table reporting effects by cumulative adjustment of various confounders. Does it make any difference?**
Thank you for the comment. We have added a paragraph in the Discussion to do this and a new table in the supplementary document.

“***A summary of the methodology and major findings of previous studies is presented in our data supplement (Table 22 in S1 File). This illustrates that our study: is substantially larger than most published work; offers estimates of risk for previously undefined categories of CVD events (e.g. abdominal aortic aneurysm, peripheral arterial disease); includes previously neglected immune-mediated inflammatory diseases (e.g. giant cell arteritis); and provides more detailed quantification of glucocorticoid use. Where analysis groups are similar, the 95% confidence interval of our estimates overlap with those reported in previous studies in most cases, although our intervals are smaller due to the larger size of our cohort.***”

**REVIEWER 1**

I have reviewed the statistical methods and the models used are appropriate, using multivariate Cox models and KM techniques. The authors had also conducted a complete case analysis and compared this to multiple imputation which is good practice. However, there are some suggestions/comments I have on the study design.

***MAJOR:***

**1. Relating to exposure period of only one year prior to index date and residual confounding, in pharmaco-epidemiological studies, the major issue here is consideration for protopathic bias and confounding by indication.  In this study, neither issue is sufficiently investigated for or considered in the study design. See** [**https://www.ncbi.nlm.nih.gov/pmc/articles/PMC4594717/**](https://nam04.safelinks.protection.outlook.com/?url=https%3A%2F%2Fwww.ncbi.nlm.nih.gov%2Fpmc%2Farticles%2FPMC4594717%2F&data=02%7C01%7Cmpujadesrodriguez%40uk.imshealth.com%7C4796ad38f04a4f64e8d208d81ec6653e%7C5989ece0f90e40bf9c791a7beccdb861%7C1%7C0%7C637293185204634499&sdata=A%2BEavqafIIBC96969Q2SaFRDJpk5KHdVo%2Bi1slt%2Bti0%3D&reserved=0) **Firstly, to control for protopathic bias (as the outcome CVD here is associated both with the exposure and the indication for the exposure, symptoms or diagnosis itself, usually lag time can be added to exposure period (exclusion of any drug prescribing in the year before the index date). Often times, additional sensitivity analyses are also incorporated at longer exclusions periods for prescribing prior to the index date (e.g. > 2 years or > 5 years). This allows testing of the robustness of the primary results. If indeed, there is a dose-response relationship, then this effect should remain in the lag-time analyses. The present analyses uses only an exposure period of one year prior to diagnosis.**
As requested, we have performed additional sensitivity analyses considering longer exclusions periods for prescribing prior to the start of follow-up: 2 years and 5 years. These analyses only affected two glucocorticoid exposure variables: glucocorticoid ever use and total cumulative dose. We present the results of these analyses in Supplemental Table 20. Estimates for ever use were slightly increased with higher number of years of exposure pre-index date and did not change for total cumulative dose. The Methods and Results sections have been amended accordingly.

“We defined several time-variant glucocorticoid variables to quantify current and cumulative drug exposure: i) ever use from one year ***(2 or 5 years in additional sensitivity analyses)*** prior to follow-up start (binary variable)... iv) cumulative dose since one year ***(2 or 5 years in additional sensitivity analyses)*** prior to follow-up start per 1,000 mg…”

“***In additional sensitivity analyses performed in response to peer reviewer comments, we found slightly higher dose-response estimates for glucocorticoid ever use when longer lengths of glucocorticoid exposure before the start of follow-up were considered and similar estimates for cumulative dose (Table 20 in S1 File).***”

**2. Related to this, confounding by indication will therefore also be a potential issue here as well. The indication for prescriptions itself (diagnosis of inflammatory diseases) is related to the outcome of CVD here. This is going to be the case here as the inflammatory diseases are known to be associated with an increased risk of CVD, with more severe symptoms also potentially being treated more aggressively. This creates residual confounding which is not appropriately taken into account. To account for methods which can minimize this: (1) using a propensity scores to adjust the statistical models - a statistical way to try to achieve balance between exposure groups in observational research designs or (2) using a comparator group where the indication for the prescription is not related to the outcome (i.e. a disease group which would be prescribed oral glucocorticoids). Covariate adjustment as completed in this analysis adjusts for each individual variable as a potential confounder but not achieve as robust adjustments for residual confounding as in propensity score adjustments (the results can differ based on these methods). See** [**https://www.bmj.com/content/347/bmj.f6409.full**](https://nam04.safelinks.protection.outlook.com/?url=https%3A%2F%2Fwww.bmj.com%2Fcontent%2F347%2Fbmj.f6409.full&data=02%7C01%7Cmpujadesrodriguez%40uk.imshealth.com%7C4796ad38f04a4f64e8d208d81ec6653e%7C5989ece0f90e40bf9c791a7beccdb861%7C1%7C0%7C637293185204644493&sdata=VtWIknaMGgXe8rjH%2FaOs1ojlixfM6X28vDErPIEW7NQ%3D&reserved=0)**. These additional analyses would test the robustness of the primary results and should be considered. In general (especially using observational or real-world evidence data), the burden is up to the researchers to provide additional analyses to test the robustness of the results under various assumptions. The study here in my opinion is incomplete as issues related to residual confounding have not been considered.** 
Thank you for the suggestion. To investigate the issue of confounding by indication, we generated a propensity score to predict the indication for prescription and adjusted the Cox model accordingly. We present the results of this analysis in Supplemental Table 21. The size of all the estimates remained unchanged after propensity score adjustment. The Methods and Results sections have been amended accordingly.

“***We also found materially unchanged estimates after further adjusting for propensity scores for glucocorticoid prescribing (Table 21 in S1 File).***”

**3. Eligibility of patients: It's not clear patients or mentioned if patients have more than one inflammatory condition which group they would fall under - I would have to presume that the groups are not mutually exclusive (i.e. an individual with co-morbid inflammatory conditions) could be contributing to more than one category. In the sub-analyses of each disease, presenting the results separately is not an issue, but in the combined analysis, presumably this could potentially double count individuals who may fall into more than one category? Is this is the case, then overall effect estimates would could potentially be exaggerated. Please clarify?**
A total of 5543 (6.3%) patients had more than one chronic inflammatory condition diagnosed and contributed to more than one of the disease specific analyses. In the combined analyses, each individual was only counted once. Models were adjusted for each inflammatory condition as separate binary variables, to take into account presence of co-morbid inflammatory conditions. We have clarified this issue in the Methods.

“***For the combined analyses, patients were assigned to the immune-mediated inflammatory disease group corresponding to the earliest condition diagnosed in the database.***”

**4. Outcomes: Cause-specific incidence outcomes are fine but combining composite CVD outcomes presents an issue of competing risk events (as the individuals may have multiple types of events). Using a cumulative incidence model would be more helpful when presenting the overall composite outcome.**
We thank both reviewers for raising the issue of competing risk and the possible overestimation of cumulative incidence with Kaplan-Meier estimates. We have now applied a cumulative incidence function to estimate the cumulative incidence for the composite CVD and the cause-specific cardiovascular outcomes, including death as one of the competing risk events as well. We have updated the cumulative incidences estimates in Supplemental Tables 5-7 and have also updated the Methods accordingly.

***MINOR:***

**Lines 109: It's not clear here what the authors mean by follow-up started when they "first became eligible". Is this the date which first instance of a diagnosis of any of the six immune-mediated inflammatory disease? Please clarify**
Thank you for pointing out the lack of clarity of the sentence. Eligibility criteria included continuous registration in a CPRD practice for 1 year or more, age ≥18 years, free of CVD and diagnosis with one of the 6 inflammatory conditions of interest. The Methods has been clarified as follows:

“This was a cohort study including all patients continuously registered in a CPRD practice for 1 year or more, aged ≥18 years and free of CVD, who had been diagnosed with at least one of 6 immune-mediated inflammatory diseases commonly treated with oral glucocorticoids at or before the start of follow-up ***(inclusion eligibility criteria)***. These were polymyalgia rheumatica, giant cell arteritis (GCA), systemic lupus erythematosus, rheumatoid arthritis, vasculitis, and inflammatory bowel disease (Figure 1 in S1 File). Diagnostic codes used to identify patients with each immune-mediated inflammatory disease are shown in Table 1 in S1 File. For each patient, the follow-up started when they first became eligible ***(i.e. earliest date on which all the inclusion criteria were met).***”

**Line 120: Conversion to prednisolone-equivalent dose.  Another common way to standardise the different drug dosages is to use the WHO DDD as the benchmark. See** [**https://www.whocc.no/atc_ddd_index/?code=H02AB**](https://nam04.safelinks.protection.outlook.com/?url=https%3A%2F%2Fwww.whocc.no%2Fatc_ddd_index%2F%3Fcode%3DH02AB&data=02%7C01%7Cmpujadesrodriguez%40uk.imshealth.com%7C4796ad38f04a4f64e8d208d81ec6653e%7C5989ece0f90e40bf9c791a7beccdb861%7C1%7C0%7C637293185204644493&sdata=p0I4MZL3EppI%2ByHzFasY8g8Fscbrul62RhmC7pK5TN8%3D&reserved=0)**. Many pharmaco-epidemiology studies use this approach for comparability. Perhaps authors could consider this as a sensitivity analyses.**
Previous studies based on the analysis of primary care electronic health records in the UK used the same approach that we have implemented in the study. Our results can therefore be compared to those previously published on this UK database. In order to perform the proposed analysis we would need to completely recalculate the daily dose for the patient cohort and effectively repeat all the analyses again in full. Currently, we do not have capacity to perform this additional analysis in a timely manner and we would prefer to leave this excellent suggestion for a future publication.

**Limitation: limitation of CPRD data should be mentioned - covers only prescribing in general practice and does not cover any in-hospital prescribing associated with any acute events during the exposure or follow-up periods.**

This has been added to the abstract and it is discussed as a imitations in the Discussion (page 23).

**REVIEWER 2**

**This is a large population-based cohort study that examines glucocorticoid dose-dependent cardiovascular risk in six immune-mediated diseases. The study adds important knowledge in terms of dose-dependent associations and absolute risks. Further, the study has more sufficient confounder control than many prior observational studies and has made substantial efforts to disentangle confounding by indication and disease severity. The study is very comprehensive, however, I have comments that need to be addressed.**  
Thank you for the positive feedback.

***ABSTRACT***

**Comment #1:**

**Page 2, lines 28-30:
"Evidence for the association between glucocorticoid dose and cardiovascular risk is weak for moderate and low doses. To quantify glucocorticoid dose-dependent cardiovascular risk in people with six immune-mediated inflammatory diseases." It is difficult to understand if it is the evidence or the associations that are weak. Further, you may add "This study aimed" to the second sentence.** 
Thank you for the comment. We have amended this paragraph to clarify the text:

“***It is uncertain whether or not low to moderate glucocorticoid dose increases cardiovascular risk. We aimed to*** quantify glucocorticoid dose-dependent cardiovascular risk in people with six immune-mediated inflammatory diseases.”

**Comment #2
Page 2, lines 43-46.
"We found strong dose-dependent estimates for all immune-mediated diseases (hazard ratio [HR] for <5.0mg daily dose vs. non-use=1.74, 95%CI: 1.64-1.84; range 1.52 for polymyalgia rheumatic and/or giant cell arteritis to 2.82 for systemic lupus erythematosus), all cardiovascular outcomes, regardless of disease activity level."** **This sentence is difficult to follow, you may consider to re-write**.

Thank you for pointing this out. We have amended the paragraph as follows:

“***Compared to people not currently using glucocorticoids, those receiving <5.0mg daily prednisolone-equivalent dose had increased all-cause CVD risk*** (hazard ratio [HR]=1.74; 95%CI 1.64-1.84; range 1.52 for polymyalgia rheumatica and/or giant cell arteritis to 2.82 for systemic lupus erythematosus). ***Increased dose-dependent risk ratios were found regardless of disease activity level and for all type-specific CVDs, with the highest ratios seen for*** heart failure and acute myocardial infarction.”

***INTRODUCTION*
Comment #3
Page 4, lines 58-60:
"Patients with immune-mediated inflammatory diseases often receive long-term courses of oral glucocorticoids to reduce disease activity and inflammation during the initial episode and subsequent episodic flares." I suggest to delete the word "long-term" as it is not correct for all six diseases and also it depends on the definition of long-term treatment.   It is accurate that long-term oral glucocorticoid treatment is indicated for polymyalgia rheumatica and giant cell arthritis. However, for rheumatoid arthritis long-term oral glucocorticoid treatment is rarely used and only if DMARDs cannot be used. Oral short-term/ medium-term glucocorticoid treatment may be used until effect of DMARDs.**    
Thank you for your comment. We agree that long-term use of glucocorticoids is not currently recommended for most patients with rheumatoid arthritis in clinical guideline and have deleted ‘long-term’ from the sentence. However, please note that the reality of prescribing in the UK is different, and we have recently submitted work demonstrating that in 2017, 16% of patients with rheumatoid arthritis in the UK received ≥90 days per year total prescription duration (19% during the first year following diagnosis).

**Comment #4
Page 4, lines 61- 65. The first sentence is in present tense ("can") and the last is in past tense ("could").** 
We have amended the later sentence to also use present tense.

**Comment #5
Page 4, lines 79-81:
"Our study aimed to estimate daily and cumulative dose-dependent oral glucocorticoid cardiovascular disease risk accurately in people diagnosed with six common immune-mediated** **inflammatory diseases in England."
I understand that the word "accurately" refers to the time-variant measure of the exposure, but I would delete/replace the word as it is may also refer to the quality of your exposure data as validity and completeness.**  
Thank you. We agree with the reviewer and have amended this sentence.

“Our study aimed to estimate daily and cumulative dose-dependent oral glucocorticoid cardiovascular disease risk ***accurately*** in people diagnosed with six common immune-mediated inflammatory diseases in England***, using time-dependent regression methods***.”

***METHODS*
Comment #6
How was the distribution of the different generic types of oral glucocorticoids, i.e. the frequency of prednisolone use, betamethasone use etc.?**
We did not consider the distribution of the different generic types of oral glucocorticoids in the analysis. Some individuals may have several types of glucocorticoids over the long period of follow up. Nevertheless, 96.4% of prescriptions issued to the patients were for prednisolone. Furthermore, we converted different types of glucocorticoids into prednisolone-equivalent dose to account for the differences between drugs.

We have now added a footnote to Supplemental Table 2 providing information about the proportion of prednisolone prescriptions issued to the patient cohort.

**Comment #7
From Table 2, S1 File it reads as if budesonide was included as an oral glucocorticoid? The main actions are in the mucosa (i.e. locally acting) and the bioavailability is < 20%. I would suggest not to include budesonide as an oral glucocorticoid exposure in your analyses or examine in a sensitivity analysis if not including budesonide as an exposure changes your estimates.** 
Thank you for this comment. Only 0.2% of all prescriptions issued for oral glucocorticoids were for budesonide. This should therefore not have an impact on the estimates.

We have also added this information in the footnote of Supplemental Table 2.

**Comment #8
In Table 1 age and biomarkers are described by mean (SD). Are they normal distributed? Else, please change this to e.g. median.**
We have checked the normality of the continuous variables in table 1 and revised mean (SD) to median (interquartile range) if they were not normally distributed.

**Comment #9
Were the biomarkers included as continuous variables in the adjusted models and if so, how were they modelled (i.e. linear, cubic spline etc.)** 
Biomarkers were included as continuous variables and modelled as cubic spline in the adjusted models. We have revised the Methods to reflect this.

**Comment #10
Page 7, lines 161-162.
"We estimated cumulative probabilities of CVD outcomes using Kaplan-Meier methods."
The cumulative incidences/absolute risks of CVD were estimated using the Kaplan-Meier estimator. As you have competing risk by death, please take this into account by computing the cumulative incidence function (e.g. the Aalen Johansen estimator) instead of the Kaplan-Meier function. The Kaplan-Meier function likely overestimates the cumulative incidence in competing risk settings (reference: Lacny et al. Kaplan-Meier survival analysis overestimates cumulative incidence of health-related events in competing risk settings: a meta-analysis. Journal of Clinical Epidemiology 93 (2018) 2535).**
We have addressed this comment in response to reviewer 1. We applied a cumulative incidence function to estimate the cumulative incidence and updated the results in the Supplemental Tables 5-7.

***RESULTS*
Comment #11
In Table 1 you have provided count and percentage of DMARDs ever during follow-up, which is relevant since you model this as a time-varying variable. Would it be relevant to do the same for NSAIDs?**
We included both DMARDs and NSAIDs as time-varying variables in the model. We mentioned that in the ‘confounding variables’ subsection of the Methods.

**Comment #12
How many were loss to follow up (left the family practice)?**
There were 20,851/87,794 (23.7%) patients who left their CPRD practice. The follow-up of these patients was censored on the date in which they left the practice. We have added this information in the Results.

“***A total of 20,851 (23.7%) patients transferred out of their general practices and were lost to follow up.***”

**Comment #13
Your absolute risk estimates are important from a clinical point of view, hence, I think some of the results from e.g. Table 5, S1 File deserve to be in the main text as a Table or Figure (if space enough and after re-estimating them in accordance with comment #7).**    
Thank you for pointing this out. We agree that these estimates are important. Current Supplemental Table 5 is big, so we have left this in the Supplement file and have added a subset of key results in the main article.

**Comment #14
It would be appropriate to show crude hazard ratios also (maybe just in the supplementary)** 
We include now Supplemental Table 19 providing the crude hazard ratios corresponding to the primary analyses presented in Table 2.

***DISCUSSION***

**Comment #15
Page 21:
"We adjusted estimates of risk for established cardiovascular risk factors (e.g. smoking, hypertension and diabetes), concomitant use of medications (e.g. time-variant disease-modifying antirheumatic drugs, non-steroidal antiinflammatory drugs, non-oral glucocorticoid use)"
The estimates of risk (absolute risk) were unadjusted, so please delete "estimates of risk" or replace with another term.**

We have amended the sentence to clarify what hazard ratios were adjusted.

**Comment #16
It would strengthen the paper if estimates of e.g. PPV and completeness for the immune-mediated inflammatory diseases and cardiovascular outcomes were included in the discussion.**
Estimates of PPV reported in published validation studies (≥75%) and appropriate references of published studies reporting on completeness of recording for the diseases of interest are now provided in the discussion.
